# Probiotic Supplementation for Promotion of Growth in Children: A Systematic Review and Meta-Analysis

**DOI:** 10.3390/nu14010083

**Published:** 2021-12-25

**Authors:** Joseph Catania, Natasha G. Pandit, Julie M. Ehrlich, Muizz Zaman, Elizabeth Stone, Courtney Franceschi, Abigail Smith, Emily Tanner-Smith, Joseph P. Zackular, Zulfiqar A. Bhutta, Aamer Imdad

**Affiliations:** 1 College of Medicine, SUNY Upstate Medical University, Syracuse, NY 13210, USA; cataniaj@upstate.edu (J.C.); panditn@upstate.edu (N.G.P.); ehrlichj@upstate.edu (J.M.E.); zamanm@upstate.edu (M.Z.); estone121@gmail.com (E.S.); cfranceschi94@gmail.com (C.F.); 2 Health Science Library, SUNY Upstate Medical University, Syracuse, NY 13210, USA; smithab@upstate.edu; 3 College of Education, University of Oregon, Eugene, OR 97403, USA; etanners@uoregon.edu; 4 Department of Pathology, Children’s Hospital of Philadelphia, University of Pennsylvania, Philadelphia, PA 19104, USA; Joseph.Zackular@pennmedicine.upenn.edu; 5 Centre for Global Child Health, The Hospital for Sick Children, Toronto, ON M5G 0A4, Canada; zulfiqar.bhutta@aku.edu; 6 Center of Excellence in Women and Child Health, The Aga Khan University, Karachi 74800, Pakistan; 7 Department of Pediatrics, Division of Pediatric Gastroenterology, Hepatology and Nutrition, SUNY Upstate Medical University, Syracuse, NY 13210, USA

**Keywords:** probiotics, synbiotics, growth, low- and middle-income countries, high-income countries, systematic review

## Abstract

Probiotics are commonly prescribed to promote a healthy gut microbiome in children. Our objective was to investigate the effects of probiotic supplementation on growth outcomes in children 0–59 months of age. We conducted a systematic review and meta-analysis which included randomized controlled trials (RCTs) that administered probiotics to children aged 0–59 months, with growth outcomes as a result. We completed a random-effects meta-analysis and calculated a pooled standardized mean difference (SMD) or relative risk (RR) and reported with a 95% confidence interval (CI). We included 79 RCTs, 54 from high-income countries (HIC), and 25 from low- and middle-income countries (LMIC). LMIC data showed that probiotics may have a small effect on weight (SMD: 0.26, 95% CI: 0.11–0.42, grade-certainty = low) and height (SMD 0.16, 95% CI: 0.06–0.25, grade-certainty = moderate). HIC data did not show any clinically meaningful effect on weight (SMD: 0.01, 95% CI: −0.04–0.05, grade-certainty = moderate), or height (SMD: −0.01, 95% CI: −0.06–0.04, grade-certainty = moderate). There was no evidence that probiotics affected the risk of adverse events. We conclude that in otherwise healthy children aged 0–59 months, probiotics may have a small but heterogenous effect on weight and height in LMIC but not in children from HIC.

## 1. Introduction

The role of gut microbiota in human health has been studied extensively in the recent past [1]. Observational and experimental studies from community settings have shown that gut microbiota immaturity or dysbiosis is associated with risk of development of acute malnutrition and linear growth failure in children [2,3,4,5]. Probiotics are one of the commonly used products to promote healthy gut microbiomes, encompassing a USD 54 billion industry around the globe [6,7]. Probiotics are defined as live microorganisms that, when administered in adequate amounts, confer a health benefit to the host [8]. Prebiotics are a non-digestible food ingredient that benefit the host by stimulating the growth or activity of microorganisms indigenous to the human digestive tract [9]. Synbiotics are a combination of both probiotics and prebiotics [10]. Multiple studies have assessed the usage of probiotics in the context of childhood growth [11,12,13]. A systematic review by Onubi et al. assessed the effect of probiotics on growth in children in developing countries [12]. This review of 12 studies was published in 2014 and did not include studies from high-income countries and did not use a standard method to assess the overall quality of evidence. We, therefore, aimed to systematically assess up-to-date evidence on the effects of probiotics supplementation on growth outcomes in children aged 0–59 months by following the methodological guidance of the Cochrane Collaboration.

## 2. Materials and Methods

We conducted a systematic review and meta-analysis and reported our findings according to Preferred Reporting Items for Systematic Reviews and Meta-Analyses (PRISMA) guidelines. We developed a team consensus on the study questions and methods in a protocol before the start of the study and registered this protocol on PROSPERO (CRD42020154352).

We included individual- and cluster-randomized trials. Trials with multiple treatment arms were included if the only difference between the arms was probiotic usage. We included studies with children aged 0 to 59 months who were supplemented with oral probiotics or synbiotics. We excluded studies that only tested prebiotics. As our review considered both probiotics and synbiotics, the term “probiotic” hereafter refers to both probiotics and synbiotics. The studies were included irrespective of dose, dosage forms, and strain of probiotics. We included studies that had an adequate comparison group such as standard-of-care, placebo, or no-intervention groups. We excluded observational studies such as cohort studies, case–control studies, case series, and case reports. We excluded studies on children with congenital abnormalities, syndromic diagnosis, and chronic conditions such as cystic fibrosis or inflammatory bowel disease. Furthermore, we excluded studies in which the authors declared that participants were already undernourished or malnourished.

We searched multiple electronic databases, including PubMed, Embase, Cochrane Central Register of Controlled Trials, CINAHL, Scopus, and LILACS. The last date of search was 6 November 2020. The search strategy for all databases is available in the supplementary document (Text S1, supplementary document). We searched ClinicalTrials.gov for ongoing studies. We also searched the reference sections of published studies and systematic reviews. We contacted the authors in relevant fields for any new studies. If growth data were measured but not reported in the study paper, we contacted the authors for those results. Trial registries were searched and checked for unpublished data as well as newly published data. If a study was only available in a language other than English, we attempted to translate the paper. If an adequate translation was unavailable, the study was excluded.

At least two authors screened titles and abstracts using Covidence software [14], and extracted data in duplication (AI, JC, NP, JE, MZ ES, CF). We double extracted relevant data using a data collection form specifically designed for this review. Data were extracted independently into the same form. We extracted the data for study design, study setting (hospital vs. community, country, country income status), inclusion and exclusion criteria, participant characteristics (age, nutritional status, gender), and characteristics of intervention (type, strain, form, duration, frequency, dose, comparison group). We included data on all outcomes at the longest follow-up reported by individual studies. If longest follow-up was not reported, we used suitable data the study provided which was either in the form of total growth gain over the course of the study or growth gain per time. Any duplicate data were only counted once. Any disagreement among the authors during any stage of the study was resolved by discussion and review of the publication(s) with consultation of the senior author (AI).

Our primary outcomes included weight-for-age (continuous outcome, Z scores or kg) and height-for-age (continuous outcome, cm or Z scores). Growth data were used in the form of Z scores as to WHO standards or in the primary units. Secondary outcomes included weight-for-height, BMI, head circumference, and adverse events such as nausea, vomiting, diarrhea, abdominal pain, flatulence, and sepsis.

We pooled the dichotomous outcomes to obtain a summary estimate in the form of mean relative risk (RR) and reported with its 95% confidence interval (CI). We used the standardized mean difference effect size for continuous outcomes due to studies reporting data in different units (e.g., a few studies reported weight in kg and the others in Z scores) and reported the standardized mean effect with its 95% CI. We used Review Manager 5.4 and Stata to conduct the meta-analysis [15,16]. We used the random-effects models to pool data as the effect of probiotics could be different in different study populations. We used funnel plots and Egger’s test to assess for publication bias. 

For eligible studies with multiple treatment arms, one eligible pair was selected and included, and if more than two groups were eligible, they were combined into a single pairwise comparison. If a trial had multiple arms that addressed different doses, those arms were combined and compared with the control arm to avoid double counting the control arm in the synthesis. Cluster trials were synthesized together with individually randomized trials using cluster adjusted values. If trial results were not cluster adjusted, we adjusted the result by methods given in the Cochrane handbook [17]. Extended details of data input are found in the supplementary document (Text S2). 

We assessed clinical, methodological, and statistical heterogeneity of effects reported in the literature. Statistical heterogeneity was assessed using the χ^2^ test, I^2^, and tau-squared statistics, and visual inspection of the forest plot. We considered statistical heterogeneity to be significant if the *p* value of the χ^2^ was <0.1, I^2^ values were above 50%, and forest plots showed different magnitude and effect of the intervention. We conducted subgroup analyses to explore reasons for any substantial statistical heterogeneity.

We conducted all analyses for low- and middle-income countries (LMIC) separately from high-income countries (HIC), given presumed heterogeneity in study populations across these settings. A subgroup analysis was conducted for growth outcomes using a χ^2^ test to assess whether the effects of probiotics were significantly different for the following subgroups: age: 0–<6 months vs. 6–<60 months, probiotic interventions with single vs. multiple strains, synbiotics vs. probiotics, and participant status of healthy vs. premature/low birth weight. The healthy group included participants not defined as premature/low birth weight. As previously described, we excluded all studies in which authors declared that participants were undernourished or malnourished. We also completed a post hoc analysis in which we calculated the effect of each strain, or combination of strains, for each of our main outcomes. 

We conducted sensitivity analyses by excluding studies with high risk of bias, those studies where data were supplied in a form other than mean (SD), or when effect-size data were extracted from figures. 

Two authors assessed and agreed upon bias using the Cochrane Collaboration’s risk-of-bias tool-2 (ROB-2) for assessing risk of bias for all outcomes from the included studies in the meta-analysis [18]. Using this tool, results for each outcome were judged as either low, some concerns, or high risk of bias. The certainty of overall evidence for the effect of probiotics for an outcome was assessed using the Grading of Recommendations Assessment, Development and Evaluation (GRADE) method [19]. We present the results of the quality assessment in the form of a summary-of-findings table separately for high-income countries and low- and middle-income countries. 

## 3. Results

### 3.1. Literature Search 

**Figure 1:**
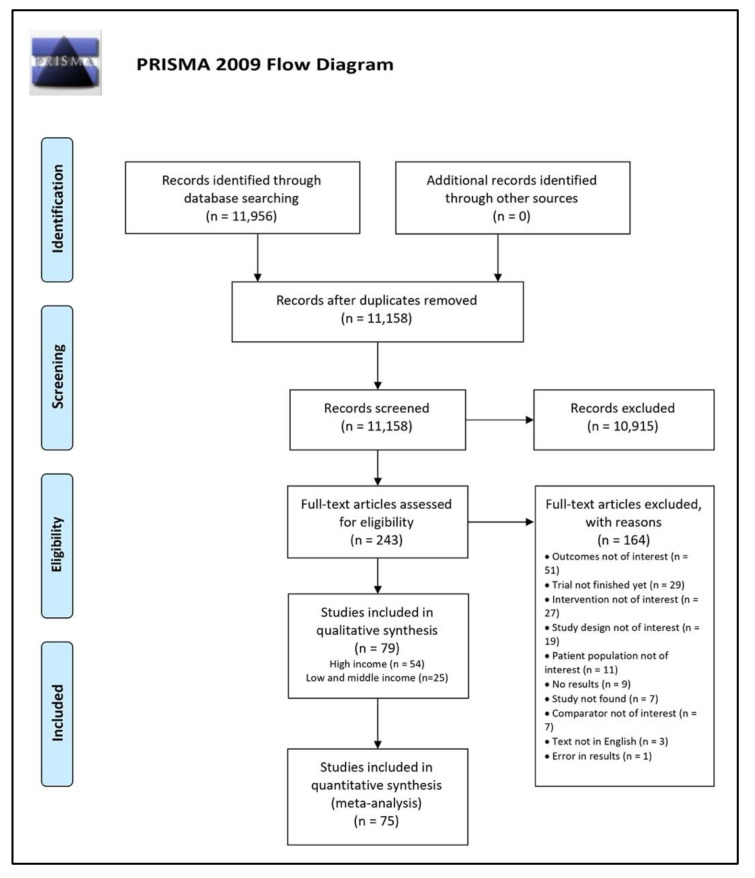
PRISMA Flow Diagram.

Our literature search identified 11,158 titles after exclusion of duplicates. Figure 1 shows the results of the literature search. The 11,158 studies were reduced to 243 full-text studies after applying the exclusion criteria stated in our methods section. After screening the full text of 243 studies, we ultimately included 79 studies in our systematic review, of which 54 studies were from high-income countries, and 25 studies from low- and middle-income countries [20,21,22,23,24,25,26,27,28,29,30,31,32,33,34,35,36,37,38,39,40,41,42,43,44,45,46,47,48,49,50,51,52,53,54,55,56,57,58,59,60,61,62,63,64,65,66,67,68,69,70,71,72,73,74,75,76,77,78,79,80,81,82,83,84,85,86,87,88,89,90,91,92,93,94,95,96,97,98]. We excluded 164 studies, and reasons for exclusion can be found in Table S1. 

### 3.2. Characteristics of Included Studies

Tables S2–S5 in the supplementary document display participant characteristics and intervention characteristics, respectively. The included studies had 12,524 total participants from high-income countries and 13,037 total participants from low- and middle-income countries. The median sample size for included studies was 149 with a range of 4541 (min: 15, max: 4556). Seventy-six studies were individually randomized [20,21,22,23,24,25,26,27,28,29,30,31,32,33,34,35,36,37,38,39,40,41,42,43,44,45,46,47,48,49,50,51,52,53,54,55,56,57,58,59,60,61,62,63,64,65,66,67,68,69,70,71,72,73,74,75,77,78,79,80,82,83,84,85,86,87,88,89,90,91,92,94,95,96,97,98], and three studies were cluster-randomized and were already cluster-adjusted [76,81,93]. Thirty-six of the studies were conducted in the community setting [21,22,24,25,27,28,30,37,43,45,48,52,53,54,56,58,60,63,64,67,70,71,73,74,76,77,78,80,86,87,88,89,93,98], and 43 were conducted in a hospital setting [20,23,26,29,31,32,33,34,35,36,38,39,40,41,42,44,46,47,50,51,55,57,59,61,62,65,66,68,69,72,75,79,82,83,84,85,90,91,92,95,96,97]. A total of 35 countries were represented in our meta-analysis and most of the studies were conducted in the United States [23,30,43,67,71,74,82,94,98] (more details in Text S3 in Supplementary document). Twelve studies had multiple intervention arms that we combined to obtain a single pairwise comparison [21,31,37,48,54,56,58,67,82,88,89]. Two studies were used as two separate datasets as they contained two independent treatment and control arms [33,84]. Twenty-one studies used an intervention that consisted of synbiotics [20,22,31,33,39,42,43,49,60,61,63,64,66,70,73,78,82,87,93,96,98]. Fifty-two studies were conducted on apparently healthy participants [20,21,22,24,25,27,28,30,31,33,37,40,41,43,45,46,48,49,50,52,53,54,55,56,58,60,61,62,63,64,66,67,70,71,73,74,76,77,78,79,80,83,85,86,87,88,89,91,93,94,96,98], and twenty-seven studies were conducted on premature or low birth-weight infants [23,26,29,32,34,35,36,38,39,42,44,47,51,57,59,65,68,69,72,75,81,82,84,90,92,95,97]. Thirty studies compared a probiotic intervention to a placebo [21,23,24,25,32,34,35,37,39,44,45,46,47,49,52,56,59,60,61,65,72,77,79,81,82,84,90,91,97,98], forty-eight studies compared to standard of care [20,22,26,27,28,29,30,31,33,36,38,40,41,42,43,48,50,51,53,54,55,57,58,62,63,64,66,67,68,69,70,71,73,74,75,76,78,80,83,85,86,87,88,89,93,94,95,96], and one study compared to no intervention [92]. The most common single-strain intervention used was *Bifidobacterium lactis* [33,39,59,64,70,73,75,83,85,94]. The median dose was 1.0 × 10^9^ CFUs (range 1.0 × 10^6^–1.8 × 10^10^) administered per day of study. The median duration of intervention was 13 weeks (range 1–104 weeks) for studies that reported an average duration of intervention. Thirty-two studies had a probiotic intervention that consisted of multiple strains [21,23,24,26,28,29,31,37,42,44,47,48,49,51,54,56,58,65,66,67,68,80,82,84,87,88,89,91,93,95,96,98], forty-six studies had a probiotic intervention that consisted of a single strain [20,22,25,30,32,33,34,35,36,38,39,40,41,43,45,46,50,52,53,55,57,59,60,61,62,63,64,69,70,71,72,73,74,75,76,77,78,79,81,83,85,86,90,92,94,97], and one study did not specify the probiotic content of the intervention. A total of 46 studies received industry funding [22,24,28,30,31,33,40,41,43,44,45,46,50,52,53,54,55,56,57,59,62,63,64,67,70,71,72,73,74,75,76,78,80,81,83,84,85,86,87,88,89,90,93,96,97,98]. At least one growth outcome was listed as a primary outcome in 38 studies [20,22,23,27,30,31,33,34,36,40,41,42,43,44,52,54,56,58,59,63,66,67,70,71,74,77,78,82,83,84,85,86,87,88,92,94,96,97].

### 3.3. LMIC Results

#### 3.3.1. Weight-for-Age

**Figure 2:**
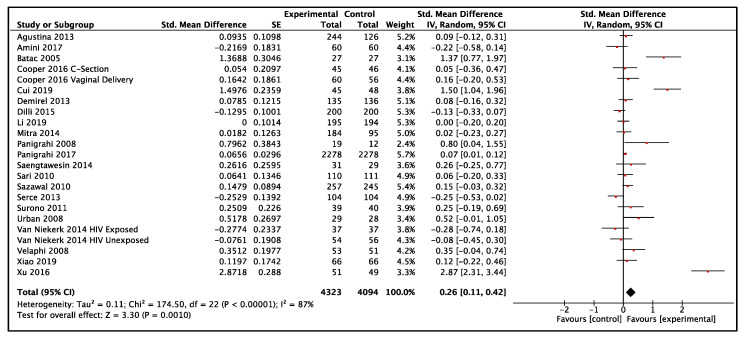
Effect of use of probiotics on weight-for-age in children 0–59 months of age from LMIC [21,26,27,33,36,38,39,50,58,60,61,68,69,70,72,77,83,84,85,91,92].

**Figure 3:**
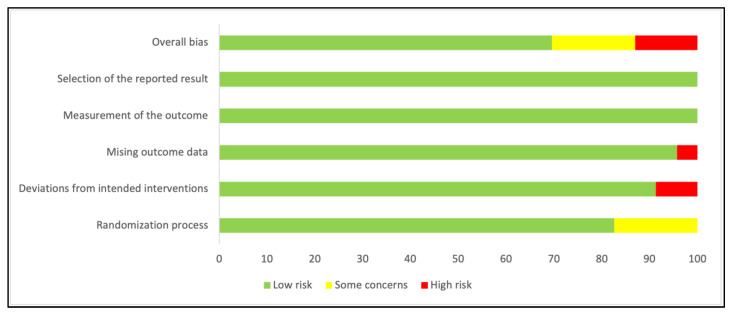
Risk of bias for weight-for-age in LMIC. The figure shows the five domains of the risk of bias 2.0 tool and the overall bias. The horizontal axis depicts percentage of studies. Most of the studies were at low risk of bias.

**Figure 4:**
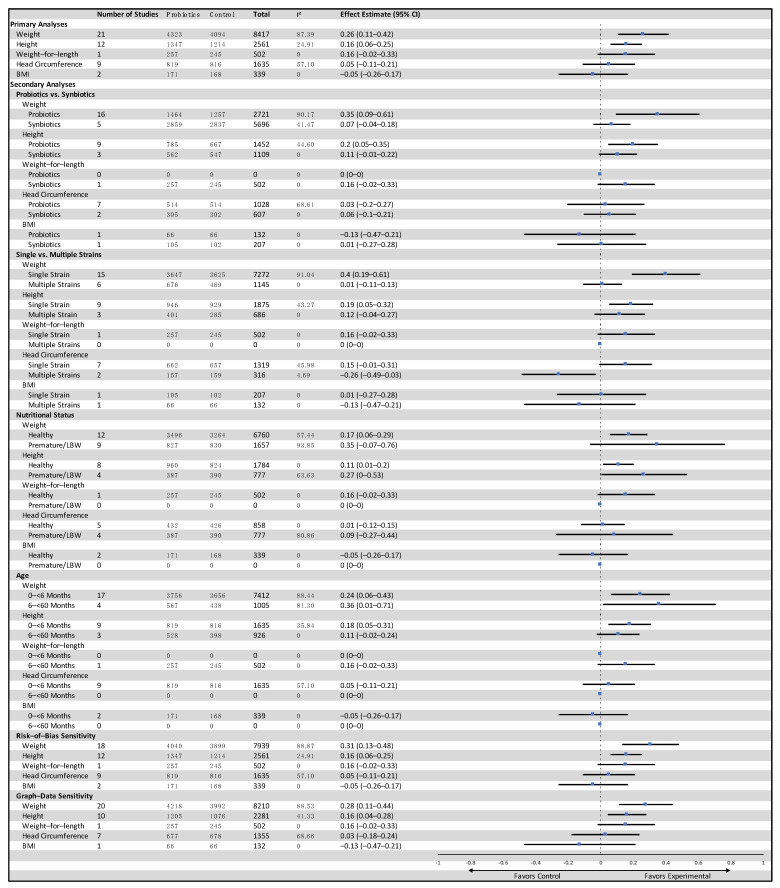
Primary and subgroup analyses for the effects of probiotics and growth outcomes in children from low- and middle-income countries.

In LMIC, twenty-one studies reported data on weight-for-age and included a total of 8417 participants (4323 probiotics, 4094 control) [21,26,27,33,36,38,39,50,58,60,61,68,69,70,72,77,83,84,85,91,92]. The results showed low-certainty evidence that probiotics had a small effect on weight when compared to the control group (SMD: 0.26, 95% CI: 0.11–0.42, *p* = 0.001, I^2^ = 87%, Figure 2). The GRADE evidence was downgraded to low due to statistical heterogeneity in the pooled data and clinical heterogeneity in the use of probiotics in the included studies (Table 1). The funnel plot and Egger’s test showed no evidence of publication bias (*p* = 0.07, Funnel plot in Figure S1). The summary risk of bias for weight-for-age in low- and middle-income countries is shown in Figure 3. The results were similar without high-risk-of-bias studies (SMD 0.31, 95% CI: 0.13–0.48, *p* < 0.001, I^2^ = 89%). A subgroup analysis comparing single versus multiple strain interventions found that single-strain interventions had a greater impact on weight gain than multiple strain interventions (P_subgroup_ = 0.002). Figure 4 shows the results of other sensitivity analyses and subgroup analyses. All other subgroup and sensitivity analyses did not show significantly different results for weight-for-age. A post hoc subgroup analysis based on type of strains did not show a particular individual strain or combination of strains that were effective in terms of effect on weight (Table S6).

#### 3.3.2. Height-for-Age

**Figure 5:**
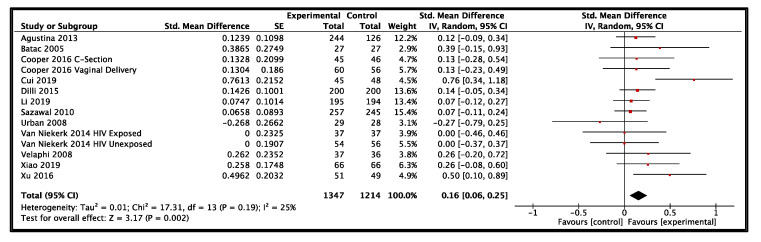
Effect of use of probiotics on height-for-age in children 0–59 months of age from low- and middle-income countries [21,27,33,36,39,50,70,83,84,85,91,92].

Twelve studies from LMIC reported data on height-for-age and included a total of 2561 participants (1347 probiotics, 1214 control) [21,27,33,36,39,50,70,83,84,85,91,92]. The results provided moderate-certainty evidence that probiotics had a small effect on height-for-age when compared to the control group (SMD: 0.16, 95% CI: 0.06–0.25, *p* = 0.002, I^2^ = 25%, Figure 5). The GRADE evidence was downgraded due to heterogeneity in the clinical use of probiotics in the included studies (Table 1). The funnel plot and Egger’s test showed no evidence of publication bias (*p* = 0.25). All subgroup and sensitivity analyses did not show significantly different results for height-for-age (Figure 4). A summary risk of bias for height-for-age and other outcomes in LMIC are available in Figures S6–S9 of the supplementary document.

#### 3.3.3. Other Outcomes

Among studies from LMIC, probiotics did not have any significant effect on other outcomes including adverse events (forest plots in Figures S10–S15). A funnel plot for head circumference in LMIC is available in Figure S3. The other analyses for LMIC, including subgroup and sensitivity analyses, are displayed in Figure 4.

### 3.4. HIC Results

#### 3.4.1. Weight-for-Age

Fifty-one studies from HIC reported data on weight-for-age and included a total of 10,832 participants (5759 probiotics, 5074 control) [20,22,23,24,25,28,29,30,31,32,34,35,37,40,41,44,45,46,47,48,49,51,53,54,55,56,57,59,62,63,64,65,66,67,71,73,74,75,78,79,80,81,82,86,87,88,89,90,94,96,97]. The results provided moderate-certainty evidence that probiotics did not have a clinically meaningful effect on weight when compared to the control group (SMD: 0.01, 95% CI: −0.04–0.05, *p* = 0.78, I^2^ = 7%, Figure S16). The GRADE evidence was downgraded due to clinical heterogeneity in the use of probiotics in the included studies (Table 2). The funnel plot and Egger’s test showed no evidence of publication bias (*p* = 0.64, Figure S2). The sensitivity analysis without high-risk-of-bias studies showed similar results (SMD 0.00, 95% CI: −0.04–0.05, *p* = 0.88, I^2^ = 6%). A post hoc subgroup analysis based on type of strain did not show a strain or combination of strains that influenced any of the growth outcomes from studies from high-income countries (Table S7). All subgroup and sensitivity analyses did not show significantly different results for weight-for-age (Figure S32).

#### 3.4.2. Height-for-Age

Thirty-two studies from HIC reported data on height-for-age and included a total of 6118 participants(3350 probiotics, 2768 control) [20,22,25,28,30,31,37,40,41,44,45,49,53,54,55,56,63,66,67,71,73,75,78,79,80,86,87,88,90,94,96,97]. The results provided moderate evidence that probiotics did not have a clinically meaningful effect on height-for-age when compared to the control group (SMD: −0.01, 95% CI: −0.06–0.04, *p* = 0.71, I^2^ = 0%, Figure S17). The GRADE evidence was downgraded due to clinical heterogeneity of probiotics used in the included studies. The funnel plot and Egger’s test showed no evidence of publication bias (*p* = 0.87, Figure S4). All subgroup and sensitivity analyses did not show significantly different results for height-for-age (Figure S32).

#### 3.4.3. Other Outcomes

Probiotics did not show any significant effect on other primary and secondary outcomes in studies from HIC (forest plots in Figures S16–S25). The summary risk of bias for effect of probiotics for weight-for-age and other outcomes is shown in Figures S26–S31 of the supplementary document. A funnel plot for head circumference in HIC is found in Figure S5. The other analyses for HIC, including subgroup and sensitivity analyses, are displayed in Figure S32.

## 4. Discussion

This comprehensive systematic review evaluated the effects of probiotics on growth in children 0 to 59 months of age. Overall, there was no evidence that probiotics had a clinically meaningful effect on any of the growth outcomes in children from high-income countries. The data from low- and middle-income countries showed that there may be a small beneficial effect on weight and height gain; however, the certainty of evidence was low and moderate for these outcomes. There was no evidence that probiotics increased the risk of any of the adverse events including risk of sepsis from HIC and LMIC.

We used the GRADE approach to assess the overall certainty of evidence for the effect of probiotics on primary outcomes and selected secondary outcomes. The GRADE method of certainty assessment gives ratings of evidence for each outcome and considers factors such as type of study, risk of bias, inconsistency of results, indirectness of evidence, imprecision of the summary estimate, and publication bias [19]. All included studies were randomized trials, and we did not downgrade the certainty rating for study designs for any of the outcomes graded. Overall, there were a few studies that were at high risk of bias, and sensitivity analyses removing these studies did not substantively change the results, so we did not adjust the overall certainty of evidence rating for risk of bias. However, we did adjust the certainty rating due to clinical heterogeneity in the use of probiotics for all the outcomes graded. We conducted separate GRADE assessments for studies from high-income countries and those from low- and middle-income countries because we believe that environmental factors, diet, and gut microbiome might be different in these settings [2]. We therefore did not downgrade the certainty of evidence for indirectness for any of the graded outcomes. The number of studies that contributed data for an outcome varied among the outcomes. We downgraded the evidence for imprecision where the number of included studies was small and the confidence interval included a null effect.

Even though there was clinical heterogeneity in the use of probiotics, the pooled results were mostly homogenous around the null effect, especially from studies from high-income countries. Therefore, it can be concluded with reasonable confidence that probiotic supplementation in otherwise healthy children from high-income countries does not give any differential effect in terms of growth. The findings from trials conducted in low- and middle-income countries were mixed. The pooled results for the effects of probiotics on weight-for-age from low- and middle-income countries showed a small effect in favor of the intervention (SMD 0.26, 95% CI 0.11–0.42); however, there was significant statistical heterogeneity in the pooled data (I^2^ = 87%) and the positive effect can be explained by three studies [27,36,92] that had an effect SMD > 1. Removal of these studies with outlying findings yielded results that were similar to those from high-income countries (SMD 0.05, 95% CI −0.02, 0.12). We adjusted the GRADE evidence by lowering the certainty grade to ‘low’ for this outcome, which means that we have low confidence in this estimate and future research might change this estimate.

Were there any subgroups that might be affected differently? The data from high-income countries did not show any differential effects of probiotics for groups such as age < 6 months vs. 6–59 months, probiotics vs. synbiotics, or single vs. multiple strains and nutritional status (Figure S32). Thus, there does not seem to be an overall effect or any significant effect in subgroups from studies from high-income countries. The subgroup analyses from low- and middle-income countries are hard to interpret as the number of studies in each subgroup varied and some of the differences observed could be due to the small number of included studies. Nonetheless, there was evidence that single-strain probiotics may yield a more pronounced effect on weight-for-age compared to multiple-strain probiotics (Figure 4), although no particular single strain can be attributed to this result. This result was only present when pooling many studies that utilized a single-strain intervention. Future large studies will be required to further elaborate on and potentially replicate this finding. Indeed, more-targeted therapies could be more beneficial rather than using a single strain or combination of strains of probiotics [3].

This study is one of the largest systematic reviews conducted on the subject. We searched multiple databases and examined 11,158 titles and abstracts, and this included both published and ongoing studies. We did not apply any limitations of the literature search and did not exclude studies at the title/abstract screenings stages if they did not report outcomes in the abstract. This might be the reason that we were able to include many more studies compared to the last review published in 2014 [12]. We specified our analyses a priori and registered our protocol on a publicly available website before the review started. The major post hoc decision was to include studies from high-income countries as we understand that probiotics are commonly used to promote healthy gut microbiome in high-income countries, and it is important to review the available literature for their effect in promotion of healthy growth in children. We therefore conducted separate meta-analyses and certainty ratings for high-income countries and low- and middle-income countries. The limitations of the data presented in this study were that there was significant clinical heterogeneity in the use of probiotics with respect to type, duration, and combination. It is debatable if a meta-analysis should have been performed in the presence of such clinical heterogeneity. We believe that a meta-analysis was appropriate here as it served the purpose of assessing the overall magnitude and direction of effect from included studies. We adjusted the GRADE ratings for each outcome for clinical heterogeneity and conducted a post hoc subgroup analysis examining each strain or combination of strains and found no differential effect for any single strain or combination of strains for any of the growth outcomes assessed. Another potential limitation is related to type of growth outcomes included. Although we included a range of outcomes for growth, most of the outcomes were continuous and we did not include dichotomous outcomes such as undernutrition and stunting. We posit that if probiotics had any significant effect on prevention of stunting and undernutrition then that pattern would be mirrored in their effects on average height and weight gain.

There is a growing appreciation for the role of the gut microbiota, and accumulating evidence suggests an association between immaturity of the microbial community and undernutrition [4,99,100]. Although our understanding of the dynamic interplay between undernutrition and the microbiota is improving, empirical work examining therapeutic interventions to ameliorate microbiota dysbiosis remains limited. Methods that harness the microbiota through rationally designed microbial consortia, or manipulate the community through microbiota-directed therapeutic foods, show promise for treatment [3,4]. Our observation that children in low- and middle-income countries might benefit from probiotic supplementation is consistent with recent findings regarding the beneficial effects of a microbiota-targeted food intervention in Bangladesh [3]. There is a need for large-scale clinical trials that address the multifaceted role of the microbiota in childhood nutrition.

## 5. Conclusions

Probiotic supplementation does not seem to have a clinically meaningful effect on growth for apparently healthy children in high-income countries. However, there might be a small effect on weight and height in apparently healthy children from low- and middle-income countries. Future large-scale clinical trials are needed that assess the targeted therapies to prevent the gut dysbiosis associated with childhood undernutrition in low- and middle-income countries.

## Figures and Tables

**Table 1:** GRADE evidence profile showing results of GRADE analysis of overall certainty of evidence for effect of probiotics in children 0–59 months of age in low- and middle-income countries.

Certainty Assessment							№ of Patients		Effect		Certainty
No of Studies	Study Design	Risk of Bias	Inconsistency	Indirectness	Imprecision	Other Considerations	Probiotics	Control	Relative(95% CI)	Absolute (95% CI)	
Weight-for-age											
21	RCT	not serious ^a^	very serious ^b^	not serious ^c^	not serious ^d^	none	4323	4094	-	SMD 0.26 higher (0.11 higher to 0.42 higher)	⨁⨁◯◯ LOW
Height-for-age											
12	RCT	not serious ^e^	serious ^f^	not serious ^c^	not serious	none	1347	1214	-	SMD 0.16 higher (0.06 higher to 0.25 higher)	⨁⨁⨁◯ MODERATE
Head Circumference											
9	RCT	not serious ^e^	serious ^g^	not serious ^c^	serious ^h^	none	819	816	-	SMD 0.05 higher (0.11 lower to 0.21 higher)	⨁⨁◯◯ LOW
BMI											
2	RCT	not serious ^e^	serious ^i^	not serious	serious ^j^	none	171	168	-	SMD 0.05 lower (0.26 lower to 0.17 higher)	⨁⨁◯◯ LOW
Sepsis											
9	RCT	not serious ^k^	serious ^l^	not serious	not serious	none	312/3026 (10.3%)	441/3024 (14.6%)	RR 0.74 (0.64 to 0.87)	38 fewer per 1000 (from 53 fewer to 19 fewer)	⨁⨁⨁◯ MODERATE

Footnotes: ^a.^ Even though three of the included studies were at high risk of bias for this outcome, a sensitivity analysis by excluding these studies did not change the magnitude, direction, or statistical significance of the summary estimate. ^b.^ The I^2^ was 87%. Inspection of the forest plot showed the effect of probiotics varied in magnitude. We also downgraded for heterogeneity in the use of probiotics used in the included studies. ^c.^ All the studies were conducted in low-r and middle-income countries ^d.^ Overall sample size from all the included studies in the meta-analysis was more than 8000. The CI did not include 0. ^e.^ None of the included studies in this analysis were at high risk of bias. ^f.^ Even though the statistical heterogeneity was only 25%, we downgraded for clinical heterogeneity in the use of probiotics in the included studies. ^g.^ The I^2^ was 57% ^h.^ The overall sample size was less than 2000 and the confidence interval of the summary estimate included 0. ^i.^ Even though the statistical heterogeneity was only 0%, we downgraded for clinical heterogeneity in the use of probiotics in the included studies. ^j.^ The overall sample size of the included studies was less than 400 and the confidence interval of the summary estimate was wide and included 0. ^k.^ Even though one of the included studies was at high risk of bias for this outcome, a sensitivity analysis by excluding this study did not change the magnitude, direction, or statistical significance of the summary estimate. ^l.^ Even though the statistical heterogeneity was only 20%, we downgraded for clinical heterogeneity in the use of probiotics in the included studies. Abbreviations: CI: confidence interval; SMD: standardized mean difference; RR: risk ratio, BMI: body mass index RCT: randomized controlled trial.

**Table 2:** Summary-of-Findings table showing results of GRADE analysis of overall evidence for effect of probiotics in children 0–59 months of age in high-income countries.

Certainty Assessment							№ of Patients		Effect		Certainty
No of Studies	Study Design	Risk of Bias	Inconsistency	Indirectness	Imprecision	Other Considerations	Probiotics	Control	Relative (95% CI)	Absolute (95% CI)	
Weight-for-age											
51	RCT	not serious ^a^	serious ^b^	not serious ^c^	not serious ^d^	none	5759	5073	-	SMD 0.01 higher (0.04 lower to 0.05 higher)	⨁⨁⨁◯ MODERATE
Height-for-age											
32	RCT	not serious ^a^	serious ^e^	not serious ^c^	not serious ^d^	none	3350	2768	-	SMD 0.01 lower (0.06 lower to 0.04 higher)	⨁⨁⨁◯ MODERATE
Head Circumference											
28	RCT	not serious ^f^	serious ^g^	not serious ^c^	not serious ^d^	none	2655	2117	-	SMD 0.04 lower (0.2 lower to 0.11 higher)	⨁⨁⨁◯ MODERATE
BMI											
5	RCT	not serious	serious ^e^	not serious ^c^	serious ^h^	none	415	305	-	SMD 0.09 higher (0.06 lower to 0.25 higher)	⨁⨁◯◯ LOW
Sepsis											
12	RCT	not serious ^i^	serious ^j^	not serious ^c^	serious ^k^	none	275/1778 (15.5%)	278/1749 (15.9%)	RR 1.03 (0.84 to 1.26)	5 more per 1000 (from 25 fewer to 41 more)	⨁⨁◯◯ LOW

Explanations: ^a.^ Even though four of the included studies were at high risk of bias for this outcome, the sensitivity analysis by the exclusion of these studies did not change the magnitude, direction, or statistical significance of the analysis. ^b.^ Even though the statistical heterogeneity was very low with I^2^ values of 7%, we downgraded for clinical heterogeneity in the type of probiotics used in the included studies. ^c.^ All the studies were conducted in high-income countries. ^d.^ The confidence interval of the effect size includes 0, and we think the overall effect is about 0. The confidence intervals are narrow enough that we do not think that the summary size is imprecise. ^e.^ Even though the statistical heterogeneity was homogenous with I^2^ values of 0%, we downgraded for clinical heterogeneity in the type of probiotics used in the included studies. ^f.^ Even though three of the included studies were at high risk of bias for this outcome, the sensitivity analysis by the exclusion of these studies did not change the magnitude, direction, or statistical significance of the analysis. ^g.^ The I^2^ was 85% ^h.^ The total sample size of all the studies included in the meta-analysis was less than 1000. The CIs were wide ^i.^ Even though one of the included studies was at high risk of bias for this outcome, the sensitivity analysis by the exclusion of this study did not change the magnitude, direction, or statistical significance of the analysis. ^j.^ Even though the statistical heterogeneity was low with I^2^ values of 26%, we downgraded for clinical heterogeneity in the type of probiotics used in the included studies. ^k.^ The total sample size of all the studies included in the meta-analysis was less than 1000. The CIs were wide, and increased risk cannot be excluded. Abbreviations: CI: confidence interval; SMD: standardized mean difference; RR: risk ratio, BMI: body mass index RCT: randomized controlled trial.

## Data Availability

We will share data-extraction sheets. Furthermore, we are willing to share our risk-of-bias assessment and meta-analysis RevMan file on request via email.

## Supplementary Materials

The following are available online at https://www.mdpi.com/article/10.3390/nu14010083/s1, Text S1: Search strategies for databases through November 6, 2020; Text S2: Extended data-input details; Text S3: Results: Study Locations; Table S1: Excluded studies and reasons for exclusion; Table S2: Participant characteristics in the included studies from low- and middle-income countries; Table S3: Participant characteristics in the included studies from high-income countries; Table S4: Treatment and comparison characteristics in the included studies from low- and middle-income countries; Table S5: Treatment and comparison characteristics in the included studies from high-income countries; Table S6: Pooled analysis based on probiotic strains: Low- and middle-income countries; Table S7: Pooled analysis based on probiotic strains: High-income countries; Figure S1: Funnel Plot: Effect of probiotics on growth: Low- and middle-income countries: Weight; Figure S2: Funnel Plot: Effect of probiotics on growth: High-income countries: Weight; Figure S3: Funnel Plot: Effect of probiotics on growth: Low- and middle-income countries: Head Circumference; Figure S4: Funnel Plot: Effect of probiotics on growth: High-income countries: Height; Figure S5: Funnel Plot: Effect of probiotics on growth: High-income countries: Head circumference; Figure S6: Risk of bias in the included studies from low- and middle-income countries for effect of probiotics on the outcome of weight-for-age; Figure S7: Risk of bias in the included studies from low- and middle-income countries for the outcome of height-for-age; Figure S8: Risk of bias in the included studies from low- and middle-income countries for the outcome of head circumference; Figure S9: Risk of bias in the included studies from low- and middle-income countries for the outcome of sepsis; Figure S10: Forest Plot: Effect of use of probiotics on head circumference in children 0–59 months of age from low- and middle-income countries; Figure S11: Forest Plot: Effect of use of probiotics on BMI in children 0–59 months of age from low- and middle-income countries; Figure S12: Forest Plot: Effect of use of probiotics on weight for height in children 0–59 months of age from low- and middle-income countries; Figure S13: Forest Plot: Effect of use of probiotics on adverse events: Sepsis, in children 0–59 months of age from low- and middle-income countries; Figure S14: Forest Plot: Effect of use of probiotics on adverse events: Vomiting, in children 0–59 months of age from low- and middle-income countries; Figure S15: Forest Plot: Effect of use of probiotics on adverse events: Diarrhea, in children 0–59 months of age from low- and middle-income countries; Figure S16: Forest Plot Effect of use of probiotics on weight-for-age in children 0–59 months of age from high-income countries; Figure S17: Forest Plot: Effect of use of probiotics on height-for-age in children 0–59 months of age from high-income countries; Figure S18: Forest Plot: Effect of use of probiotics on weight-for-length in children 0–59 months of age from high-income countries; Figure S19: Forest Plot: Effect of use of probiotics on head circumference for age in children 0–59 months of age from high-income countries; Figure S20: Forest Plot: Effect of use of probiotics on BMI in children 0–59 months of age from high-income countries; Figure S21: Forest Plot: Effect of use of probiotics on adverse events: Sepsis, in children 0–59 months of age from high-income countries; Figure S22: Forest Plot: Effect of use of probiotics on adverse events: Vomiting, in children 0–59 months of age from high-income countries; Figure S23: Forest Plot: Effect of use of probiotics on adverse events: Diarrhea, in children 0–59 months of age from high-income countries; Figure S24: Forest Plot: Effect of use of probiotics on adverse events: Abdominal pain, in children 0–59 months of age from high-income countries; Figure S25: Forest Plot: Effect of use of probiotics on adverse events: Flatulence, in children 0–59 months of age from high-income countries; Figure S26: Risk of bias in the included studies from high-income countries for effect of probiotics on the outcome of weight-for-age; Figure S27: Risk of bias in the included studies from high-income countries for effect of probiotics on the outcome of height-for-age; Figure S28: Risk of bias in the included studies from high-income countries for effect of probiotics on the outcome of weight-for-length; Figure S29: Risk of bias in the included studies from high-income countries for effect of probiotics on the outcome of head circumference; Figure S30: Risk of bias in the included studies from high-income countries for effect of probiotics on the outcome of BMI; Figure S31: Risk of bias in the included studies from high-income countries for effect of probiotics on the outcome of sepsis; Figure S32: Subgroup and Sensitivity analyses in high-income countries.

## References

[1] Rackaityte E., Lynch S.V. (2020). The human microbiome in the 21(st) century. Nat. Commun..

[2] Blanton L.V., Charbonneau M.R., Salih T., Barratt M.J., Venkatesh S., Ilkaveya O., Subramanian S., Manary M.J., Trehan I., Jorgensen J.M. (2016). Gut bacteria that prevent growth impairments transmitted by microbiota from malnourished children. Science.

[3] Chen R.Y., Mostafa I., Hibberd M.C., Das S., Mahfuz M., Naila N.N., Islam M.M., Huq S., Alam M.A., Zaman M.U. (2021). A Microbiota-Directed Food Intervention for Undernourished Children. N. Engl. J. Med..

[4] Gehrig J.L., Venkatesh S., Chang H.W., Hibberd M.C., Kung V.L., Cheng J., Chen R.Y., Subramanian S., Cowardin C.A., Meier M.F. (2019). Effects of microbiota-directed foods in gnotobiotic animals and undernourished children. Science.

[5] Robertson R.C., Manges A.R., Finlay B.B., Prendergast A.J. (2019). The Human Microbiome and Child Growth—First 1000 Days and Beyond. Trends Microbiol..

[6] Probiotics Market Size, Share & Trends Analysis Report by Product (Food & Beverages, Dietary Supplements), By Ingredient (Bacteria, Yeast), By End Use, By Distribution Channel, And Segment Forecasts, 2021–2028. https://www.grandviewresearch.com.

[7] Depoorter L., Vandenplas Y. (2021). Probiotics in Pediatrics. A Review and Practical Guide. Nutrients.

[8] Hill C., Guarner F., Reid G., Gibson G.R., Merenstein D.J., Pot B., Morelli L., Canani R.B., Flint H.J., Salminen S. (2014). The International Scientific Association for Probiotics and Prebiotics consensus statement on the scope and appropriate use of the term probiotic. Nat. Rev. Gastroenterol. Hepatol..

[9] Roberfroid M.B. (2001). Prebiotics: Preferential substrates for specific germs?. Am. J. Clin. Nutr..

[10] Collins M.D., Gibson G.R. (1999). Probiotics, prebiotics, and synbiotics: Approaches for modulating the microbial ecology of the gut. Am. J. Clin. Nutr..

[11] Million M., Angelakis E., Paul M., Armougom F., Leibovici L., Raoult D. (2012). Comparative meta-analysis of the effect of Lactobacillus species on weight gain in humans and animals. Microb. Pathog..

[12] Onubi O.J., Poobalan A.S., Dineen B., Marais D., McNeill G. (2015). Effects of probiotics on child growth: A systematic review. J. Health Popul. Nutr..

[13] Steenhout P.G., Rochat F., Hager C. (2009). The effect of Bifidobacterium lactis on the growth of infants: A pooled analysis of randomized controlled studies. Ann. Nutr. Metab..

[14] Covidence systematic review software, Veritas Health Innovation, Melbourne, Australia. www.covidence.org.

[15] Serce O., Gursoy T., Karatekin G., Ovali F. (2013). Effects of prebiotic and probiotic combination on necrotizing enterocolitis and sepsis prophylaxis in very low birth weight infants. J. Perinat. Med..

[16] StataCorp (2017). Stata Statistical Software: Release 15.

[17] Higgins J.P.T., Green S., Cochrane Handbook for Systematic Reviews of Interventions Version 5.1.0 [updated March 2011] (2011). The Cochrane Collaboration. www.handbook.cochrane.org.

[18] Sterne J.A.C., Savovic J., Page M.J., Elbers R.G., Blencowe N.S., Boutron I., Cates C.J., Cheng H.Y., Corbett M.S., Eldridge S.M. (2019). RoB 2: A revised tool for assessing risk of bias in randomised trials. BMJ.

[19] Guyatt G., Oxman A.D., Akl E.A., Kunz R., Vist G., Brozek J., Norris S., Falck-Ytter Y., Glasziou P., DeBeer H. (2011). GRADE guidelines: 1. Introduction-GRADE evidence profiles and summary of findings tables. J. Clin. Epidemiol..

[20] Abrahamse-Berkeveld M., Alles M., Franke-Beckmann E., Helm K., Knecht R., Kollges R., Sandner B., Knol J., Ben Amor K., Bufe A. (2016). Infant formula containing galacto-and fructo-oligosaccharides and Bifidobacterium breve M-16V supports adequate growth and tolerance in healthy infants in a randomised, controlled, double-blind, prospective, multicentre study. J. Nutr. Sci..

[21] Agustina R., Bovee-Oudenhoven I.M., Lukito W., Fahmida U., van de Rest O., Zimmermann M.B., Firmansyah A., Wulanti R., Albers R., van den Heuvel E.G. (2013). Probiotics Lactobacillus reuteri DSM 17938 and Lactobacillus casei CRL 431 modestly increase growth, but not iron and zinc status, among Indonesian children aged 1-6 years. J. Nutr..

[22] Ahrens B., Hellmuth C., Haiden N., Olbertz D., Hamelmann E., Vusurovic M., Fleddermann M., Roehle R., Knoll A., Koletzko B. (2018). Hydrolyzed Formula with Reduced Protein Content Supports Adequate Growth: A Randomized Controlled Noninferiority Trial. J. Pediatr. Gastroenterol. Nutr..

[23] Al-Hosni M., Duenas M., Hawk M., Stewart L.A., Borghese R.A., Cahoon M., Atwood L., Howard D., Ferrelli K., Soll R. (2012). Probiotics-supplemented feeding in extremely low-birth-weight infants. J. Perinatol..

[24] Allen S.J., Jordan S., Storey M., Thornton C.A., Gravenor M., Garaiova I., Plummer S.F., Wang D., Morgan G. (2010). Dietary supplementation with lactobacilli and bifidobacteria is well tolerated and not associated with adverse events during late pregnancy and early infancy. J. Nutr..

[25] Aloisio I., Prodam F., Giglione E., Bozzi Cionci N., Solito A., Bellone S., Baffoni L., Mogna L., Pane M., Bona G. (2018). Three-Month Feeding Integration with Bifidobacterium Strains Prevents Gastrointestinal Symptoms in Healthy Newborns. Front. Nutr..

[26] Amini E., Dalili H., Niknafs N., Shariat M., Nakhostin M., Jedari-Attari S. (2017). The effect of probiotics in prevention of necrotising enterocolitis in preterm neonates in comparison with control group. Iran. J. Pediatrics.

[27] Batac M.C.R., Guno M.J.V., Caparas-de Castro C., Gutierrez-Santos K., Tondoc A. (2005). Effects of a probiotic formula on measles, mumps and rubella IgG production and on anthropometric measurements of infants aged 11-15 months in a tertiary hospital. St. Tomas J. Med..

[28] Bazanella M., Maier T.V., Clavel T., Lagkouvardos I., Lucio M., Maldonado-Gòmez M.X., Autran C., Walter J., Bode L., Schmitt-Kopplin P. (2017). Randomized controlled trial on the impact of early-life intervention with bifidobacteria on the healthy infant fecal microbiota and metabolome. Am. J. Clin. Nutr..

[29] Bin-Nun A., Bromiker R., Wilschanski M., Kaplan M., Rudensky B., Caplan M., Hammerman C. (2005). Oral probiotics prevent necrotizing enterocolitis in very low birth weight neonates. J. Pediatr..

[30] Cekola P.L., Czerkies L.A., Storm H.M., Wang M.H., Roberts J., Saavedra J.M. (2015). Growth and Tolerance of Term Infants Fed Formula With Probiotic Lactobacillus re.e.euteri. Clin. Pediatr..

[31] Chouraqui J.P., Grathwohl D., Labaune J.M., Hascoet J.M., de Montgolfier I., Leclaire M., Giarre M., Steenhout P. (2008). Assessment of the safety, tolerance, and protective effect against diarrhea of infant formulas containing mixtures of probiotics or probiotics and prebiotics in a randomized controlled trial. Am. J. Clin. Nutr..

[32] Chrzanowska-Liszewska D., Seliga-Siwecka J., Kornacka M.K. (2012). The effect of Lactobacillus rhamnosus GG supplemented enteral feeding on the microbiotic flora of preterm infants-double blinded randomized control trial. Early Hum. Dev..

[33] Cooper P., Bolton K.D., Velaphi S., de Groot N., Emady-Azar S., Pecquet S., Steenhout P. (2016). Early Benefits of a Starter Formula Enriched in Prebiotics and Probiotics on the Gut Microbiota of Healthy Infants Born to HIV+ Mothers: A Randomized Double-Blind Controlled Trial. Clin. Med. Insights Pediatr..

[34] Costalos C., Skouteri V., Gounaris A., Sevastiadou S., Triandafilidou A., Ekonomidou C., Kontaxaki F., Petrochilou V. (2003). Enteral feeding of premature infants with Saccharomyces boulardii. Early Hum. Dev..

[35] Costeloe K., Hardy P., Juszczak E., Wilks M., Millar M.R. (2016). Bifidobacterium breve BBG-001 in very preterm infants: A randomised controlled phase 3 trial. Lancet.

[36] Cui X., Shi Y., Gao S., Xue X., Fu J. (2019). Efffects of Lactobacillus reuteri DSM 17938 in preterm infants: A double-blinded randomized controlled study. Ital. J. Pediatr..

[37] Dekker J.W., Wickens K., Black P.N., Stanley T.V., Mitchell E.A., Fitzharris P., Tannock G.W., Purdie G., Crane J. (2009). Safety aspects of probiotic bacterial strains Lactobacillus rhamnosus HN001 and Bifidobacterium animalis subsp. lactis HN019 in human infants aged 0–2 years. Int. Dairy J..

[38] Demirel G., Erdeve O., Celik I.H., Dilmen U. (2013). Saccharomyces boulardii for prevention of necrotizing enterocolitis in preterm infants: A randomized, controlled study. Acta Paediatr.

[39] Dilli D., Aydin B., Fettah N.D., Ozyazici E., Beken S., Zenciroglu A., Okumus N., Ozyurt B.M., Ipek M.S., Akdag A. (2015). The propre-save study: Effects of probiotics and prebiotics alone or combined on necrotizing enterocolitis in very low birth weight infants. J. Pediatr.

[40] Escribano J., Ferre N., Gispert-Llaurado M., Luque V., Rubio-Torrents C., Zaragoza-Jordana M., Polanco I., Codoner F.M., Chenoll E., Morera M. (2018). Bifidobacterium longum subsp infantis CECT7210-supplemented formula reduces diarrhea in healthy infants: A randomized controlled trial. Pediatr. Res..

[41] Gil-Campos M., Lopez M.A., Rodriguez-Benitez M.V., Romero J., Roncero I., Linares M.D., Maldonado J., Lopez-Huertas E., Berwind R., Ritzenthaler K.L. (2012). Lactobacillus fermentum CECT 5716 is safe and well tolerated in infants of 1-6 months of age: A randomized controlled trial. Pharmacol. Res..

[42] Guney Varal I., Koksal N., Ozkan H., Bagci O., Dogan P. (2018). Potential use of multi-strain synbiotics for improving postnatal head circumference. Pak. J. Med. Sci..

[43] Harvey B.M., Langford J.E., Harthoorn L.F., Gillman S.A., Green T.D., Schwartz R.H., Burks A.W. (2014). Effects on growth and tolerance and hypoallergenicity of an amino acid-based formula with synbiotics. Pediatr. Res..

[44] Hays S., Jacquot A., Gauthier H., Kempf C., Beissel A., Pidoux O., Jumas-Bilak E., Decullier E., Lachambre E., Beck L. (2015). Probiotics and growth in preterm infants: A randomized controlled trial, PREMAPRO study. Clin. Nutr..

[45] Hojsak I., Snovak N., Abdovic S., Szajewska H., Misak Z., Kolacek S. (2010). Lactobacillus GG in the prevention of gastrointestinal and respiratory tract infections in children who attend day care centers: A randomized, double-blind, placebo-controlled trial. Clin. Nutr..

[46] Indrio F., Riezzo G., Raimondi F., Bisceglia M., Cavallo L., Francavilla R. (2008). The effects of probiotics on feeding tolerance, bowel habits, and gastrointestinal motility in preterm newborns. J. Pediatr..

[47] Jacobs S.E., Tobin J.M., Opie G.F., Donath S., Tabrizi S.N., Pirotta M., Morley C.J., Garland S.M., ProPrems Study G. (2013). Probiotic effects on late-onset sepsis in v.very preterm infants: A randomized controlled trial. Pediatrics.

[48] Kankaanpaa P.E., Yang B., Kallio H.P., Isolauri E., Salminen S.J. (2002). Influence of probiotic supplemented infant formula on composition of plasma lipids in atopic infants. J. Nutr. Biochem..

[49] Kukkonen K., Savilahti E., Haahtela T., Juntunen-Backman K., Korpela R., Poussa T., Tuure T., Kuitunen M. (2008). Long-term safety and impact on infection rates of postnatal probiotic and prebiotic (synbiotic) treatment: Randomized, double-blind, placebo-controlled trial. Pediatrics.

[50] Li X., Peng Y., Li Z., Christensen B., Heckmann A.B., Stenlund H., Lönnerdal B., Hernell O. (2019). Feeding Infants Formula with Probiotics or Milk Fat Globule Membrane: A Double-Blind, Randomized Controlled Trial. Front. Pediatrics.

[51] Lin H.C., Hsu C.H., Chen H.L., Chung M.Y., Hsu J.F., Lien R.I., Tsao L.Y., Chen C.H., Su B.H. (2008). Oral probiotics prevent necrotizing enterocolitis in very low birth weight preterm infants: A multicenter, randomized, controlled trial. Pediatrics.

[52] Luoto R., Kalliomaki M., Laitinen K., Isolauri E. (2010). The impact of perinatal probiotic intervention on the development of overweight and obesity: Follow-up study from birth to 10 years. Int. J. Obes..

[53] Maldonado J., Canabate F., Sempere L., Vela F., Sanchez A.R., Narbona E., Lopez-Huertas E., Geerlings A., Valero A.D., Olivares M. (2012). Human milk probiotic Lactobacillus fermentum CECT5716 reduces the incidence of gastrointestinal and upper respiratory tract infections in infants. J. Pediatr. Gastroenterol. Nutr..

[54] Maldonado J., Gil-Campos M., Maldonado-Lobon J.A., Benavides M.R., Flores-Rojas K., Jaldo R., Jimenez Del Barco I., Bolivar V., Valero A.D., Prados E. (2019). Evaluation of the safety, tolerance and efficacy of 1-year consumption of infant formula supplemented with Lactobacillus fermentum CECT5716 Lc40 or Bifidobacterium breve CECT7263: A randomized controlled trial. BMC Pediatr..

[55] Maldonado J., Lara-Villoslada F., Sierra S., Sempere L., Gomez M., Rodriguez J.M., Boza J., Xaus J., Olivares M. (2010). Safety and tolerance of the human milk probiotic strain Lactobacillus salivarius CECT5713 in 6-month-old children. Nutrition.

[56] Manzano S., De Andres J., Castro I., Rodriguez J.M., Jimenez E., Espinosa-Martos I. (2017). Safety and tolerance of three probiotic strains in healthy infants: A multi-centre randomized, double-blind, placebo-controlled trial. Benef. Microbes.

[57] Millar M.R., Bacon C., Smith S.L., Walker V., Hall M.A. (1993). Enteral feeding of premature infants with Lactobacillus GG. Arch. Dis. Child..

[58] Mitra M., Adarsh E., Narang A., Agrawal R., Vaidya U., Ganguly S. (2014). Safety and tolerance of infant formulas containing probiotics in India: A multicenter randomized controlled trial. J. Matern.-Fetal Neonatal Med..

[59] Mohan R., Koebnick C., Schildt J., Mueller M., Radke M., Blaut M. (2008). Effects of Bifidobacterium lactis Bb12 supplementation on body weight, fecal pH, acetate, lactate, calprotectin, and IgA in preterm infants. Pediatr. Res..

[60] Panigrahi P., Parida S., Nanda N.C., Satpathy R., Pradhan L., Chandel D.S., Baccaglini L., Mohapatra A., Mohapatra S.S., Misra P.R. (2017). A randomized synbiotic trial to prevent sepsis among infants in rural India. Nature.

[61] Panigrahi P., Parida S., Pradhan L., Mohapatra S.S., Misra P.R., Johnson J.A., Chaudhry R., Taylor S., Hansen N.I., Gewolb I.H. (2008). Long-term colonization of a Lactobacillus plantarum synbiotic preparation in the neonatal gut. J. Pediatr. Gastroenterol. Nutr..

[62] Papagaroufalis K., Fotiou A., Egli D., Tran L.A., Steenhout P. (2014). A Randomized Double Blind Controlled Safety Trial Evaluating d-Lactic Acid Production in Healthy Infants Fed a Lactobacillus reuteri-containing Formula. Nutr. Metab. Insights.

[63] Puccio G., Cajozzo C., Meli F., Rochat F., Grathwohl D., Steenhout P. (2007). Clinical evaluation of a new starter formula for infants containing live Bifidobacterium longum.m.m BL999 and prebiotics. Nutrition.

[64] Radke M., Picaud J.C., Loui A., Cambonie G., Faas D., Lafeber H.N., de Groot N., Pecquet S.S., Steenhout P.G., Hascoet J.M. (2017). Starter formula enriched in prebiotics and probiotics ensures normal growth of infants and promotes gut health: A randomized clinical trial. Pediatr. Res..

[65] Rouge C., Piloquet H., Butel M.J., Berger B., Rochat F., Ferraris L., Des Robert C., Legrand A., de la Cochetiere M.F., N’Guyen J.M. (2009). Oral supplementation with probiotics in very-low-birth-weight preterm infants: A randomized, double-blind, placebo-controlled trial. Am. J. Clin. Nutr..

[66] Roze J.C., Barbarot S., Butel M.J., Kapel N., Waligora-Dupriet A.J., De Montgolfier I., Leblanc M., Godon N., Soulaines P., Darmaun D. (2012). An alpha-lactalbumin-enriched and symbiotic-supplemented v. a standard infant formula: A multicentre, double-blind, randomised trial. Br. J. Nutr..

[67] Saavedra J.M., Abi-Hanna A., Moore N., Yolken R.H. (2004). Long-term consumption of infant formulas containing live probiotic bacteria: Tolerance and safety. Am. J. Clin. Nutr..

[68] Saengtawesin V., Tangpolkaiwalsak R., Kanjanapattankul W. (2014). Effect of oral probiotics supplementation in the prevention of necrotizing enterocolitis among very low birth weight preterm infants. J. Med. Assoc. Thail..

[69] Sari F.N., Dizdar E.A., Oguz S., Erdeve O., Uras N., Dilmen U. (2010). Oral probiotics: Lactobacillus sporogenes in prevention of necrotizing enterocolitis in very low birth weight infants: A randomized, controlled trial. Early Hum. Dev..

[70] Sazawal S., Dhingra U., Hiremath G., Sarkar A., Dhingra P., Dutta A., Menon V.P., Black R.E. (2010). Effects of Bifidobacterium lactis HN019 and prebiotic oligosaccharide added to milk on iron status, anemia, and growth among children 1 to 4 years old. J. Pediatr. Gastroenterol. Nutr..

[71] Scalabrin D.M., Johnston W.H., Hoffman D.R., P’Pool V.L., Harris C.L., Mitmesser S.H. (2009). Growth and tolerance of healthy term infants receiving hydrolyzed infant formulas supplemented with Lactobacillus rhamnosus GG: Randomized, double-blind, controlled trial. Clin. Pediatr..

[72] Serce O., Benzer D., Gursoy T., Karatekin G., Ovali F. (2013). Efficacy of Saccharomyces boulardii on necrotizing enterocolitis or sepsis in very low birth weight infants: A randomised controlled trial. Early Hum. Dev..

[73] Simeoni U., Berger B., Junick J., Blaut M., Pecquet S., Rezzonico E., Grathwohl D., Sprenger N., Brussow H., Study T. (2016). Gut microbiota analysis reveals a ma.arked shift to bifidobacteria by a starter infant formula containing a synbiotic of bovine milk-derived oligosaccharides and Bifidobacterium animalis subsp. lactis CNCM I-3446. Environ. Microbiol..

[74] Smilowitz J.T., Moya J., Breck M.A., Cook C., Fineberg A., Angkustsiri K., Underwood M.A. (2017). Safety and tolerability of Bifidobacterium longum subspecies infantis EVC001 supplementation in healthy term breastfed infants: A phase I clinical trial. BMC Pediatr..

[75] Stratiki Z., Costalos C., Sevastiadou S., Kastanidou O., Skouroliakou M., Giakoumatou A., Petrohilou V. (2007). The effect of a bifidobacter supplemented bovine milk on intestinal permeability of preterm infants. Early Hum. Dev..

[76] Sur D., Manna B., Niyogi S.K., Ramamurthy T., Palit A., Nomoto K., Takahashi T., Shima T., Tsuji H., Kurakawa T. (2011). Role of probiotic in preventing acute diarrhoea in children: A community-based, randomized, double-blind placebo-controlled field trial in an urban slum. Epidemiol. Infect..

[77] Surono I.S., Koestomo F.P., Novitasari N., Zakaria F.R., Yulianasari, Koesnandar (2011). Novel probiotic Enterococcus faecium IS-27526 supplementation increased total salivary sIgA level and bodyweight of pre-school children: A pilot study. Anaerobe.

[78] Szajewska H., Ruszczynski M., Szymanski H., Sadowska-Krawczenko I., Piwowarczyk A., Rasmussen P.B., Kristensen M.B., West C.E., Hernell O. (2017). Effects of infant formula supplemented with prebiotics compared with synbiotics on growth up to the age of 12 mo: A randomized controlled trial. Pediatr. Res..

[79] Taylor A.L., Dunstan J.A., Prescott S.L. (2007). Probiotic supplementation for the first 6 months of life fails to reduce the risk of atopic dermatitis and increases the risk of allergen sensitization in high-risk children: A randomized controlled trial. J. Allergy Clin. Immunol..

[80] Thibault H., Aubert-Jacquin C., Goulet O. (2004). Effects of long-term consumption of a fermented infant formula (with Bifidobacterium breve c50 and Streptococcus thermophilus 065) on acute diarrhea in healthy infants. J. Pediatr. Gastroenterol. Nutr..

[81] Totsu S., Yamasaki C., Terahara M., Uchiyama A., Kusuda S. (2014). Bifidobacterium and enteral feeding in preterm infants: Cluster-randomized trial. Pediatr. Int..

[82] Underwood M.A., Salzman N.H., Bennett S.H., Barman M., Mills D.A., Marcobal A., Tancredi D.J., Bevins C.L., Sherman M.P. (2009). A randomized placebo-controlled comparison of 2 prebiotic/probiotic combinations in preterm infants: Impact on weight gain, intestinal microbiota, and fecal short-chain fatty acids. J. Pediatr. Gastroenterol. Nutr..

[83] Urban M.F., Bolton K.D., Mokhachane M., Mphahlele R.M., Bomela H.N., Monaheng L., Beckh-Arnold E., Cooper P.A. (2008). Growth of infants born to HIV-infected women when fed a biologically acidified starter formula with and without probiotics. South Afr. J. Clin. Nutr..

[84] Van Niekerk E., Kirsten G.F., Nel D.G., Blaauw R. (2014). Probiotics, feeding tolerance, and growth: A comparison between HIV-exposed and unexposed very low birth weight infants. Nutrition.

[85] Velaphi S.C., Cooper P.A., Bolton K.D., Mokhachane M., Mphahlele R.M., Beckh-Arnold E., Monaheng L., Haschke-Becher E. (2008). Growth and metabolism of infants born to women infected with human immunodeficiency virus and fed acidified whey-adapted starter formulas. Nutrition.

[86] Vendt N., Grunberg H., Tuure T., Malminiemi O., Wuolijoki E., Tillmann V., Sepp E., Korpela R. (2006). Growth during the first 6 months of life in infants using formula enriched with Lactobacillus rhamnosus GG: Double-blind, randomized trial. J. Hum. Nutr. Diet..

[87] Vlieger A.M., Robroch A., van Buuren S., Kiers J., Rijkers G., Benninga M.A., te Biesebeke R. (2009). Tolerance and safety of Lactobacillus paracasei ssp. paracasei in combination with Bifidobacterium animalis ssp. lactis in a prebiotic-containing infant formula: A randomised controlled trial. Br. J. Nutr..

[88] Weizman Z., Alsheikh A. (2006). Safety and tolerance of a probiotic formula in early infancy comparing two probiotic agents: A pilot study. J. Am. Coll Nutr..

[89] Weizman Z., Asli G., Alsheikh A. (2005). Effect of a probiotic infant formula on infections in child care centers: Comparison of two probiotic agents. Pediatrics.

[90] Wejryd E., Marchini G., Frimmel V., Jonsson B., Abrahamsson T. (2019). Probiotics promoted head growth in extremely low birthweight infants in a double-blind placebo-controlled trial. Acta Paediatr..

[91] Xiao L., Gong C., Ding Y., Ding G., Xu X., Deng C., Ze X., Malard P., Ben X. (2019). Probiotics maintain intestinal secretory immunoglobulin A levels in healthy formula-fed infants: A randomised, double-blind, placebo-controlled study. Benef. Microbes.

[92] Xu L., Wang Y., Wang Y., Fu J., Sun M., Mao Z., Vandenplas Y. (2016). A double-blinded randomized trial on growth and feeding tolerance with Saccharomyces boulardii CNCM I-745 in formula-fed preterm infants. J. Pediatr. (Rio J.).

[93] Xuan N.N., Wang D., Grathwohl D., Lan P.N., Kim H.V., Goyer A., Benyacoub J. (2013). Effect of a Growing-up Milk Containing Synbiotics on Immune Function and Growth in Children: A Cluster Randomized, Multicenter, Double-blind, Placebo Controlled Study. Clin. Med. Insights Pediatr..

[94] Ziegler E.E., Jeter J.M., Drulis J.M., Nelson S.E., Haschke F., Steenhout P., Brown C., Maire J.C., Hager C. (2003). Formula with reduced content of improved, partially hydrolyzed protein and probiotics: Infant growth and health. Mon. Fur Kinderheilkd..

[95] Jalali S.Z., Shiri M.R., Shirazi M.G. (2020). Effect of probiotics on full intestinal feeding in premature infants: A double blind, clinical trial. Iran. J. Pediatrics.

[96] Meli F., Puccio G., Cajozzo C., Ricottone G.L., Pecquet S., Sprenger N., Steenhout P. (2014). Growth and safety evaluation of infant formulae containing oligosaccharides derived from bovine milk: A randomized, double-blind, noninferiority trial. BMC Pediatr..

[97] Oshiro T., Nagata S., Wang C., Takahashi T., Tsuji H., Asahara T., Nomoto K., Takei H., Nittono H., Yamashiro Y. (2019). Bifidobacterium Supplementation of Colostrum and Breast Milk Enhances Weight Gain and Metabolic Responses Associated with Microbiota Establishment in Very-Preterm Infants. Biomed. Hub..

[98] Ringel-Kulka T., Kotch J.B., Jensen E.T., Savage E., Weber D.J. (2015). Randomized, double-blind, placebo-controlled study of synbiotic yogurt effect on the health of children. J. Pediatr..

[99] Blanton L.V., Barratt M.J., Charbonneau M.R., Ahmed T., Gordon J.I. (2016). Childhood undernutrition, the gut microbiota, and microbiota-directed therapeutics. Science.

[100] Subramanian S., Huq S., Yatsunenko T., Haque R., Mahfuz M., Alam M.A., Benezra A., DeStefano J., Meier M.F., Muegge B.D. (2014). Persistent gut microbiota immaturity in malnourished Bangladeshi children. Nature.

