# Supplementary material for: Probiotic Supplementation for Promotion of Growth in Children: A Systematic Review and Meta-Analysis
Source: Nutrients. 2021 Dec 25;14(1):83. doi: 10.3390/nu14010083 (PMC8746675; doi:10.3390/nu14010083)
Supplement: Supplementary file 1 [file nutrients-14-00083-s001.zip › nutrients-1517130-supplementary.pdf]

Supplementary Material
Probiotic supplementation for promotion of growth in children: a systematic review and meta-analysis

Joseph Catania, Natasha G Pandit, Julie M Ehrlich, Muizz Zaman, Elizabeth Stone, Courtney Franceschi, Abigail Smith, Emily Tanner-Smith, Joseph P. Zackular, Zulfiqar Ahmed Bhutta, Aamer Imdad

Correspondence
Aamer Imdad MBBS, MPH
Department of Pediatrics, Division of Pediatric Gastroenterology, Hepatology and Nutrition, SUNY Upstate Medical University, Syracuse NY 13210, USA; imdada@upstate.edu

Contents

Text S1: Search strategies for databases through November 6, 2020.
Text S2: Extended data input details
Text S3: Results: Study Locations
Table S1: Excluded studies and reasons for exclusion

Participant Characteristics
- Table S2: Participant characteristics in the included studies from low- and middle-income countries
- Table S3: Participant characteristics in the included studies from high income countries

Treatment Characteristics
- Table S4: Treatment and comparison characteristics in the included studies from low- and middle-income countries
- Table S5: Treatment and comparison characteristics in the included studies from high income countries

Pooled Strain Analysis
- Table S6: Pooled analysis based on probiotic strains: Low- and middle-income countries
- Table S7: Pooled analysis based on probiotic strains: High income countries

Funnel Plots
- Figure S1: Funnel Plot: Effect of probiotics on growth: Low- and middle-income countries: Weight
- Figure S2: Funnel Plot: Effect of probiotics on growth: High income countries: Weight
- Figure S3: Funnel Plot: Effect of probiotics on growth: Low- and middle-income countries: Head Circumference
- Figure S4: Funnel Plot: Effect of probiotics on growth: High income countries: Height
- Figure S5: Funnel Plot: Effect of probiotics on growth: High income countries: Head circumference

Risk of Bias Low- and Middle-Income Countries
- Figure S6: Risk of bias in the included studies from low- and middle-income countries for effect of probiotics on the outcome of weight for age

Figure S7: Risk of bias in the included studies from low- and middle-income countries for the outcome of height for age
Figure S8: Risk of bias in the included studies from low- and middle-income countries for the outcome of head circumference
Figure S9: Risk of bias in the included studies from low- and middle-income countries for the outcome of sepsis

Forest Plots Low- and Middle-Income Countries
Figure S10: Forest Plot: Effect of use of probiotics on head circumference in children 0-59 months of age from low- and middle-income countries
Figure S11: Forest Plot: Effect of use of probiotics on BMI in children 0-59 months of age from low- and middle-income countries
Figure S12: Forest Plot: Effect of use of probiotics on weight for height in children 0-59 months of age from low- and middle-income countries
Figure S13: Forest Plot: Effect of use of probiotics on adverse events: Sepsis, in children 0-59 months of age from low- and middle-income countries
Figure S14: Forest Plot: Effect of use of probiotics on adverse events: Vomiting, in children 0-59 months of age from low- and middle-income countries
Figure S15: Forest Plot: Effect of use of probiotics on adverse events: Diarrhea, in children 0-59 months of age from low- and middle-income countries

Forest Plots High-Income Countries
Figure S16: Forest Plot Effect of use of probiotics on weight for age in children 0-59 months of age from high income countries
Figure S17: Forest Plot: Effect of use of probiotics on height for age in children 0-59 months of age from high income countries
Figure S18: Forest Plot: Effect of use of probiotics on weight for length in children 0-59 months of age from high income countries
Figure S19: Forest Plot: Effect of use of probiotics on head circumference for age in children 0-59 months of age from high income countries
Figure S20: Forest Plot: Effect of use of probiotics on BMI in children 0-59 months of age from high income countries
Figure S21: Forest Plot: Effect of use of probiotics on adverse events: Sepsis, in children 0-59 months of age from high income countries
Figure S22: Forest Plot: Effect of use of probiotics on adverse events: Vomiting, in children 0-59 months of age from high income countries
Figure S23: Forest Plot: Effect of use of probiotics on adverse events: Diarrhea, in children 0-59 months of age from high income countries
Figure S24: Forest Plot: Effect of use of probiotics on adverse events: Abdominal pain, in children 0-59 months of age from high income countries
Figure S25: Forest Plot: Effect of use of probiotics on adverse events: Flatulence, in children 0-59 months of age from high income countries

Risk of Bias High-Income Countries
Figure S26: Risk of bias in the included studies from high income countries for effect of probiotics on the outcome of weight for age

Figure S27: Risk of bias in the included studies from high income countries for effect of probiotics on the outcome of height for age

Figure S28: Risk of bias in the included studies from high income countries for effect of probiotics on the outcome of weight for length

Figure S29: Risk of bias in the included studies from high income countries for effect of probiotics on the outcome of head circumference

Figure S30: Risk of bias in the included studies from high income countries for effect of probiotics on the outcome of BMI

Figure S31: Risk of bias in the included studies from high income countries for effect of probiotics on the outcome of sepsis

Figure S32: Subgroup and Sensitivity analyses in high income countries

Text S1: Search strategies for databases through November 6, 2020.
Medline Strategy using PubMed

(((("Probiotics"[Mesh] OR "Synbiotics"[Mesh] OR "Prebiotics"[Mesh] OR Probiotic*[tiab] OR synbiotic*[tiab] OR prebiotic*[tiab]))) AND ((infant*[tiab] OR baby[tiab] OR babies[tiab] OR newborn*[tiab] OR neonat*[tiab] OR neo nat*[tiab] OR child*[tiab] OR toddler*[tiab] OR adolescen*[tiab] OR teen*[tiab] OR teenager*[tiab] OR youth[tiab] OR juvenile*[tiab] OR "Infant"[Mesh] OR "Child"[Mesh] OR "Adolescent"[Mesh]))) NOT (("Animals"[Mesh] NOT ("Animals"[Mesh] AND "Humans"[Mesh])))

CINAHL Strategy

(((MH "Probiotics"OR MH "Prebiotics") OR (TI (probiotic* OR synbiotic* OR prebiotic*)) OR (AB (probiotic* OR synbiotic* OR prebiotic*)))

AND

(((MH “Infant” OR MH “Infant, Newborn" OR MH “Child” OR MH “Child, Preschool” OR MH “Adolescence”)) OR (TI (infant* OR baby OR babies OR newborn* OR neonat* OR neo nat* OR child* OR toddler* OR adolescen* OR teen* OR teenager* OR youth OR juvenile*)) OR (AB (infant* OR baby OR babies OR newborn* OR neonat* OR neo nat* OR child* OR toddler* OR adolescen* OR teen* OR teenager* OR youth OR juvenile*))))

NOT

(MH “Animals” AND (MH "Animals" AND MH "Humans"))))

Limiter: Exclude MEDLINE records

Cochrane CENTRAL Strategy

1 MeSH descriptor: [Infant] explode all trees

2 MeSH descriptor: [Child] explode all trees

3 MeSH descriptor: [Adolescent] explode all trees

4 infant*:ti,ab OR baby:ti,ab OR babies:ti,ab OR newborn*:ti,ab OR neonat*:ti,ab OR neo nat*:ti,ab OR child*:ti,ab OR toddler*:ti,ab OR adolescen*:ti,ab OR teen*:ti,ab OR youth:ti,ab OR juvenile*:ti,ab

5 #1 OR #2 OR #3 OR #4

6 MeSH descriptor: [Probiotics] explode all trees

7 MeSH descriptor: [Prebiotics] explode all trees

8 MeSH descriptor: [Synbiotics] explode all trees

9 probiotic*:ti,ab OR prebiotic*:ti,ab OR synbiotic*:ti,ab

10 #6 OR #7 OR #8 OR #9

11 MeSH descriptor: [Animals] explode all trees

12 MeSH descriptor: [Humans] explode all trees

13 (#11 NOT (#11 AND #12))

14 #5 AND #10 NOT #13

15 "accession number" near pubmed

16 #14 NOT #15

Scopus Strategy

TITLE-ABS (probiotic* OR prebiotic* OR synbiotic*) AND TITLE-ABS (infant* OR baby OR babies OR newborn* OR neonat* OR "neo nat*" OR child* OR toddler* OR adolescen* OR teen* OR youth OR juvenile) AND NOT INDEX (medline)

Embase Strategy

1 'probiotic agent'/exp OR 'prebiotic agent'/exp OR 'synbiotic agent'/exp

2 probiotic*:ti,ab OR synbiotic*:ti,ab OR prebiotic*:ti,ab

3 #1 OR #2

4 'infant'/exp OR 'newborn'/exp OR 'child'/exp OR 'toddler'/exp OR 'adolescent'/exp OR 'adolescence'/exp OR 'juvenile'/exp

5 infant*:ti,ab OR baby:ti,ab OR babies:ti,ab OR newborn*:ti,ab OR neonat*:ti,ab OR 'neo nat*':ti,ab OR child*:ti,ab OR toddler*:ti,ab OR adolescen*:ti,ab OR teen*:ti,ab OR youth:ti,ab OR juvenile*:ti,ab

6 #4 OR #5

7 #3 AND #6Pr

8 #7 NOT ([animals]/lim NOT [humans]/lim)

9 #8 NOT [medline]/lim

LILACS

(mh:(probiotics)) OR (mh:(prebiotics)) OR (mh:(synbiotics)) OR ((ti:(probiotic*)) OR (ti:(prebiotic*)) OR (ti:(synbiotic*)) OR (ab:(probiotic*)) OR (ab:(prebiotic*)) OR (ab:(synbiotic*))) AND ((mh:(Infant)) OR (mh:(Child)) OR (mh:(Adolescent)) OR ((ti:(infant* OR baby OR babies OR newborn* OR neonat* OR "neo nat*" OR child* OR toddler* OR adolescen* OR teen* OR youth OR juvenile)) OR ((ab:(infant* OR baby OR babies OR newborn* OR neonat* OR "neo nat*" OR child* OR toddler* OR adolescen* OR teen* OR youth OR juvenile))))) AND NOT ((mh:(Animals)) AND NOT ((mh:(Animals)) AND (mh:(Human))))

Text S2: Extended data input details
If a study had multiple intervention arms and the only difference between the arms were the strain of the probiotics used, the intervention arms were combined and compared against the control group. This was done to avoid double counting the control group. If a study had run two different arms with both a treatment and control group, those arms were taken individually and split in our meta-analyses. This was done for 2 studies[1,2]. Some studies had data given data in different forms. If the data was not able to be entered into RevMan, it was converted. All conversions were done in RevMan besides when data was presented as median (IQR or range). We used a reputable calculator to convert data to a RevMan acceptable form[3].

Text S3: Results: Study Locations
Nine studies were conducted in the United States[4-12]. Six studies were conducted in Spain[13-18] and France[19-24]. Five studies were conducted in Germany[21,25-28], India[29-33], and Turkey[34-38]. Four studies were conducted in China[39-42], South Africa[1,2,43,44], and Italy[45-48]. Three studies were conducted in Finland[49-51], Greece[52-54], Israel[55-57], and the United Kingdom[58-60]. Two studies were conducted in Australia[61,62], New Zealand[61,63], Netherlands[21,64], Indonesia[65,66], Iran[67,68], Poland[69,70], and Japan[71,72]. One study each was conducted in Mexico[73], Portugal[73], Croatia[74], Estonia[75], Austria[26], Serbia[26], Philippines[76], Sweden[77], Switzerland[78], Taiwan[79], Thailand[80], and Vietnam[81].

Table S1. Excluded studies and reasons for exclusion

Study Name	Reason for Exclusion
Abdulkadir 2016[82]	No relevant outcomes were reported
Abramova 2015[83]	Wrong intervention (not probiotics)
Actrn 2019[84]	Trial not yet finished
Actrn 2020[85]	Trial not yet finished
Aflatoonian 2019[86]	Wrong study population (malnourished)
Akar 2017[87]	Incompatible study design
Aydin 2012[88]	No relevant outcomes were reported
Baglatzi 2016[89]	Incorrect intervention and comparator pairing (no control group, only an observational reference is included that was not randomized)
Bakker-Zierikzee 2005[90]	No relevant outcomes were reported
Bakker-Zierikzee 2006[91]	No relevant outcomes were reported
Barclay 2003[92]	Abstract only; which contained limited data
Berggren 2003[93]	No relevant outcomes were reported
Binns 2007[94]	No relevant outcomes were reported

Bocquet 2013[95]	Incorrect intervention and comparator pairing (no probiotic intervention)
Braga 2011[96]	No relevant outcomes were reported
Campeotto 2011[97]	Unable to locate full study
Campoy 2018[98]	Wrong intervention (not probiotics)
Castanet 2020[99]	Wrong intervention
Cazzola 2010[100]	No relevant outcomes were reported
Cazzola 2010 (abstract)[100]	No relevant outcomes were reported
Chandel 2010	Study not locatable
Chau 2015[101]	Typo in results and could not contact authors
Chen 2010[102]	No relevant outcomes were reported
Chi 2019	No relevant outcomes were reported
Chou 2010[104]	Incompatible study design
Chubarova 2017[105]	No relevant outcomes were reported
Cooper 2015	Study not locatable
Costeloe 2015[106]	No relevant outcomes were reported
Cox 2010[107]	No relevant outcomes were reported
Ctri 2009[108]	Trial not yet finished
Ctri 2010[109]	Trial not yet finished
Ctri 2012[110]	Trial not yet finished
Ctri 2012[111]	Trial not yet finished
Ctri 2017[112]	Trial not yet finished
daCostaRibeiro 2015[113]	Wrong intervention (prebiotics)
DiPierro 2020[114]	No relevant outcomes were reported
Drks 2018[115]	Trial not yet finished
Dupont 2010[116]	Incorrect intervention and comparator pairing
Famouri 2014[117]	Wrong study population (malnourished)
Firmansyah 2011[118]	Wrong intervention (containing LCPUFA + synbiotics)
Fisberg 2000	Study not locatable
Fisberg 2002[73]	Wrong study population (malnourished)

Fleming 2015[119]	No relevant outcomes were reported
Fonolla 2017[120]	No results reported in abstract, full study not found, similar population as reported in Gil Campos 2012
Galpin 2005[121]	Wrong patient population
Garland 2011[122]	No relevant outcomes were reported
Gibson 2009[123]	Wrong intervention (containing LCPUFA + probiotics)
Gomez-Rodriguez 2019[124]	Incorrect intervention and comparator pairing (both groups received probiotics)
Gonchar 2015[125]	Primary language translation error
Gonchar 2016[126]	Primary language translation error
Grenov 2017[127]	Wrong study population (malnourished)
Greuter 2020[128]	Wrong study design (meta-analysis)
Hariharan 2016[129]	No relevant outcomes were reported
Hartel 2014[130]	Incompatible study design (observational)
Hartel 2017[131]	Incompatible study design (observational)
Hascoet 2007	Study not found
Hishiki 2020[132]	Wrong intervention (heat killed)
Hojsak 2016[133]	No relevant outcomes were reported
Holscher 2012[134]	No relevant results were reported
Hu 2010[135]	No relevant outcomes were reported and primary language translation error
Hurkala 2020[136]	No relevant outcomes were reported
Irct201210209568N 2013[137]	Trial not yet finished
Irct2016061128386N 2016[138]	Trial not yet finished
Isolauri 2000[139]	No relevant outcomes were reported
Isrctn 2005[140]	Trial not yet finished
Isrctn 2008[141]	Trial not yet finished
Isrctn 2012[142]	Trial not yet finished
Jones 2012[143]	Wrong study population (adults)

Jones 2018[144]	Wrong study population (greater than 5 years old)
Jprn 2015[145]	Trial not yet finished
Kara 2019[146]	Wrong study population (malnourished)
Kerac 2009[147]	Wrong study population (malnourished)
Kianifar 2018[148]	Incorrect intervention and comparator pairing (intervention includes synbiotics + vitamins but not found in control placebo)
Kim 2010[149]	No relevant outcomes were reported
Kitajima 1997[150]	No relevant outcomes were reported
Kon 2014[151]	Incompatible study design
Kuitunen 2009[152]	Duplicate study population as included study (Kukkonen 2008)
Langhendries 1995[153]	No relevant results were reported
Lazou Ahren 2020[154]	No relevant outcomes were reported
Lee le 2015[155]	Incorrect intervention and comparator pairing: Both groups contained probiotics, experimental group also contained GOS and FOS
Lin 2005[156]	No relevant outcomes were reported
Mai 2020[157]	Wrong study design (not an RCT)
Maldonado-Lobon 2014[158]	Incompatible study design
Maldonado-Lobon 2015[159]	Incompatible study design (more observational than a randomized control trial, this is a long term follow up study that was initially RCT)
Manzoni 2011[160]	Wrong interventions (testing more than just probiotics - lactoferrin also included)
Marangione 2008	Study not locatable
Marissen 2019[161]	Only a trial protocol and clinical trial still active
Materna 2010[162]	Trial not yet finished
Merenstein 2010 (a)[163]	No relevant outcomes were reported
Merenstein 2010 (b)[164]	No relevant outcomes were reported
Meyer 2015[165]	Incompatible study design (not randomized controlled trial)
Modi 2010[166]	Wrong intervention (prebiotics only)
Muraro 2012[167]	No relevant outcomes were reported
Nakamura 2010[168]	Wrong intervention (prebiotics only)
Nct 2008[169]	Wrong outcomes

Nct 2009[170]	Wrong intervention
Nct 2010[171]	Wrong outcomes
Nct 2010[172]	Wrong outcomes
Nct 2011[173]	Wrong comparator
Nct 2011[174]	No results
Nct 2012[175]	No results
Nct 2012[176]	Wrong intervention
Nct 2012[177]	Wrong outcomes
Nct 2013[178]	Wrong comparator
Nct 2013[179]	Trial not yet finished
Nct 2014[180]	Wrong outcomes
Nct 2014[181]	Trial not yet finished
Nct 2014[182]	Trial not yet finished
Nct 2014[183]	Trial not yet finished
Nct 2014[184]	Wrong outcomes
Nct 2014[185]	Wrong outcomes
Nct 2014[186]	Wrong outcomes
Nct 2015[187]	Wrong outcomes
Nct 2015[188]	Wrong outcomes
Nct 2016[189]	Trial not yet finished
Nct 2017[190]	Wrong intervention
Nct 2018[191]	Trial not yet finished
Nct 2018[192]	Trial not yet finished
Nct 2018[193]	Trial not yet finished
Nct 2019[194]	Trial not yet finished
Nct 2019[195]	Wrong intervention
Nct 2019[196]	Trial not yet finished
Nct 2020[197]	Wrong intervention
Nopchinda 2002[198]	Wrong study design
Ntr 2008[199]	Trial not yet finished
Ntr 2008[200]	Trial not yet finished
Ntr 2010[201]	Wrong intervention
Oberhelman 1999[202]	No relevant outcomes were reported
Pastor-Villaescusa 2020[203]	Wrong patient population
Patole 2016[204]	Incorrect intervention and comparator pairing (both groups receive probiotics)

Pecquet 2012[205]	Wrong intervention (not probiotics)
Pehlevan 2020[206]	No relevant outcomes were reported
Picaud 2020[207]	Wrong intervention (no probiotics)
Rinaldi 2012[208]	No relevant outcomes were reported (primary outcome is NEC, as full text is unavailable, unable to confirm whether relevant outcomes are reported)
Rinne 2005[209]	Wrong intervention (no probiotics)
Rodriguez-Herrera 2019[210]	Wrong intervention (prebiotics only)
Rojas 2012[211]	No relevant outcomes were reported
Rubaltelli 2000[212]	Wrong outcomes
Sadowska-Krawczenko 2012[213]	No results were reported
Saran 2002[214]	Incompatible study design
Sari 2012[215]	Duplicate study population as included study, Sari 2010
Sazawal 2010[216]	Wrong outcomes
Scalabrin 2017[217]	Duplicate study population as included study, Scalabrin 2009
Serce 2013[218]	No relevant outcomes were reported
Silva 2008[219]	Incompatible study design
Strong 2015[220]	No relevant outcomes were reported
Strus 2018[221]	No relevant outcomes were reported
Szajewska 2013[222]	No relevant results were reported
Tewari 2015[223]	No relevant outcomes were reported
Totsu 2018[224]	Duplicate study population as included study, Totsu 2014
Urbanska 2016[225]	No relevant outcomes were reported
Vakiliamini 2020[226]	No relevant outcomes were reported
Van Niekert 2015[227]	Relevant outcomes only reported in subset of patient population (patients with NEC)
Vandenplas 2017[228]	Incompatible study design
Veereman-Wauters 2011[229]	Wrong intervention (prebiotics only)

Victoria 2017[230]	Unable to locate full study
Vray 2018[231]	Wrong intervention (not probiotics)
Wall 2019[232]	Incorrect intervention and comparator pairing (differences in formula composition)
Wu 2020[233]	No relevant outcomes were reported
Yamasaki 2012[234]	Wrong comparator group (both groups received probiotics within different time windows after birth, and there is no control group)
Ye 2009[235]	Trial not yet finished
Zhang 2017[236]	Incorrect intervention and comparator pairing (control does not contain mosapride while experimental containing mospride + probiotics)
Ziegler 2007[237]	Wrong Intervention (prebiotics only)
Ziegler 2007 (abstract)[237]	Wrong intervention

Table S2. Participant characteristics in the included studies from low- and middle-income countries

**Author**	**Setting**	**Country**	**Inclusion Criteria**	**Average age at enrollment**	**Nutritional Status**
Agustina 2013	Community	Indonesia	Apparently healthy infants, not being breastfed (and if consuming milk, calcium intake was <75% of the age-specific recommended daily allowances) were eligible	59.4 months +/- 14.3 months	healthy
Amini 2017	Hospital	Iran	All premature newborns weighing 750 - 1500 g or less than 32 weeks' gestation who received antibiotics and total parenteral nutrition in NICU of Vali Asr Hospital (Tehran, Iran).	<1 month	premature/ LBW
Batac 2005	Community	Philippines	Healthy infants with a Z score >-3 SD	12.5 months	healthy

			between 11-15 months old.		
Cooper 2016	Hospital	South Africa	Healthy, full-term, newborn infants (between 37 weeks and 42 weeks gestation), ≤3 days old, weighing between 2500 g and 4500 grams, being a singleton birth); recruited from HIV-positive mothers who had elected to feed their child exclusively with formula beginning from birth	<1 month	healthy
Cui 2019	Hospital	China	Formula-fed preterm infants admitted within 12 hours of birth whose gestational age ≥30 and <37 weeks; birthweight ≥1500g and ≤2000g with stable vital sign and hemodynamic parameters.	<1 month	premature/ LBW
Demirel 2013	Hospital	Turkey	Preterm infants of gestational age ≤32 weeks and birthweight ≤1500 grams who survived to start enteral feeding	<1 month	premature/ LBW
Dilli 2015	Hospital	Turkey	Very Low Birth Weight infants (with a gestational age of <32 weeks and a birth weight of <1500 grams) who were born at or transferred to the NICU within the first week of life (fed enterally prior to inclusion) were included.	<1 month	premature/ LBW
Guney Varal 2018	Hospital	Turkey	Preterm infants with a gestational age of ≤32	<1 month	premature/ LBW

			weeks and a birth weight of ≤1500 grams.		
Jalali 2020	Hospital	Iran	Preterm infants born at < 36 weeks, birth weight 1000 to 2500 gr and postnatal birth weight less than or equal to two weeks with intestinal feeding.	<1 month	healthy
Li 2019	Hospital	China	Infants with gestational age of 37–42 weeks at birth, birth weight >2.500 g and <4.000 g, absence of chronic illness, with a parent or legal representative who could speak and understand Chinese were eligible:<br>-For the formula-fed group: healthy infants of mothers who could not or voluntarily completely refrained from breastfeeding at inclusion (infant age 21 ± 7 days)<br>-For the breastfed group: having been exclusively breastfed from birth and mothers intending to breastfeed >80% to age at least 4 months	<1 month	healthy
Mitra 2014	Community	India	Healthy full-term infants were eligible.	<1 month	healthy
Panigrahi 2017	Community	India	Eligible infants needed to be at least 2000 grams at birth, breastfed by 24th hour of life, and able to tolerate oral feeds. Informed consent by parent or guardian.	<1 month	healthy

Panigrahi 2008	Hospital	India	Healthy newborn infants (>35 weeks of gestational age and >1800 grams weight at birth) were eligible for enrollment. Inclusion criteria included all of the babies delivered in the unit (including those by caesarean section) that were >12 and <72 hours old, were not taking antibiotics at time of enrollment, had little likelihood of receiving antibiotics later in the hospital stay, and were able to tolerate oral feeds and the establishment of breast-feeding before enrollment.	<1 month	healthy
Saengtawes in 2014	Hospital	Thailand	Preterm infants with gestational age less than or equal to 34 weeks and birth weight less than or equal to 1,500 grams were eligible.	<1 month	premature/ LBW
Sari 2010	Hospital	Turkey	Preterm neonates with a gestational age <33 weeks or birth weight <1500 grams, who survived to feed enterally were eligible for enrollment.	<1 month	premature/ LBW
Sazawal 2010	Community	India	Children 1 to 3 years old, likely to remain in the area, not experiencing severe malnutrition, and nonallergic to milk were elibible.	22.55 months	healthy

Serce 2013	Hospital	Turkey	Very Low Birth Weight infants (with a gestational age ≤32 weeks; birth weight ≤1500 grams) who survived to feed enterally were eligible for the trial.	<1 month	premature/ LBW
Sur 2011	Community	India	Eligible children, aged 1–5 years, were enrolled with verbal consent of parents for participation of their children in the study. They were enrolled through a demographic census conducted at the initiation of the study. The census was used to define 100 contiguous geographical clusters that served as the unit of randomization. About 40 children were included in each.	35.89 months	healthy
Surono 2011	Community	Indonesia	To be eligible, healthy children required informed consent. Eighty one apparently healthy preschool children (39 boys and 40 girls, between 15 and 54 months age, mean age 33 months) who lived in Teluk Naga sub District, Tangerang, Banten Province, Indonesia were eligible and enrolled.	33 months	healthy
Urban 2008	Hospital	South Africa	Normally grown (birth weight 2500–4200 grams), term (gestation 37–42 weeks) male or female infants born to	<1 month	healthy

			HIV-infected women who had elected not to breast-feed were eligible to enroll.		
Van Niekerk 2014	Hospital	south Africa	Consecutive HIV-positive or HIV-negative mothers who gave birth to a premature baby with a birth weight between 500 and 1250 grams at TBCH and consented to participate in the study were eligible to enroll. Only mothers who decided to breastfeed after counseling, regardless of their HIV status were included. HIV-positive mothers who were on the prevention of mother-to-child transmission treatment schedule received nevirapine and zidovudine as well as those who received highly active antiretroviral therapy (HAART) were enrolled in the study, as well as HIV-exposed infants that received antiretroviral (ARV) medication.	<1 month	premature/ LBW
Velaphi 2008	Hospital	South Africa	To be eligible, the infant's mother was diagnosed with HIV by standard HIV testing and decided on exclusive formula-feeding for 4 months and the infant was born at term (37– 42 weeks), had a normal	<1 month	healthy

			birth weight (2500 – 4200 grams), and the infant was 1 week of age at time of enrollment. In cases of multiple births enrollment occurred only if all siblings met the inclusion criteria.		
Xiao 2019	Hospital	China	To be eligible to enroll, infants were of single birth at gestational age ≥37 weeks (i.e. non-preterm infants), birth weight >2,500 grams; appropriateweight between P20-P80 at the inclusion visit, in accordance with the weight percentile standards for children up to 6 years old in the Shanghai urban areas. Additionally, formula feeding had to represent >80% of daily food intake at the inclusion visit. There had to be an absence of gastrointestinal diseases within 1 month prior to the inclusion visit as well as parents agreement to use one of the recommended infant formulas (i.e. without probiotics, without fructooligosaccharide, with less than 2 grams of galactooligosaccharide /100 grams).There also had to be a capacity of the parent or legal tutor to understand the	4.89 months	healthy

			protocol and fill out the infant's diary and consent form had to be signed by at least one of the parents or by the legal tutor properly informed of the study.		
Xu 2016	Hospital	China	To be eligible to enroll, infants had to be hospital-born formula-fed, with a gestational age of 30---37 weeks and a birth weight between 1500 and 2500 grams.	<1 month	premature/ LBW
Xuan 2013	Community	Vietnam	To be eligible, infants had to be between 18 and 36-months-old, were not being breastfed, had no congenital or chronic diseases, and were not consuming any commercial products containing probiotics or prebiotics during the study period.	30.12 months	healthy

Table S3. Participant characteristics in the included studies from high income countries

**Author**	**Setting**	**Country**	**Inclusion Criteria**	**Average age at enrollment**	**Nutritional Status**
Abrahamse-Berkeveld 2016	Hospital	Germany	Infants younger than age 35 days who are exclusively formula-fed and of normal birth weight for gestational age and sex (birth weight between the 10th and 90th percentile according to	<1 month	healthy

			local standards on weight-for-gestational age values).		
Ahrens 2018	Community	Germany, Austria, Serbia	Healthy term newborns <28 days of life with gestational age of 37 weeks and birth weight between 2500 and 4500 grams.	<1 month	healthy
Al-Hosni 2011	Hospital	US	All premature infants with birth weight 501 to 1000 grams, who were appropriate for gestational age (AGA), and less than or equal to 14 days of age at the time of feeding initiation.	<1 month	premature/ LBW
Allen 2010	Community	UK	Women aged 16 years or older with a normal singleton pregnancy attending antenatal clinics in hospitals or general practice surgeries.	<1 month	healthy
Aloisio 2018	Community	Italy	Healthy infants within 15 days from birth and born adequate for gestational age.	<1 month	healthy
Bazanella 2017	Community	Germany	Healthy term infants born by vaginal or cesarean delivery and	<1 month	healthy

			breastfed, formula-fed, or mixed-fed (breast and formula in parallel).		
Bin-Nun 2005	Hospital	Israel	Preterm neonates, <1500 grams birth weight, who were admitted to the neonatal intensive care unit of the Shaare Zedek Medical Center between September 2001 and September 2004, and who began feeding on a weekday, were recruited for study on the day that they were to begin feeds.	<1 month	premature/ LBW
Cekola 2015	Community	US	Healthy, full-term (≥37 weeks gestation; ≥2500 and ≤4500 grams birth weight), singleton-birth infants of any ethnicity who had been exclusively formula-fed for at least 3 days prior to potential enrollment	<1 month	healthy

Chouraqui 2008	Hospital	France	Infants that were healthy, full-term (gestational age between 37 and 42 weeks), ≤14 d old, singletons, and weighed between 2500 and 4500 grams; recruited from mothers who had chosen not to breastfeed and had decided to feed their infants exclusively with formula from the time of enrollment until infants were at least 16 weeks old	<1 month	healthy
Chrzanowska-Liszewska 2012	Hospital	Poland	Preterm neonates with birth weight >1000 grams, gestational age <32 weeks with absence of any disease other than those linked to prematurity, and commencement of enteral formula feeding before enrollment	<1 month	premature/ LBW
Costalos 2003	Hospital	Greece	Infants had a gestational age between 28 and 32 weeks and did not have	<1 month	premature/ LBW

			major gastrointestinal abnormalities; they were not receiving antibiotics or antifungals and they were not receiving breast milk.		
Costeloe 2016	Hospital	UK	Infants born between 23 weeks and 0 days, and 30 weeks and 6 days of gestation from 24 hospitals within 60 miles of London were eligible.	<1 month	premature/ LBW
Dekker 2009	Community	New Zealand	Infants with asthma, hay fever or eczema treated by a doctor in either biological parent.	<1 month	healthy
Escribano 2018	Hospital	Spain	Healthy-term newborns (≥37 weeks), with normal birth weight (3rd to 97th percentiles for gestational age), normal growth curve (according to current Spanish references) and at ≤3 months of age at enrollment were eligible. They had to be exclusively	<1 month	healthy

			formula-fed at enrollment, with ≤30 days of breastfeeding (stopped at least 15 days before inclusion), and ≤48 hours of feeding with pre- or probiotic-supplemented infant formulas (with a washout period of 7 days with control formula).		
Gil-Campos 2012	Hospital	Spain	Healthy, one-month-old infants who were exclusively formula-fed.	1 month	healthy
Harvey 2014	Community	US	Full-term infants aged from birth to 15 days that could receive study formula as their sole source of nutrition throughout the study duration.	10.55 months	healthy
Hays 2015	Hospital	France	Infants with a gestational age at birth of between 25 weeks and 31 weeks, with a birth weight of between 700 g and 1600 g were eligible if admission to a participating unit within	<1 month	premature/ LBW

			seven days of life and enteral feeding was initiated before the fifth day of life		
Hojsak 2010	Community	Croatia	Children attending day care centers in Zagreb area whose parents or legal guardians provided written informed consent (and also who did not meet any of the exclusion criteria).	52.75 months	healthy
Indrio 2008	Hospital	Italy	Healthy preterm infants who are appropriate for gestational age, with normal Apgar scores were enrolled on days 3 to 5 of life.	<1 month	healthy
Jacobs 2003	Hospital	Australia, New Zealand	Infants born <32 completed weeks' gestation and weighing <1500 grams were eligible for enrollment within 72 hours of birth.	<1 month	premature/ LBW
Kankaanpa¨a¨ 2002	Community	Finland	Infants referred to a pediatric clinic on the basis of atopic eczema (all fulfilled the Hanifin criteria	5.2 months	healthy

			for atopic eczema). All the infants were exclusively breastfed before the eczema symptoms having had no exposure to any infant substitute formula.		
Kukkonen 2008	Community	Finland	Mothers were eligible if at least one parent of the unborn child had a physician-diagnosed allergic disease, as evaluated in telephone interviews by trained personnel.	<1 month	healthy
Lin 2008	Hospital	Taiwan	Very Low Birth Weight preterm infants (gestational age: <34 weeks; birth weight: <1500 grams) who survived to feed enterally were eligible.	<1 month	premature/ LBW
Luoto 2010	Community	Finland	Pregnant women with at least one close relative (mother, father or sibling) with atopic dermatitis, allergic rhinitis or asthma were eligible.	<1 month	healthy

Maldonado 2010	Hospital	Spain	6-momth old children who were fed exclusively with formula at the moment of recruitment were eligible.	Study says 6-month-old children were included in study, but data is based on consumption from t0 to t6	healthy
Maldonado 2012	Community	Spain	Families that lived in proximity to the hospitals whose mothers had delivered their babies at the hospital and/or made regular visits to the pediatrician were considered for the study and contacted by the research nurse during scheduled visits to the hospital. Before the inclusion, infants received a physical examination and their clinical records were consulted for previous diseases and pharmacological treatments. Healthy 6-month-old infants who were exclusively formula fed were recruited.	6.5 months	healthy

Maldonado 2019	Community	Spain	Healthy infants one-month of age who were exclusively feeding with infant formula were included in the study after the parents or caregivers gave written consent.	<1 month	healthy
Manzano 2017	Community	Spain	Healthy, full-term (> 37 weeks gestation at birth) and aged between 3 to 12 months were eligible for recruitment.	6.375 months	healthy
Meli 2014	Hospital	Italy	Age ≤14 days at enrollment, weight 2500–4500 g, gestational age ≥37 weeks at birth, and singleton pregnancy	<1 month	healthy
Millar 1993	Hospital	UK	Preterm infants with a gestational age of 33 weeks or less who were admitted to the neonatal unit of the Princess Anne Hospital, Southampton between September 1, 1991 and January 31, 1992 were studied.	<1 month	premature/ LBW
Mohan 2008	Hospital	Germany	Preterm infants born with a	<1 month	premature/ LBW

			gestational age of 37 week were eligible.		
Oshiro 2019	Hospital	Japan	Infants born between 24 and 31 weeks of gestation and with body weights <1,500 g who had been admitted to the NICU	<1 month	Premature/ LBW
Papagaroufalis 2014	Hospital	Greece	Only infants whose parents/legal guardians had decided to exclusively feed them formula at the time of recruitment and had signed the informed consent document were assessed for eligibility to enter the trial. Those fulfilling all the inclusion criteria and having none of the exclusion criteria were enrolled in the study. Inclusion criteria were being healthy, being born at term (>37 weeks), being >72 hours old at the time of enrollment, being under the care of a	<1 month	healthy

			pediatrician or other qualified healthcare professional, and having had >1 post-natal visit with a healthcare provider.		
Puccio 2007	Community	Italy	Full-term, newborn infants whose mothers had decided not to breast feed beyond the 14th day of birth were enrolled and randomly assigned to one of two groups of 69 infants each. At the time of enrollment, the infants had to be 14 days old, weigh 2500 – 4500 grams, and be singletons.	<1 month	healthy
Radke 2017	Community	Germany, France, Netherlands	Being fullterm (between 37 and 42 weeks gestation); less than 14 days old at the time of enrollment; weighing between 2500 and 4500 grams; and having their legal representative's informed consent were eligibility requirements.	<1 month	healthy

Ringel-Kulka 2015	Community	United States	A child must have attended the CCC at least 5 days a week for >4 hours a day. Given the increase in infectious illness experienced by children in child care settings, restricting to children enrolled 5 days a week increased the potential for observing an effect of synbiotics on incidence or duration of illness. Children were required to be healthy at the time of enrollment.	29 months	Healthy
Rouge 2009	Hospital	France	A gestational age <32 weeks, a birth weight <1500 grams, a postnatal age ≤2 week, the absence of any disease other than those linked to prematurity, and the start of enteral feeding before inclusion were eligibility requirements.	<1 month	premature/ LBW

Roze 2012	Hospital	France	Infants were eligble if they had a gestational age >37 weeks, a postnatal age less than 3 days, and were fed from birth with probiotic- and prebiotic-free infant formula, and without human milk before inclusion.	<1 month	healthy
Saavedra 2004	Community	United States	All parents of children aged between 3 and 24 months attending 27 daycare centers from the metropolitan area of Baltimore were approached for participation. Children/infants were selected on the basis of their parents' willingness to participate in the study.	6.73 months	healthy
Scalabrin 2009	Community	United States	infants included in the study were of 38 to 42 weeks gestational age, with birth weight ≥2500 grams and solely formula-fed at least 24	<1 month	healthy

			hours prior to randomization.		
Simeoni 2016	Community	Switzerland	Healthy infants (of mothers who did not breastfeed in the control and intervention groups, but brreastfed infants were used a reference group). They had to be full term infants (≥ 37 weeks gestation; ≤ 42 weeks gestation). The birth weight needed to be between 2500g and 4500 grams. For the Formula fed groups, the infant's mother had elected, before the 14th day of their child's life, not to continue breastfeeding (no breastfeeding after the 14th day of the child's life). For the Breastfed group, the infant's mother had elected to fully breastfeed her baby, from enrollment to at least 3 months	<1 month	healthy

			of age. Additionally, the study needed to obtain infant's legal representative's informed consent.		
Smilowitz 2017	Community	United States	Women 21 to 45 years of age, in their third trimester of pregnancy or had delivered an infant within the past 4 days who were planning to exclusively breastfeed their infants for at least the first 3 months postnatal were eligible to enroll. Additionally they must have lived in a developed nation for the past 10 years, did not plan to administer probiotic supplements to their infants during the study duration unless they were allocated to the B. infantis group. They must not have	<1 month	healthy

			been diagnosed with any chronic metabolic disease or obesity, and were non-smokers. Infant gestational age at birth was ≥37 weeks, and they were without medical complications at birth (such as respiratory distress syndrome, birth defects, and infection). There must have been no exposure to any oral or intravenous antibiotics 72 hours postnatal, and no consumption of infant formula 24 hours prior to the Day 7 postnatal at-home lactation consultation visit. Infants must have been 0-7 days old, delivered by C-section or vaginal delivery, born >37 weeks gestation, without medical complications		

			that would preclude breastfeeding or alter gut microbiota.		
Stratiki 2007	Hospital	Greece	To be eligible, infant gestational age had to be between 27 and 37 weeks, and they had to be in a stable state, formula fed and not suffering from major deformities, such as congenital heart defects or bowel atresia.	<1 month	premature/ LBW
Szajewska 2017	Community	Poland	Full-term infants aged ≤28 days, delivered vaginally between 38 and 42 weeks of gestation, with a birth weight >2,700 g and <4,200 grams, weaned completely from breast milk to	<1 month	healthy

			infant formula at 28 days of age, and with parents or the subject's legal representatives able to speak and understand Polish were elligible to enroll in the study.		
Taylor 2007	Hospital	Australia	Infants of atopic women (maternal atopy was defined as a doctor-diagnosed clinical history of asthma, allergic rhinitis, or eczema plus a positive skin prick test (SPT) to 1 or more common allergens (house dust mite, grass pollens, cat, dog, feathers, molds, and cockroach extracts)) were eligible to enroll.	<1 month	healthy
Thibault 2004	Community	France	Only healthy infants were included and eligible to enroll (age range, 4–6 months), The infants had regular contact with other children in day care centers or	4.9 months	healthy

			were living at home with two or more young siblings.		
Totsu 2014	Hospital	Japan	Very low birth weight infants (birthweight <1500 g) who were born or transferred within 24 h to the participating hospitals during the period from January 2010 to March 2011 were eligible to enroll.	<1 month	premature/ LBW
Underwood 2009	Hospital	USA	Eligible infants were less than 35 weeks gestation, had a birth weight of 750-2000 grams born in or transferred to University of California Davis Medical Center within first week of life less than eight days of age at the time of enrollment.	<1 month	premature/ LBW
Vendt 2006	Community	Estonia	Twenty healthy term infants (60 boys) aged from 0 to 2 months on formula for at least half of their daily feedings were eligible to enroll. An infant was recruited	1.33 months	healthy

			into the study only then when the amount of breast milk was still insufficient after a week of trying to breast feed.		
Vlieger 2009	Community	Netherlands	To be eligible, infants had to be born after 37 weeks of gestation and had to be aged <7 days at the time of enrollment.	<1 month	healthy
Weizman 2005	Community	Israel	Healthy term infants, 4 to 10 months old were eligible to enroll.	6.8 months	healthy
Weizman 2006	Community	Israel	Full-term healthy infants, aged 3–65 days, from similar socioeconomic areas were eligible to enroll.	<1 month	healthy
Wejryd 2019	Hospital	sweden	Infants between gestational week 23+0 and 27+6 and a birth weight less than 1000grams were eligible for enrolment within three days after delivery.	<1 month	premature/ LBW
Ziegler 2003	Community	USA	To be eligible to enroll, they had to be male and female infants with birth	<1 month	healthy

			weight between 2500 and 4500 grams.		

Table S4. Treatment and comparison characteristics in the included studies from low- and middle-income countries

Author	Type of Intervention (E.g. Probiotic supplementation or Synbiotics?)	Probiotic Strain	Intervention Form: syrup/liquid/drop (1), tablet (2), powder (3)	Total Duration of Intervention (weeks) (try to round to whole numbers)	Frequency of Intervention (E.g. daily, weekly, etc.)	Dose of probiotic (CFU preferred)	Comparison: placebo (1), standard of care formula fed/breastmilk (2), no intervention (3)
Agustina 2013	Probiotics	Lactobacillus-casei, Lactobacillus-reuteri	syrup/ liquid/ drop	24	2x Daily	5 x 10^8 CFU	placebo
Amini 2017	Probiotics	Lactobacillus-casei, Bifidobacterium-infantis, Lactobacillus-bulgaricus, Lactobacillus-acidophilus, Lactobacillus-rhamnosus, Streptococcus-thermophilus	powder	1	Daily	10^9 CFU	standard of care formula fed/ breastmilk

Batac 2005	Probiotics	Not specified	not specified	8	Daily	Not specified	standard of care formula fed/ breastmilk
Cooper 2016	Synbiotics	Bifidobacterium-animalis-subsp.-lactis	powder	26	ad libitum	10^7 CFU/g	standard of care formula fed/ breastmilk
Cui 2019	Probiotics	Lactobacillus-reuteri	syrup/ liquid/ drop	1	Daily	10^8 CFU	standard of care formula fed/ breastmilk
Demirel 2013	Probiotics	Saccharomyces-boulardii	not specified	6	Daily	5 x 10^9 CFU	standard of care formula fed/ breastmilk
Dilli 2015	Probiotics, Synbiotics	Bifidobacterium-animalis-subsp.-lactis	powder	5	Daily	5 x 10^9 CFU	placebo
Guney Varal 2018	Synbiotics	Lactobacillus-rhamnosus, Lactobacillus-casei, Lactobacillus-plantarum, Bifidobacterium-animalis	syrup/ liquid/ drop	52	Daily	L. rhamnosus (4.1 x 10^8 CFU), L. casei (8.2 x 10^8 CFU), L. plantarum (4.1 x 10^8 CFU), B. animalis (4.1 x 10^8 CFU)	standard of care formula fed/ breastmilk

Jalali 2020	Probiotics	Lactobacillus rhamnosus, Bifidobacterium infantis, and Lactobacillus reuteri	powder	1	Daily	Lactobacillus rhamnosus (1× 10^10 colony-forming unit [CFU]/mL), Lactobacillus reuteri (2×10^9 CFU/mL), and Bifidobacterium longum subsp. infantis (1.5 × 10^9 CFU/mL)	standard of care formula fed/ breastmilk
Li 2019	Probiotics	Lactobacillus-paracasei-ssp.-paracasei	powder	17	Daily	1 x 10^8 CFU/L	standard of care formula fed/ breastmilk
Mitra 2014	Probiotic	Lactobacillus-reuteri, Bifidobacterium-animalis-subsp.-lactis	powder	24	Not specified	Not specified	standard of care formula fed/ breastmilk
Panigrahi 2017	Synbiotics	Lactobacillus-plantarum	syrup/ liquid/ drop	9	Daily	10^9 L. acidophilus, 10^9 B. bifidum	placebo
Panigrahi 2008	Synbiotics	Lactobacillus-plantarum	syrup/ liquid/ drop	1	Daily	10^9 cells	placebo

Saengtawesin 2014	Probiotics	Lactobacillus-acidophilus, Bifidobacterium-bifidum	powder	6	Daily	10^9 L. acidophilus, 1 x 10^9 B. bifidum	standard of care formula fed/ breastmilk
Sari 2010	Probiotics	Lactobacillus-sporogenes	syrup/ liquid/ drop	6	Daily	3.5 x 10^8 CFU	standard of care formula fed/ breastmilk
Sazawal 2010	Synbiotics	Bifidobacterium-animalis-subsp.-lactis	powder	52	Daily	1.9 x 10^7 CFU	standard of care formula fed/ breastmilk
Serce 2013	Probiotics	Saccharomyces-boulardii	powder	6	2x daily	0.5 x 10^9 CFU/kg per dose	placebo
Sur 2011	Probiotics	Lactobacillus-casei	syrup/ liquid/ drop	12	Daily	6.5 X 10^9 CFU	standard of care formula fed/ breastmilk
Surono 2011	Probiotics	Enterococcus-faecium	powder	13	Daily	2.31x10^8 CFU	placebo
Urban 2008	Probiotics	Bifidobacterium-animalis-subsp.-lactis	powder	24	Daily	Not specified	standard of care formula fed/ breastmilk
Van Niekerk 2014	Probitoics	Lactobacillus-rhamnosus, Bifidobacterium-infantis	syrup/ liquid/ drop	4	Daily	3.5*10^10 CFU	placebo

Velaphi 2008	Probiotics	Bifidobacterium-animalis-subsp.-lactis	powder	24	Daily	Not specified	standard of care formula fed/ breastmilk
Xiao 2019	Probiotics	Bifidobacterium-infantis, Bifidobacterium-bifidum, Lactobacillus-helveticus	powder	4	Daily	1.425 × 10^8 CFU of each B. infantis R0033 and B. bifidum R0071, and 9.6×10^9 CFU of L. helveticus R0052	placebo
Xu 2016	Probiotics	Saccharomyces-boulardii	not specified	4	2x Daily	10^9 CFU	no intervention
Xuan 2013	Synbiotics	Lactobacillus-paracasei, Bifidobacterium-longum	powder	20	Daily	10^8 CFU	standard of care formula fed/ breastmilk

Table S5. Treatment and comparison characteristics in the included studies from high income countries

Author	Type of Intervention (E.g. Probiotic supplementation or Synbiotics?)	Probiotic Strain	Intervention Form: syrup/liquid/drop (1), tablet (2), powder (3)	Total Duration of Intervention (weeks) (try to round to whole numbers)	Frequency of Intervention (E.g. daily, weekly, etc.)	Dose of probiotic (CFU preferred)	Comparison: placebo (1), standard of care formula fed/breastmilk (2), no intervention (3)

Agustina 2013	Probiotics	Lactobacillus-casei, Lactobacillus-reuteri	syrup/ liquid/ drop	24	2x Daily	5 x 10^8 CFU	placebo
Amini 2017	Probiotics	Lactobacillus-casei, Bifidobacterium-infantis, Lactobacillus-bulgaricus, Lactobacillus-acidophilus, Lactobacillus-rhamnosus, Streptococcus-thermophilus	powder	1	Daily	10^9 CFU	standard of care formula fed/ breastmilk

Batac 2005	Probiotics	Not specified	not specified	8	Daily	Not specified	standard of care formula fed/ breastmilk
Cooper 2016	Synbiotics	Bifidobacterium-animalis-subsp.-lactis	powder	26	ad libitum	10^7 CFU/g	standard of care formula fed/ breastmilk
Cui 2019	Probiotics	Lactobacillus-reuteri	syrup/ liquid/ drop	1	Daily	10^8 CFU	standard of care formula fed/ breastmilk
Demirel 2013	Probiotics	Saccharomyces-boulardii	not specified	6	Daily	5 x 10^9 CFU	standard of care formula fed/ breastmilk
Dilli 2015	Probiotics, Synbiotics	Bifidobacterium-animalis-subsp.-lactis	powder	5	Daily	5 x 10^9 CFU	placebo
Guney Varal 2018	Synbiotics	Lactobacillus-rhamnosus, Lactobacillus-casei, Lactobacil	syrup/ liquid/ drop	52	Daily	L. rhamnosus (4.1 x 10^8 CFU), L. casei (8.2 x 10^8	standard of care formula fed/ breastmilk

		lus-plantarum, Bifidobacterium-animalis				CFU), L. plantarum (4.1 x 10^8 CFU), B. animalis (4.1 x 10^8 CFU)	
Jalali 2020	Probiotics	Lactobacillus rhamnosus, Bifidobacterium infantis, and Lactobacillus reuteri	powder	1	Daily	Lactobacillus rhamnosus (1× 10^10 colony-forming unit [CFU]/mL), Lactobacillus reuteri (2×10^9 CFU/mL), and Bifidobacterium longum subsp. infantis (1.5 × 10^9 CFU/mL)	standard of care formula fed/ breastmilk

Li 2019	Probiotics	Lactobacillus-paracasei-ssp.-paracasei	powder	17	Daily	1 x 10^8 CFU/L	standard of care formula fed/ breastmilk
Mitra 2014	Probiotic	Lactobacillus-reuteri, Bifidobacterium-animalis-subsp.-lactis	powder	24	Not specified	Not specified	standard of care formula fed/ breastmilk
Panigrahi 2017	Synbiotics	Lactobacillus-plantarum	syrup/ liquid/ drop	9	Daily	10^9 L. acidophilus, 10^9 B. bifidum	placebo
Panigrahi 2008	Synbiotics	Lactobacillus-plantarum	syrup/ liquid/ drop	1	Daily	10^9 cells	placebo
Saengtawesin 2014	Probiotics	Lactobacillus-acidophilus, Bifidobacterium-bifidum	powder	6	Daily	10^9 L. acidophilus, 1 x 10^9 B. bifidum	standard of care formula fed/ breastmilk

Sari 2010	Probiotics	Lactobacillus-sporogenes	syrup/ liquid/ drop	6	Daily	3.5 x 10^8 CFU	standard of care formula fed/ breastmilk
Sazawal 2010	Synbiotics	Bifidobacterium-animalis-subsp.-lactis	powder	52	Daily	1.9 x 10^7 CFU	standard of care formula fed/ breastmilk
Serce 2013	Probiotics	Saccharomyces-boulardii	powder	6	2x daily	0.5 x 10^9 CFU/kg per dose	placebo
Sur 2011	Probiotics	Lactobacillus-casei	syrup/ liquid/ drop	12	Daily	6.5 X 10^9 CFU	standard of care formula fed/ breastmilk
Surono 2011	Probiotics	Enterococcus-faecium	powder	13	Daily	2.31x10^8 CFU	placebo
Urban 2008	Probiotics	Bifidobacterium-animalis-subsp.-lactis	powder	24	Daily	Not specified	standard of care formula fed/ breastmilk
Van Niekerk 2014	Probitoics	Lactobacillus-rhamnosus, Bifidobacterium-infantis	syrup/ liquid/ drop	4	Daily	3.5*10^10 CFU	placebo
Velaphi 2008	Probiotics	Bifidobacterium-animalis-subsp.-lactis	powder	24	Daily	Not specified	standard of care formula fed/ breastmilk

Xiao 2019	Probiotics	Bifidobacterium-infantis, Bifidobacterium-bifidum, Lactobacillus-helveticus	powder	4	Daily	1.425 × 10^8 CFU of each B. infantis R0033 and B. bifidum R0071, and 9.6×10^9 CFU of L. helveticus R0052	placebo
Xu 2016	Probiotics	Saccharomyces-boulardii	not specified	4	2x Daily	10^9 CFU	no intervention
Xuan 2013	Synbiotics	Lactobacillus-paracasei, Bifidobacterium-longum	powder	20	Daily	10^8 CFU	standard of care formula fed/ breastmilk

Table S6. Pooled analysis based on probiotic strains: Low- and middle-income countries

		Weight		Height		Head Circumference		BMI		Weight for Length
**Probiotic**	**Number of Studies**	**Effect Estimate (95% CI)**	**Number of Studies**	**Effect Estimate (95% CI)**	**Number of Studies**	**Effect Estimate (95% CI)**	**Number of Studies**	**Effect Estimate (95% CI)**	**Number of Studies**	**Effect Estimate (95% CI)**
Bifidobacterium-breve	0		0		0		0		0	
Bifidobacterium-animalis-subsp.-lactis	6	0.13 (-0.05, 0.30)	6	0.10 (-0.01, 0.21)	5	0.04 (-0.10, 0.19)	2	0.01 (-0.27, 0.28)	1	0.16 (-0.02, 0.33)
Lactobacillus-fermentum	0		0		0		0		0	
Saccharomyces-boulardii	3	0.87 (-0.43, 2.16)	1	0.50 (0.10, 0.89)	1	0.56 (0.16, 0.96)	0		0	
Lactobacillus-reuteri	1	1.50 (1.04, 1.96)	1	0.76 (0.34, 1.18)	1	0.58 (0.17, 1.00)	0		0	
Bifidobacterium-longum-subsp.-infantis	0		0		0		0		0	
Lactobacillus rhamnosus	0		0		0		0		0	
Bacillus-coagulans	0		0		0		0		0	

Not specified	1	1.37 (0.77, 1.97)	1	0.39 (-0.15, 0.93)	0		0		0	
Lactobacillus-paracasei-ssp.-paracasei	1	0.00 (-0.20, 0.20)	1	0.07 (-0.12, 0.27)	1	0.00 (-0.20, 0.20)	0		0	
Lactobacillus-salivarius	0		0		0		0		0	
Lactobacillus-plantarum	2	0.33 (-0.36, 1.02)	0		0		0		0	
Bifidobacterium-longum	0		0		0		0		0	
Lactobacillus-sporogenes	1	0.06 (-0.20, 0.33)	0		0		0		0	
Lactobacillus-casei	0		0		0		0		0	
Enterococcus-faecium	1	0.25 (-0.19, 0.69)	0		0		0		0	
Lactobacillus-acidophilus	0		0		0		0		0	
Bifidobacterium-bifidum	0		0		0		0		0	
Lactobacillus-casei, Lactobacillus-reuteri	1	0.09 (-0.12, 0.31)	1	0.12 (-0.09, 0.34)	0		0		0	
Bifidobacterium-animalis-subsp.-lactis, Streptococcus-thermophilus	0		0		0		0		0	
Lactobacillus-rhamnosus, Bifidobacterium-infantis	2	-0.16 (-0.45, 0.13)	2	0.00 (-0.29, 0.29)	2	-0.34 (-0.71, 0.03)	0		0	
Bifidobacterium-breve, Streptococcus-thermophilus	0		0		0		0		0	
Lactobacillus-rhamnosus, Bifidobacterium-animalis-subsp.-lactis	0		0		0		0		0	
Lactobacillus-paracasei-ssp.-paracasei, Bifidobacterium-animalis-ssp.-lactis	0		0		0		0		0	
Bifidobacterium-animalis-subsp.-lactis, Bifidobacterium-longum	0		0		0		0		0	
Lactobacillus-paracasei, Bifidobacterium-longum	0		0		0		0		0	
Lactobacillus-acidophilus, Bifidobacterium-bifidum	1	0.26 (-0.25, 0.77)	0		0		0		0	
Bifidobacterium-spp., Lactobacillus-acidophilus	0		0		0		0		0	
Lactobacillus-fermentum, Bifidobacterium-breve	0		0		0		0		0	

Lactobacillus-reuteri, Bifidobacterium-animalis-subsp.-lactis	1	0.02 (-0.23, 0.27)	0		0		0		0	
Bifidobacterium-longum, Lactobacillus-rhamnosus	0		0		0		0		0	
Lactobacillus-rhamnosus, Bifidobacterium-longum-subsp.-infantis	0		0		0		0		0	
Bifidobacteria-infantis, Streptococcus-thermophilus, Bifidobacteria-bifidus	0		0		0		0		0	
Bifidobacterium-longum, Lactobacillus-rhamnosus, Lactobacillus-paracasei	0		0		0		0		0	
Bifidobacterium-infantis, Streptococcus-thermophilus, Bifidobacterium-animalis-subsp.-lactis	0		0		0		0		0	
Lactobacillus-rhamnosus, Bifidobacterium-breve, Propionibacterium-freudenreichii-ssp.-shermanii	0		0		0		0		0	
Bifidobacterium-infantis, Lactobacillus-helveticus, Bifidobacterium-bifidum	1	0.12 (-0.22, 0.46)	1	0.26 (-0.08, 0.60)	1	-0.16 (-0.50, 0.18)	1	-0.13 (-0.47, 0.21)	0	
Lactobacillus-rhamnosus, Lactobacillus-casei, Lactobacillus-plantarum, Bifidobacterium-animalis	0		0		0		0		0	
Lactobacillus-salivarius, Lactobacillus-paracasei, Bifidobacterium-animalis-subsp.-lactis, Bifidobacterium-bifidum	0		0		0		0		0	
Bifidobacterium-bifidum, Bifidobacterium-breve, Bifidobacterium-longum,	0		0		0		0		0	

Bifidobacterium-longum-subspecies-infantis										
Lactobacillus-rhamnosus, Lactobacillus-acidophilus, Bifidobacterium-longum, Bifidobacterium-bifidum, Bifidobacterium-animalis-subsp.-infantis	0		0		0		0		0	
Lactobacillus-acidophilus, Lactobacillus-rhamnosus, Lactobacillus-bulgaricus, Lactobacillus-casei, Bifidobacterium-infantis, Bifidobacterium-breve, Streptococcus-thermophilus	0		0		0		0		0	
Streptococcus-thermophilus, Bifidobacterium-breve, Bifidobacterium-longum-subsp.-longum, Bifidobacterium-longum-subsp.-infantis, Lactobacillus-acidophilus, Lactobacillus-plantarum, Lactobacillus-paracasei, Lactobacillus-delbrueckii-subsp.-bulgarius	0		0		0		0		0	
Lactobacillus-casei, Bifidobacterium-infantis, Lactobacillus-bulgaricus, Lactobacillus-acidophilus, Lactobacillus-rhamnosus, Streptococcus-thermophilus	1	-0.22 (-0.58, 0.14)	0		0		0		0	
Pediococcus-pentosaceus, Leuconostoc-mesenteroides, Lactobacillus-paracasei-ssp.-paracasei, Lactobacillus-plantarum	0		0		0		1	-0.05 (-0.26, 0.17)	1	0.16 (-0.02, 0.33)

Table S7. Pooled analysis based on probiotic strains: High income countries

		Weight		Height		Head Circumference		BMI		Weight for Length
**Probiotic**	**Number of Studies**	**Effect Estimate (95% CI)**	**Number of Studies**	**Effect Estimate (95% CI)**	**Number of Studies**	**Effect Estimate (95% CI)**	**Number of Studies**	**Effect Estimate (95% CI)**	**Number of Studies**	**Effect Estimate (95% CI)**
Bifidobacterium-breve	4	0.05 (-0.05, 0.14)	3	-0.02 (-0.22, 0.18)	3	'-0.57 (-2.06, 0.91)	0		0	
Bifidobacterium-animalis-subsp.-lactis	5	0.02 (-0.30, 0.35)	3	-0.06 (-0.34, 0.22)	2	0.18 (-0.40, 0.75)	0		0	
Lactobacillus-fermentum	3	0.02 (-0.15, 0.20)	3	-0.10 (-0.31, 0.11)	3	0.03 (-0.14, 0.21)	0		0	
Saccharomyces-boulardii	1	0.44 (0.01, 0.88)	0		0		0		0	
Lactobacillus-reuteri	4	-0.04 (-0.25, 0.17)	2	-0.04 (-0.43, 0.35)	3	0.05 (-0.16, 0.27)	1	0.00 (-0.50, 0.50)	1	0.14 [-0.20, 0.47]
Bifidobacterium-longum-subsp.-infantis	2	-0.02 (-0.28, 0.25)	1	0.01 (-0.31, 0.33)	1	0.06 (-0.26, 0.38)	1	0.01 (-0.31, 0.32)	0	
Lactobacillus rhamnosus	5	-0.02 (-0.20, 0.15)	3	-0.08 (-0.27, 0.11)	2	-0.03 (-0.34, 0.29)	1	-0.11 (-0.48, 0.26)	0	
Bacillus-coagulans	0		0		0		0		0	
Not specified	0		0		0		0		0	
Lactobacillus-paracasei-ssp.-paracasei	1	0.21 (-0.09, 0.51)	1	0.18 (-0.12, 0.48)	1	0.42 (0.12, 0.72)	1	0.19 (-0.11, 0.49)	0	
Lactobacillus-salivarius	1	0.35 (-0.09, 0.79)	1	0.15 (-0.29, 0.59)	1	0.40 (-0.05, 0.84)	0		0	
Lactobacillus-plantarum	0		0		0		0		0	
Bifidobacterium-longum	1	0.07 (-0.34, 0.47)	1	0.01 (-0.39, 0.42)	1	0.18 (-0.22, 0.58)	0		0	
Lactobacillus-sporogenes	0		0		0		0		0	
Lactobacillus-casei	0		0		0		0		0	
Enterococcus-faecium	0		0		0		0		0	
Lactobacillus-acidophilus	1	-0.09 (-0.38, 0.21)	1	-0.06 (-0.36, 0.24)	0		0		0	
Bifidobacterium-bifidum	1	-0.08 (-0.34, 0.17)	0		1	-0.15 (-0.41, 0.10)	0		0	
Lactobacillus-casei, Lactobacillus-reuteri	0		0		0		0		0	
Bifidobacterium-animalis-subsp.-lactis, Streptococcus-thermophilus	1	-0.02 (-0.40, 0.36)	1	0.10 (-0.28, 0.48)	0		0		1	-0.05 [-0.43, 0.33]
Lactobacillus-rhamnosus, Bifidobacterium-infantis	1	0.09 (-0.30, 0.49)	0		0		0		0	
Bifidobacterium-breve, Streptococcus-thermophilus	1	0.08 (-0.05, 0.21)	1	0.02 (-0.11, 0.15)	0		0		0	
Lactobacillus-rhamnosus, Bifidobacterium-animalis-subsp.-lactis	2	0.03 (-0.16, 0.22)	1	0.11 (-0.09, 0.30)	1	0.18 (-0.02, 0.37)	0		0	

Lactobacillus-paracasei-ssp.-paracasei, Bifidobacterium-animalis-ssp.-lactis	1	-0.09 (-0.53, 0.36)	1	0.01 (-0.43, 0.45)	1	-0.01 (-0.45, 0.43)	0		0	
Bifidobacterium-animalis-subsp.-lactis, Bifidobacterium-longum	1	-0.26 (-0.58, 0.06)	1	-0.11 (-0.43, 0.21)	1	-0.35 (-0.67, -0.03)	0		0	
Lactobacillus-paracasei, Bifidobacterium-longum	0		0		0		0		0	
Lactobacillus-acidophilus, Bifidobacterium-bifidum	1	-0.09 (-0.28, 0.10)	0		0		0		0	
Bifidobacterium-spp., Lactobacillus-acidophilus	0		0		0		0		0	
Lactobacillus-fermentum, Bifidobacterium-breve	1	-0.02 (-0.29, 0.25)	1	0.06 (-0.21, 0.33)	1	-0.27 (-0.54, 0.01)	0		0	
Lactobacillus-reuteri, Bifidobacterium-animalis-subsp.-lactis	2	-0.26 (-0.53, 0.01)	1	0.04 (-0.51, 0.58)	1	-0.50 (-1.06, 0.05)	0		0	
Bifidobacterium-longum, Lactobacillus-rhamnosus	2	-0.11 (-0.40, 0.18)	1	-0.16 (-0.51, 0.19)	1	-0.10 (-0.45, 0.24)	0		0	
Lactobacillus-rhamnosus, Bifidobacterium-longum-subsp.-infantis	1	0.42 (-0.01, 0.86)	1	0.26 (-0.17, 0.69)	1	0.41 (-0.02, 0.85)	0		0	
Bifidobacteria-infantis, Streptococcus-thermophilus, Bifidobacteria-bifidus	1	0.43 (0.10, 0.76)	0		0		0		0	
Bifidobacterium-longum, Lactobacillus-rhamnosus, Lactobacillus-paracasei	1	0.16 (-0.14, 0.47)	1	0.27 (-0.04, 0.57)	1	-0.02 (-0.33, 0.29)	1	0.25 (-0.05, 0.56)	0	
Bifidobacterium-infantis, Streptococcus-thermophilus, Bifidobacterium-animalis-subsp.-lactis	1	0.01 (-0.11, 0.13)	0		0		0		0	
Lactobacillus-rhamnosus, Bifidobacterium-breve, Propionibacterium-freudenreichii-ssp.-shermanii	1	0.00 (-0.13, 0.13)	1	-0.06 (-0.19, 0.07)	1	-0.06 (-0.19, 0.07)	0		0	
Bifidobacterium-infantis, Lactobacillus-	1	-0.18 (-0.50, 0.14)	1	-0.19 (-0.51, 0.13)	1	-0.02 (-0.34, 0.30)	0		0	

helveticus, Bifidobacterium-bifidum										
Lactobacillus-rhamnosus, Lactobacillus-casei, Lactobacillus-plantarum, Bifidobacterium-animalis	0		0		0		0		0	
Lactobacillus-salivarius, Lactobacillus-paracasei, Bifidobacterium-animalis-subsp.-lactis, Bifidobacterium-bifidum	1	-0.16 (-0.38, 0.06)	0		0		0		0	
Bifidobacterium-bifidum, Bifidobacterium-breve, Bifidobacterium-longum, Bifidobacterium-longum-subspecies-infantis	1	0.10 (-0.74, 0.94)	1	-0.08 (-0.48, 0.32)	0		0		0	
Lactobacillus-rhamnosus, Lactobacillus-acidophilus, Bifidobacterium-longum, Bifidobacterium-bifidum, Bifidobacterium-animalis-subsp.-infantis	1	0.03 (-0.54, 0.60)	0		0		0		0	
Lactobacillus-acidophilus, Lactobacillus-rhamnosus, Lactobacillus-bulgaricus, Lactobacillus-casei, Bifidobacterium-infantis, Bifidobacterium-breve, Streptococcus-thermophilus	0		0		0		0		0	
Streptococcus-thermophilus, Bifidobacterium-breve, Bifidobacterium-longum-subsp.-longum, Bifidobacterium-longum-subsp.-infantis, Lactobacillus-acidophilus, Lactobacillus-plantarum, Lactobacillus-paracasei,	0		0		0		0		0	

Lactobacillus-delbrueckii-subsp.-bulgarius										
Lactobacillus-casei, Bifidobacterium-infantis, Lactobacillus-bulgaricus, Lactobacillus-acidophilus, Lactobacillus-rhamnosus, Streptococcus-thermophilus	0		0		0		0		0	
Pediococcus-pentosaceus, Leuconostoc-mesenteroides, Lactobacillus-paracasei-ssp.-paracasei, Lactobacillus-plantarum	0		0		0		0		0	

Table S6 and S7 displays the growth outcomes and the probiotic strains represented in each outcome. We included the number of studies that used each strain for a particular outcome, and also the effect estimate with 95% CI. NA= not applicable.

Figure S1. Funnel Plot: Effect of probiotics on growth: Low- and middle-income countries: Weight

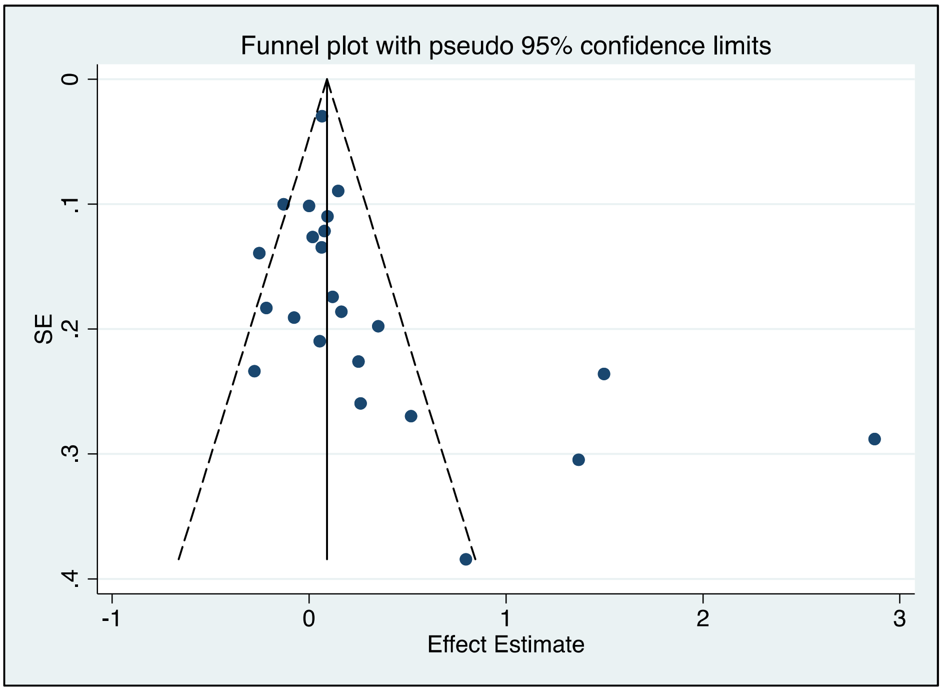

Figure S2. Funnel Plot: Effect of probiotics on growth: High income countries: Weight

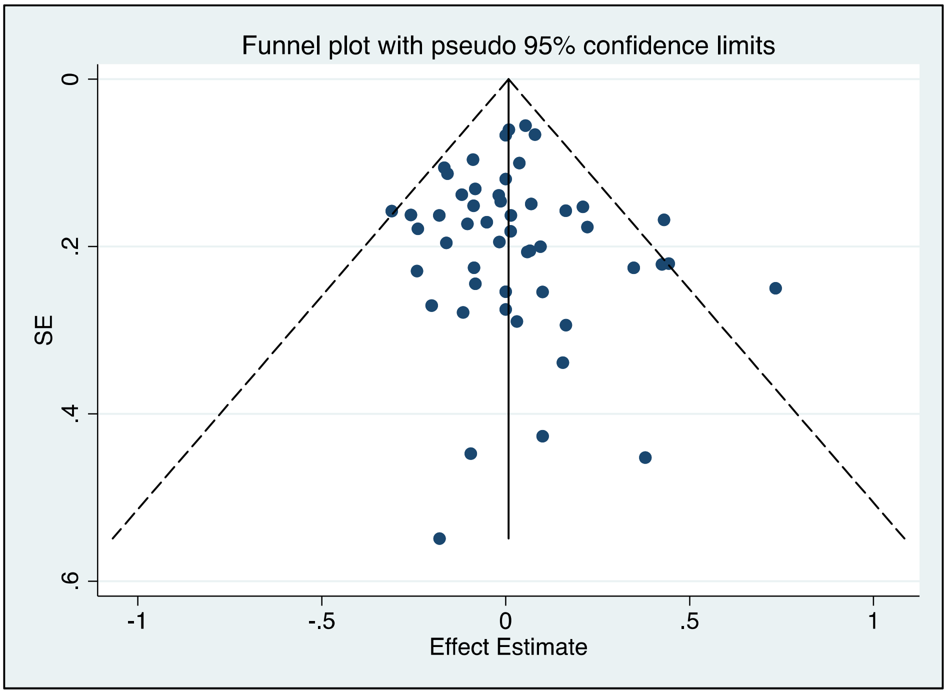

Figure S3. Funnel Plot: Effect of probiotics on growth: Low- and middle-income countries: Head Circumference

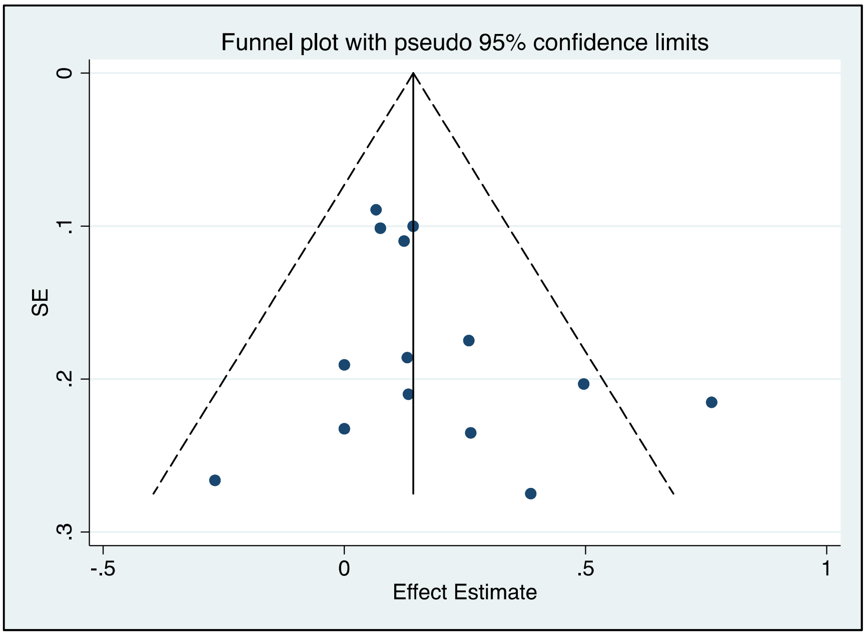

Figure S4. Funnel Plot: Effect of probiotics on growth: High income countries: Height

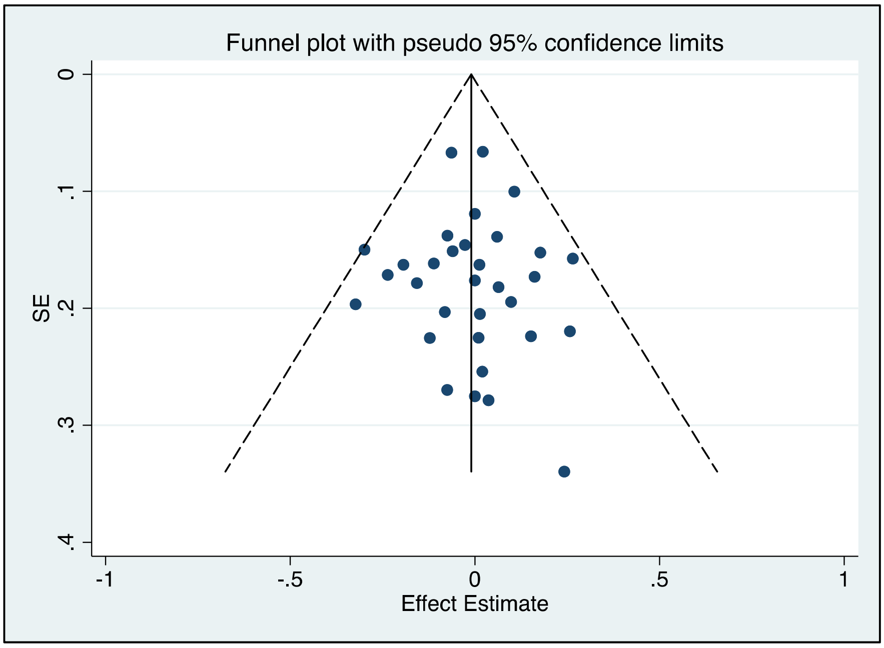

Figure S5. Funnel Plot: Effect of probiotics on growth: High income countries: Head circumference

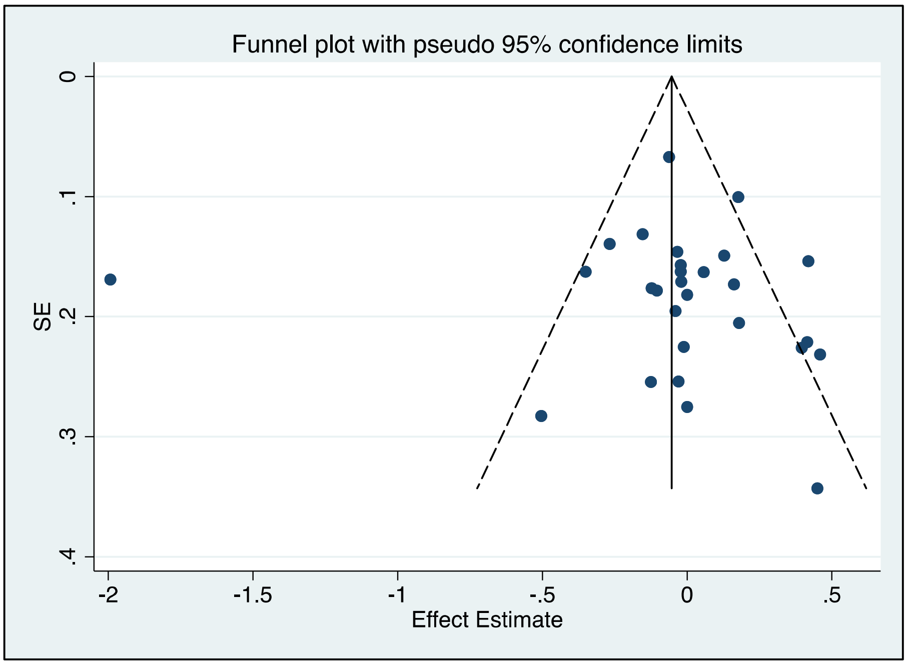

Figure S6. Risk of bias in the included studies from low- and middle-income countries for effect of probiotics on the outcome of weight for age

Study	Randomization process	Deviations from intended interventions	Missing outcome data	Measurement of the outcome	Selection of the reported result	Overall
Agustina 2013	+	+	+	+	+	+
Amini 2017	+	–	+	+	+	–
Batac 2005	+	+	+	+	+	+
Cooper 2016 C-Section	+	+	+	+	+	+
Cooper 2016 Vaginal Delivery	+	+	+	+	+	+
Cui 2019	+	+	+	+	+	+
Demirel 2013	+	+	+	+	+	+
Dilli 2015	+	+	+	+	+	+
Li 2019	+	+	+	+	+	+
Mitra 2014	+	–	+	+	+	–
Panigrahi 2008	?	+	+	+	+	!
Panigrahi 2017	+	+	+	+	+	+
Saengtawesin 2014	?	+	+	+	+	!
Sari 2010	+	+	+	+	+	!
Sazawal 2010	+	+	+	+	+	+
Serce 2013	+	+	+	+	+	+
Surono 2011	?	+	–	+	+	–
Urban 2008	+	+	+	+	+	+
Van Niekert 2014 HIV Exposed	+	+	+	+	+	+
Van Niekert 2014 HIV Unexposed	+	+	+	+	+	+
Velaphi 2008	?	+	+	+	+	!
Xiao 2019	+	+	+	+	+	+
Xu 2016	+	+	+	+	+	+

Figure S7. Risk of bias in the included studies from low- and middle-income countries for the outcome of height for age

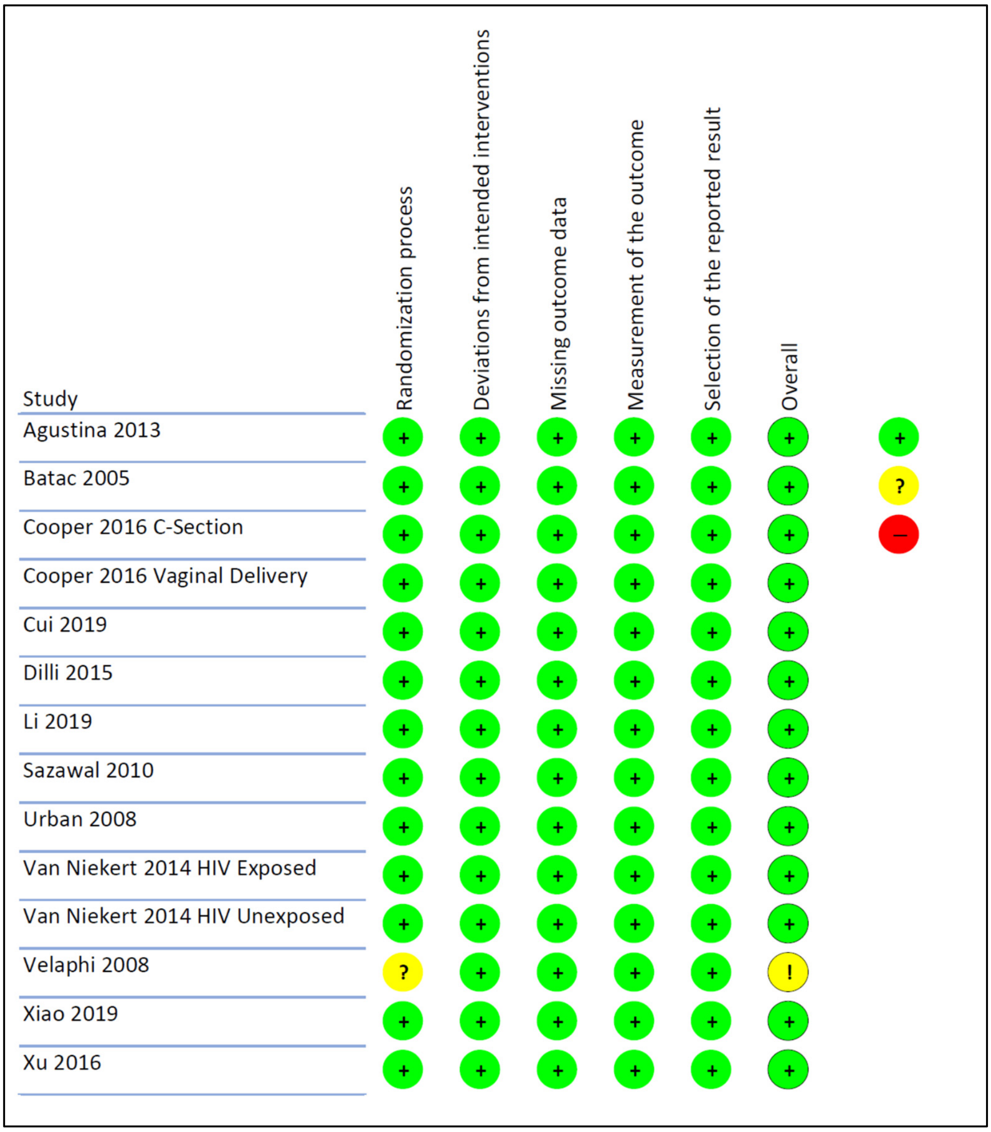

Figure S8. Risk of bias in the included studies from low- and middle-income countries for the outcome of head circumference

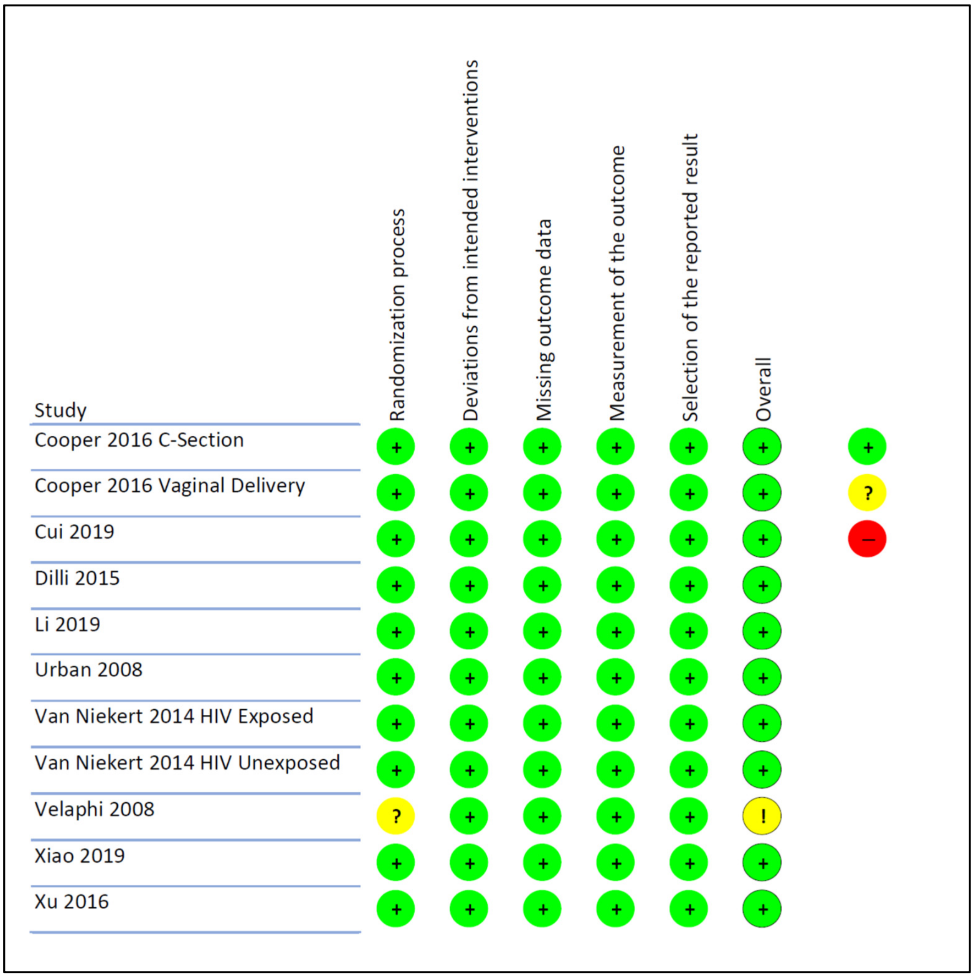

Figure S9. Risk of bias in the included studies from low- and middle-income countries for the outcome of sepsis

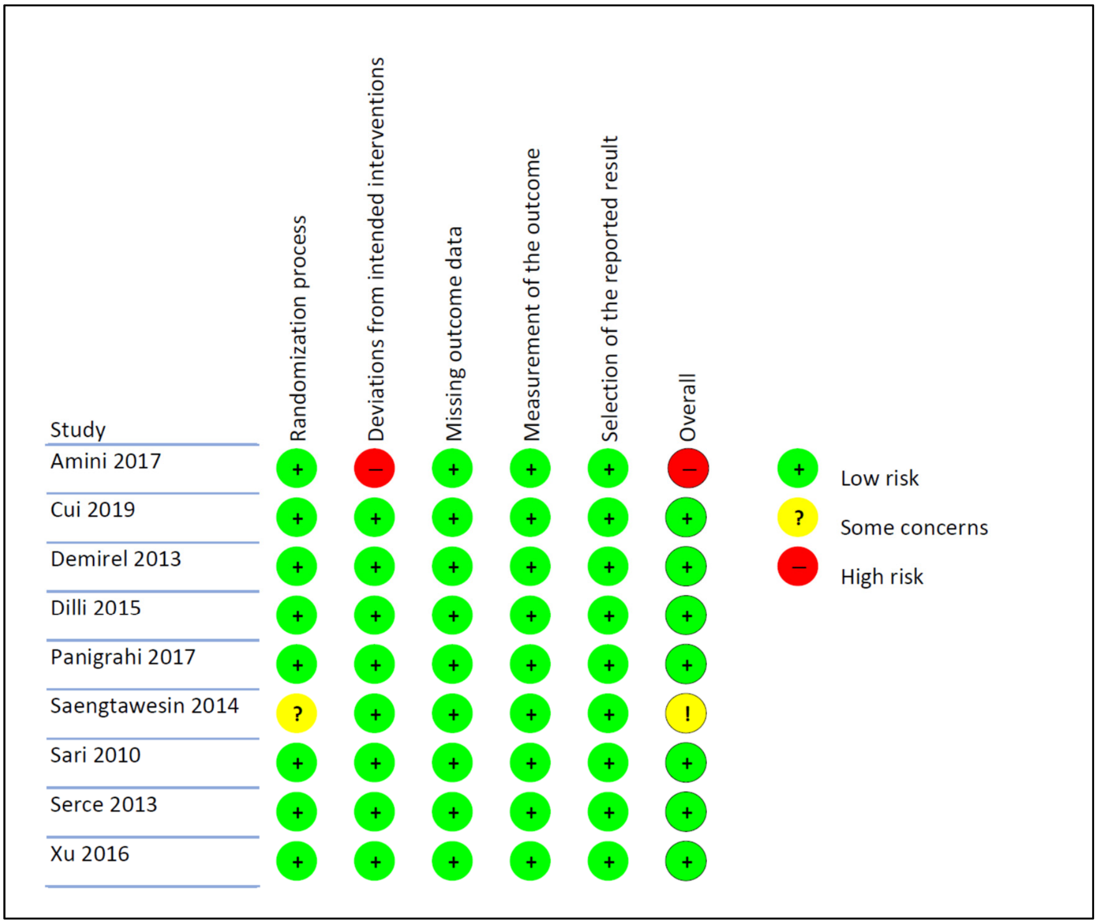

Figure S10. Forest Plot: Effect of use of probiotics on head circumference in children 0-59 months of age from low- and middle-income countries

Study or Subgroup	Std. Mean Difference	SE	Experimental Total	Control Total	Weight	Std. Mean Difference IV, Random, 95% CI
Cooper 2016 C-Section	0.1668	0.2101	45	46	8.1%	0.17 [-0.24, 0.58]
Cooper 2016 Vaginal Delivery	0.1617	0.1861	60	56	9.1%	0.16 [-0.20, 0.53]
Cui 2019	0.5849	0.2121	45	48	8.0%	0.58 [0.17, 1.00]
Dilli 2015	0	0.1	200	200	13.8%	0.00 [-0.20, 0.20]
Li 2019	0	0.1014	195	194	13.7%	0.00 [-0.20, 0.20]
Urban 2008	-0.0271	0.265	29	28	6.1%	-0.03 [-0.55, 0.49]
Van Niekerk 2014 HIV Exposed	-0.5578	0.2372	37	37	7.1%	-0.56 [-1.02, -0.09]
Van Niekerk 2014 HIV Unexposed	-0.1782	0.1911	54	56	8.9%	-0.18 [-0.55, 0.20]
Velaphi 2008	0	0.2341	37	36	7.2%	0.00 [-0.46, 0.46]
Xiao 2019	-0.1583	0.1744	66	66	9.7%	-0.16 [-0.50, 0.18]
Xu 2016	0.563	0.2041	51	49	8.3%	0.56 [0.16, 0.96]
**Total (95% CI)**			**819**	**816**	**100.0%**	**0.05 [-0.11, 0.21]**

Heterogeneity: $Tau^2 = 0.04$; $Chi^2 = 23.31$, df = 10 (P = 0.010); $I^2 = 57\%$
Test for overall effect: Z = 0.61 (P = 0.54)

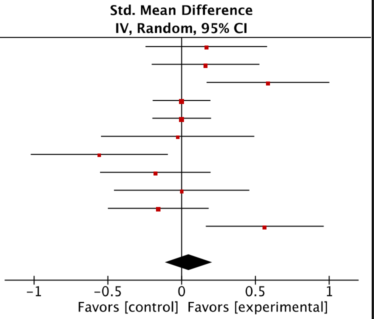

Figure S11. Forest Plot: Effect of use of probiotics on BMI in children 0-59 months of age from low- and middle-income countries

Study or Subgroup	Std. Mean Difference	SE	Experimental Total	Control Total	Weight	Std. Mean Difference IV, Random, 95% CI
Cooper 2016 C-Section	-0.0636	0.2097	45	46	26.9%	-0.06 [-0.47, 0.35]
Cooper 2016 Vaginal Delivery	0.0604	0.1858	60	56	34.2%	0.06 [-0.30, 0.42]
Xiao 2019	-0.1283	0.1743	66	66	38.9%	-0.13 [-0.47, 0.21]
**Total (95% CI)**			**171**	**168**	**100.0%**	**-0.05 [-0.26, 0.17]**

Heterogeneity: $Tau^2 = 0.00$; $Chi^2 = 0.56$, df = 2 (P = 0.76); $I^2 = 0\%$
Test for overall effect: Z = 0.43 (P = 0.67)

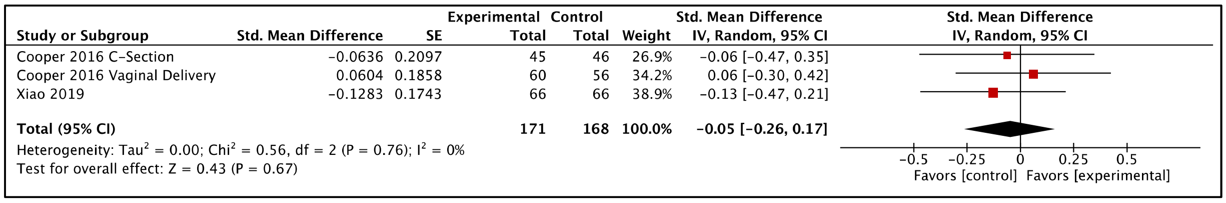

Figure S12. Forest Plot: Effect of use of probiotics on weight for height in children 0-59 months of age from low- and middle-income countries

Study or Subgroup	Std. Mean Difference	SE	Experimental Total	Control Total	Weight	Std. Mean Difference IV, Random, 95% CI
Sazawal 2010	0.1559	0.0894	257	245	100.0%	0.16 [−0.02, 0.33]
**Total (95% CI)**			**257**	**245**	**100.0%**	**0.16 [−0.02, 0.33]**

Heterogeneity: Not applicable
Test for overall effect: Z = 1.74 (P = 0.08)

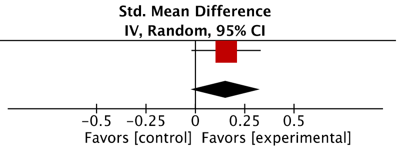

Figure S13. Forest Plot: Effect of use of probiotics on adverse events: Sepsis, in children 0-59 months of age from low- and middle-income countries

Study or Subgroup	Experimental Events	Experimental Total	Control Events	Control Total	Weight	Risk Ratio IV, Random, 95% CI
Amini 2017	1	60	0	60	0.2%	3.00 [0.12, 72.20]
Cui 2019	2	57	4	57	0.9%	0.50 [0.10, 2.62]
Demirel 2013	67	135	86	136	28.2%	0.78 [0.63, 0.97]
Dilli 2015	71	200	91	200	24.5%	0.78 [0.61, 0.99]
Panigrahi 2017	117	2278	202	2278	27.2%	0.58 [0.46, 0.72]
Saengtawesin 2014	2	31	1	29	0.4%	1.87 [0.18, 19.55]
Sari 2010	29	110	26	111	9.6%	1.13 [0.71, 1.78]
Serce 2013	19	104	25	104	7.4%	0.76 [0.45, 1.29]
Xu 2016	4	51	6	49	1.6%	0.64 [0.19, 2.13]
**Total (95% CI)**		**3026**		**3024**	**100.0%**	**0.74 [0.64, 0.87]**
Total events	312		441			

Heterogeneity: $Tau^2 = 0.01; Chi^2 = 10.04, df = 8 (P = 0.26); I^2 = 20\%$
Test for overall effect: Z = 3.73 (P = 0.0002)

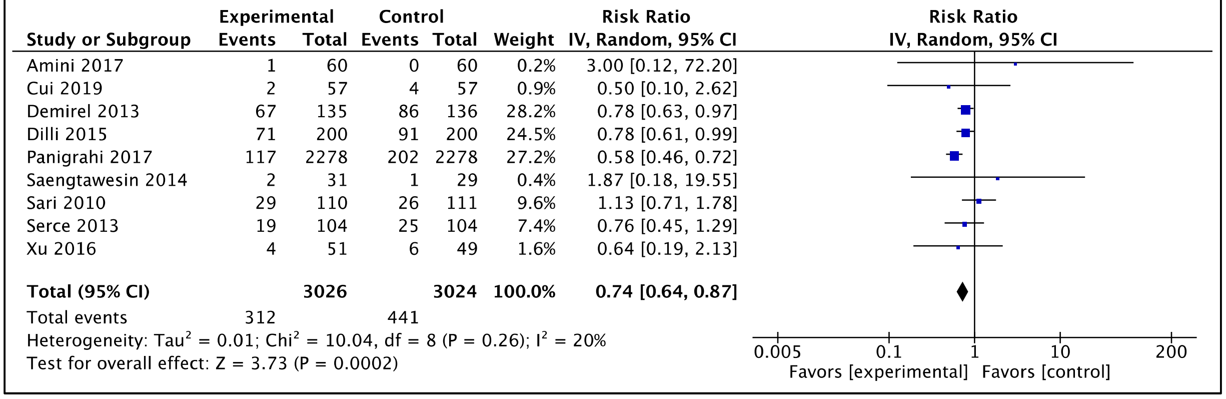

Figure S14. Forest Plot: Effect of use of probiotics on adverse events: Vomiting, in children 0-59 months of age from low- and middle-income countries

Study or Subgroup	Experimental Events	Experimental Total	Control Events	Control Total	Weight	Risk Ratio IV, Random, 95% CI
Li 2019	0	195	1	194	100.0%	0.33 [0.01, 8.09]
**Total (95% CI)**		**195**		**194**	**100.0%**	**0.33 [0.01, 8.09]**
Total events	0		1			

Heterogeneity: Not applicable
Test for overall effect: Z = 0.68 (P = 0.50)

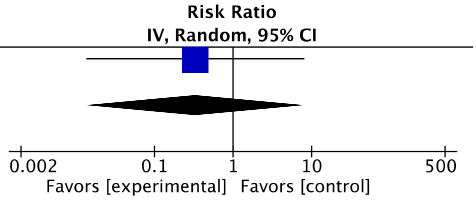

Figure S15. Forest Plot: Effect of use of probiotics on adverse events: Diarrhea, in children 0-59 months of age from low- and middle-income countries

Study or Subgroup	Experimental Events	Experimental Total	Control Events	Control Total	Weight	Risk Ratio IV, Random, 95% CI
Batac 2005	5	27	5	27	14.4%	1.00 [0.33, 3.06]
Cooper 2016 C-Section	0	92	0	101		Not estimable
Cooper 2016 Vaginal Delivery	0	115	0	113		Not estimable
Li 2019	51	195	43	194	27.9%	1.18 [0.83, 1.68]
Panigrahi 2008	0	19	4	12	3.7%	0.07 [0.00, 1.23]
Panigrahi 2017	12	2278	59	2278	23.0%	0.20 [0.11, 0.38]
Sur 2011	608	1802	674	1783	30.9%	0.89 [0.82, 0.97]
**Total (95% CI)**		**4528**		**4508**	**100.0%**	**0.64 [0.36, 1.14]**
Total events	676		785			

Heterogeneity: $Tau^2 = 0.28; Chi^2 = 27.36, df = 4 (P < 0.0001); I^2 = 85\%$
Test for overall effect: Z = 1.53 (P = 0.13)

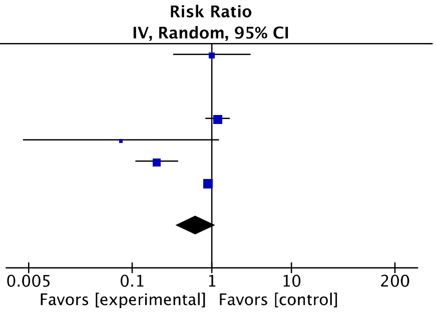

Figure S16. Forest Plot: Effect of use of probiotics on weight for age in children 0-59 months of age from high income countries

Study or Subgroup	Std. Mean Difference	SE	Experimental Total	Control Total	Weight	Std. Mean Difference IV, Random, 95% CI
Abrahamse-Berkeveld 2016	−0.1192	0.138	100	111	2.2%	−0.12 [−0.39, 0.15]
Ahrens 2018	0.0695	0.1491	90	90	1.9%	0.07 [−0.22, 0.36]
Al-Hosni 2011	0.0946	0.2001	50	50	1.1%	0.09 [−0.30, 0.49]
Allen 2010	−0.1583	0.1129	159	156	3.2%	−0.16 [−0.38, 0.06]
Aloisio 2018	0.2222	0.1767	71	59	1.4%	0.22 [−0.12, 0.57]
Bazanella 2017	0.1004	0.4267	48	49	0.2%	0.10 [−0.74, 0.94]
Bin-Nun 2005	0.4305	0.1681	72	73	1.5%	0.43 [0.10, 0.76]
Cekola 2015	−0.0513	0.1709	68	69	1.5%	−0.05 [−0.39, 0.28]
Chouraqui 2008	0.1634	0.1572	173	53	1.7%	0.16 [−0.14, 0.47]
Chrzanowska-Liszewska 2012	0.1638	0.2939	21	26	0.5%	0.16 [−0.41, 0.74]
Costalos 2003	0.4433	0.2204	51	36	0.9%	0.44 [0.01, 0.88]
Costeloe 2016	0.054	0.0554	648	657	9.8%	0.05 [−0.05, 0.16]
Dekker 2009	0.0373	0.1002	296	150	3.9%	0.04 [−0.16, 0.23]
Escribano 2018	0.0143	0.1628	73	78	1.6%	0.01 [−0.30, 0.33]
Gil-Campos 2012	0.0134	0.1818	61	60	1.3%	0.01 [−0.34, 0.37]
Hays 2015	−0.2576	0.1622	145	52	1.6%	−0.26 [−0.58, 0.06]
Hojsak 2010	0	0.1193	139	142	2.9%	0.00 [−0.23, 0.23]
Indrio 2008	0.3796	0.4522	10	10	0.2%	0.38 [−0.51, 1.27]
Jacobs 2013	0.0087	0.0603	548	551	8.8%	0.01 [−0.11, 0.13]
Kankaanpa 2002	−0.1797	0.5491	10	5	0.2%	−0.18 [−1.26, 0.90]
Kukkonen 2008	0	0.067	442	449	7.5%	0.00 [−0.13, 0.13]
Lin 2008	−0.0888	0.0961	217	217	4.2%	−0.09 [−0.28, 0.10]
Maldonado 2010	0.3481	0.2254	40	40	0.9%	0.35 [−0.09, 0.79]
Maldonado 2012	−0.0137	0.1459	97	91	2.0%	−0.01 [−0.30, 0.27]
Maldonado 2019	−0.0187	0.1388	159	77	2.2%	−0.02 [−0.29, 0.25]
Manzano 2017	−0.1804	0.1628	147	51	1.6%	−0.18 [−0.50, 0.14]
Meli 2014	−0.2386	0.1788	64	62	1.4%	−0.24 [−0.59, 0.11]
Millar 1993	−0.0948	0.4475	10	10	0.2%	−0.09 [−0.97, 0.78]
Mohan 2008	0.7338	0.2498	37	32	0.7%	0.73 [0.24, 1.22]
Oshiro 2019	0.1555	0.3388	17	18	0.4%	0.16 [−0.51, 0.82]
Papagaroufalis 2014	0	0.254	31	31	0.7%	0.00 [−0.50, 0.50]
Puccio 2007	0.0659	0.205	42	55	1.1%	0.07 [−0.34, 0.47]
Radke 2017	−0.1668	0.1057	179	180	3.6%	−0.17 [−0.37, 0.04]
Rouge 2009	0.0589	0.2065	45	49	1.0%	0.06 [−0.35, 0.46]
Roze 2012	0.425	0.2213	39	45	0.9%	0.42 [−0.01, 0.86]
Saavedra 2004	−0.0172	0.1945	78	40	1.2%	−0.02 [−0.40, 0.36]
Scalabrin 2009	0	0.2752	23	31	0.6%	0.00 [−0.54, 0.54]
Simeoni 2016	0.1002	0.2543	32	30	0.7%	0.10 [−0.40, 0.60]
Smilowitz 2017	−0.0824	0.2445	34	33	0.7%	−0.08 [−0.56, 0.40]
Stratiki 2007	−0.241	0.2293	41	36	0.8%	−0.24 [−0.69, 0.21]
Szajewska 2017	0.21	0.1525	85	88	1.8%	0.21 [−0.09, 0.51]
Taylor 2007	−0.0873	0.1513	88	87	1.9%	−0.09 [−0.38, 0.21]
Thibault 2004	0.0797	0.0662	464	449	7.7%	0.08 [−0.05, 0.21]
Totsu 2014	−0.0827	0.1311	119	114	2.4%	−0.08 [−0.34, 0.17]
Underwood 2009	0.0305	0.2896	32	19	0.5%	0.03 [−0.54, 0.60]
Vendt 2006	−0.1612	0.1956	51	54	1.2%	−0.16 [−0.54, 0.22]
Vlieger 2009	−0.086	0.2253	41	38	0.9%	−0.09 [−0.53, 0.36]
Weizman 2005	−0.3097	0.1576	136	58	1.7%	−0.31 [−0.62, −0.00]
Weizman 2006	−0.1156	0.2788	40	19	0.6%	−0.12 [−0.66, 0.43]
Wejryd 2019	−0.1039	0.1729	68	66	1.5%	−0.10 [−0.44, 0.23]
Ziegler 2003	−0.2012	0.2705	28	27	0.6%	−0.20 [−0.73, 0.33]
**Total (95% CI)**			**5759**	**5073**	**100.0%**	**0.01 [−0.04, 0.05]**

Heterogeneity: $Tau^2 = 0.00$; $Chi^2 = 53.94$, df = 50 (P = 0.33); $I^2 = 7\%$

Test for overall effect: Z = 0.28 (P = 0.78)

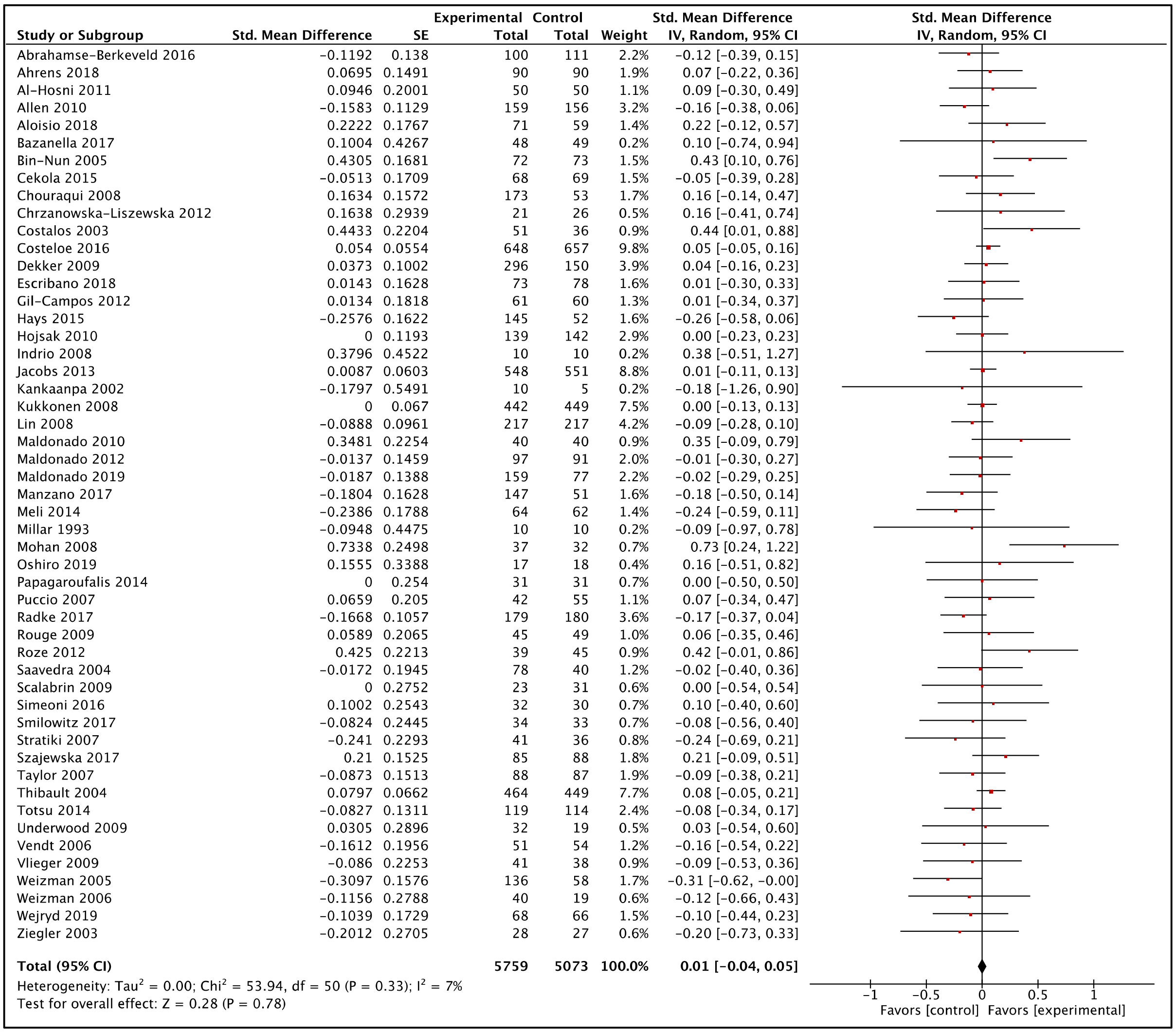

Figure S17. Forest Plot: Effect of use of probiotics on height for age in children 0-59 months of age from high income countries

Study or Subgroup	Std. Mean Difference	SE	Experimental Total	Control Total	Weight	Std. Mean Difference IV, Random, 95% CI
Abrahamse-Berkeveld 2016	−0.0743	0.1379	100	111	3.6%	−0.07 [−0.34, 0.20]
Ahrens 2018	−0.2988	0.1499	90	90	3.0%	−0.30 [−0.59, −0.01]
Aloisio 2018	0	0.1762	71	59	2.2%	0.00 [−0.35, 0.35]
Bazanella 2017	−0.0813	0.2032	48	49	1.7%	−0.08 [−0.48, 0.32]
Cekola 2015	−0.236	0.1715	68	69	2.3%	−0.24 [−0.57, 0.10]
Chouraqui 2008	0.2652	0.1575	173	53	2.8%	0.27 [−0.04, 0.57]
Dekker 2009	0.1068	0.1003	296	150	6.8%	0.11 [−0.09, 0.30]
Escribano 2018	0.0123	0.1628	73	78	2.6%	0.01 [−0.31, 0.33]
Gil-Campos 2012	0.0641	0.1819	61	60	2.1%	0.06 [−0.29, 0.42]
Hays 2015	−0.1112	0.1617	145	52	2.6%	−0.11 [−0.43, 0.21]
Hojsak 2010	0	0.1193	139	142	4.8%	0.00 [−0.23, 0.23]
Kukkonen 2008	−0.0634	0.067	442	449	15.2%	−0.06 [−0.19, 0.07]
Maldonado 2010	0.1519	0.2239	40	40	1.4%	0.15 [−0.29, 0.59]
Maldonado 2012	−0.0268	0.1459	97	91	3.2%	−0.03 [−0.31, 0.26]
Maldonado 2019	0.0601	0.1389	159	77	3.5%	0.06 [−0.21, 0.33]
Manzano 2017	−0.1936	0.1628	147	51	2.6%	−0.19 [−0.51, 0.13]
Meli 2014	−0.1569	0.1785	64	62	2.1%	−0.16 [−0.51, 0.19]
Oshiro 2019	0.2421	0.3396	17	18	0.6%	0.24 [−0.42, 0.91]
Puccio 2007	0.0136	0.2049	42	55	1.6%	0.01 [−0.39, 0.42]
Roze 2012	0.2574	0.2197	39	45	1.4%	0.26 [−0.17, 0.69]
Saavedra 2004	0.098	0.1946	78	40	1.8%	0.10 [−0.28, 0.48]
Scalabrin 2009	0	0.2752	23	31	0.9%	0.00 [−0.54, 0.54]
Simeoni 2016	0.0202	0.2541	32	30	1.1%	0.02 [−0.48, 0.52]
Stratiki 2007	−0.1219	0.2254	41	38	1.3%	−0.12 [−0.56, 0.32]
Szajewska 2017	0.1773	0.1524	85	88	2.9%	0.18 [−0.12, 0.48]
Taylor 2007	−0.0601	0.1512	88	87	3.0%	−0.06 [−0.36, 0.24]
Thibault 2004	0.0214	0.0662	464	449	15.6%	0.02 [−0.11, 0.15]
Vendt 2006	−0.3229	0.1966	51	54	1.8%	−0.32 [−0.71, 0.06]
Vlieger 2009	0.0101	0.2252	41	38	1.3%	0.01 [−0.43, 0.45]
Weizman 2006	0.037	0.2786	40	19	0.9%	0.04 [−0.51, 0.58]
Wejryd 2019	0.1617	0.1731	68	66	2.3%	0.16 [−0.18, 0.50]
Ziegler 2003	−0.0749	0.2698	28	27	0.9%	−0.07 [−0.60, 0.45]
**Total (95% CI)**			**3350**	**2768**	**100.0%**	**−0.01 [−0.06, 0.04]**

Heterogeneity: $Tau^2 = 0.00$; $Chi^2 = 22.23$, df = 31 (P = 0.88); $I^2 = 0\%$
Test for overall effect: Z = 0.38 (P = 0.71)

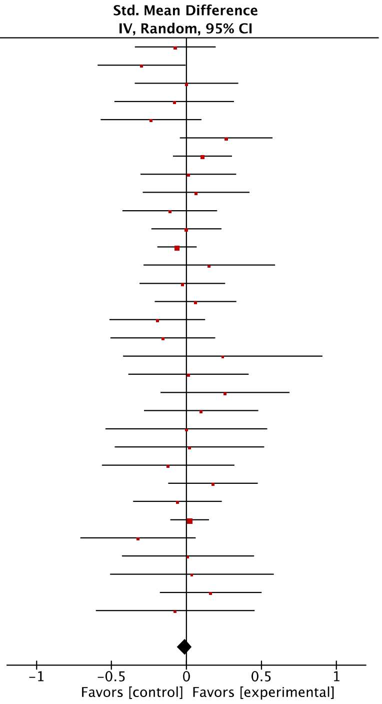

Figure S18. Forest Plot: Effect of use of probiotics on weight for length in children 0-59 months of age from high income countries

Study or Subgroup	Std. Mean Difference	SE	Experimental Total	Control Total	Weight	Std. Mean Difference IV, Random, 95% CI
Cekola 2015	0.1355	0.1711	68	69	56.4%	0.14 [−0.20, 0.47]
Saavedra 2004	−0.0476	0.1945	78	40	43.6%	−0.05 [−0.43, 0.33]
**Total (95% CI)**			**146**	**109**	**100.0%**	**0.06 [−0.20, 0.31]**

Heterogeneity: $Tau^2 = 0.00$; $Chi^2 = 0.50$, df = 1 (P = 0.48); $I^2 = 0\%$
Test for overall effect: Z = 0.43 (P = 0.67)

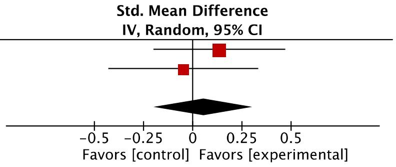

Figure S19. Forest Plot: Effect of use of probiotics on head circumference for age in children 0-59 months of age from high income countries

Study or Subgroup	Std. Mean Difference	SE	Experimental Total	Control Total	Weight	Std. Mean Difference IV, Random, 95% CI
Abrahamse-Berkeveld 2016	-1.9928	0.1691	100	111	3.7%	-1.99 [-2.32, -1.66]
Ahrens 2018	0.1279	0.1492	90	90	3.9%	0.13 [-0.16, 0.42]
Aloisio 2018	-0.1228	0.1763	71	59	3.7%	-0.12 [-0.47, 0.22]
Cekola 2015	-0.0203	0.1709	68	69	3.7%	-0.02 [-0.36, 0.31]
Chouraqui 2008	-0.0221	0.157	173	53	3.8%	-0.02 [-0.33, 0.29]
Dekker 2009	0.1767	0.1004	296	150	4.2%	0.18 [-0.02, 0.37]
Escribano 2018	0.057	0.1629	73	78	3.8%	0.06 [-0.26, 0.38]
Gil-Campos 2012	0	0.1818	61	60	3.6%	0.00 [-0.36, 0.36]
Hays 2015	-0.3507	0.1626	145	52	3.8%	-0.35 [-0.67, -0.03]
Kukkonen 2008	-0.0623	0.067	442	449	4.3%	-0.06 [-0.19, 0.07]
Maldonado 2010	0.3958	0.2259	40	40	3.3%	0.40 [-0.05, 0.84]
Maldonado 2012	-0.034	0.146	97	91	3.9%	-0.03 [-0.32, 0.25]
Maldonado 2019	-0.2677	0.1394	159	77	3.9%	-0.27 [-0.54, 0.01]
Manzano 2017	-0.0221	0.1625	147	51	3.8%	-0.02 [-0.34, 0.30]
Meli 2014	-0.1046	0.1783	64	62	3.7%	-0.10 [-0.45, 0.24]
Oshiro 2019	0.4498	0.343	17	18	2.5%	0.45 [-0.22, 1.12]
Papagaroufalis 2014	-0.0298	0.254	31	31	3.1%	-0.03 [-0.53, 0.47]
Puccio 2007	0.1792	0.2053	42	55	3.5%	0.18 [-0.22, 0.58]
Roze 2012	0.4147	0.2212	39	45	3.3%	0.41 [-0.02, 0.85]
Scalabrin 2009	0	0.2752	23	31	2.9%	0.00 [-0.54, 0.54]
Simeoni 2016	-0.1251	0.2544	32	30	3.1%	-0.13 [-0.62, 0.37]
Stratiki 2007	0.4593	0.2315	41	36	3.3%	0.46 [0.01, 0.91]
Szajewska 2017	0.4187	0.1538	85	88	3.8%	0.42 [0.12, 0.72]
Totsu 2014	-0.1537	0.1313	119	114	4.0%	-0.15 [-0.41, 0.10]
Vendt 2006	-0.0401	0.1953	51	54	3.5%	-0.04 [-0.42, 0.34]
Vlieger 2009	-0.0116	0.2252	41	38	3.3%	-0.01 [-0.45, 0.43]
Weizman 2006	-0.5041	0.2827	40	19	2.9%	-0.50 [-1.06, 0.05]
Wejryd 2019	0.1617	0.1731	68	66	3.7%	0.16 [-0.18, 0.50]
**Total (95% CI)**			**2655**	**2117**	**100.0%**	**-0.04 [-0.20, 0.11]**

Heterogeneity: $Tau^2 = 0.14$; $Chi^2 = 175.93$, df = 27 ($P < 0.00001$); $I^2 = 85\%$
Test for overall effect: Z = 0.55 (P = 0.58)

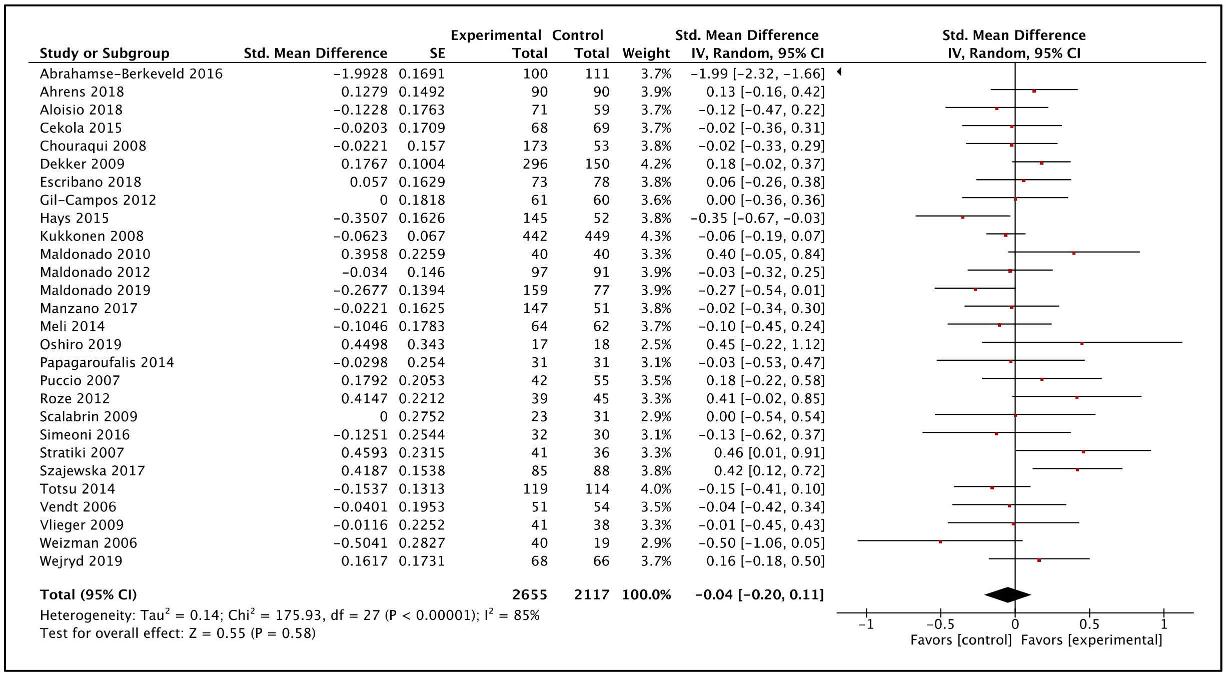

Figure S20. Forest Plot: Effect of use of probiotics on BMI in children 0-59 months of age from high income countries

Study or Subgroup	Std. Mean Difference	SE	Experimental Total	Control Total	Weight	Std. Mean Difference IV, Random, 95% CI
Chouraqui 2008	0.2544	0.1575	173	53	24.5%	0.25 [-0.05, 0.56]
Escribano 2018	0.0059	0.1628	73	78	22.9%	0.01 [-0.31, 0.32]
Luoto 2010	-0.1094	0.1894	53	55	16.9%	-0.11 [-0.48, 0.26]
Papagaroufalis 2014	0	0.254	31	31	9.4%	0.00 [-0.50, 0.50]
Szajewska 2017	0.1877	0.1524	85	88	26.2%	0.19 [-0.11, 0.49]
**Total (95% CI)**			**415**	**305**	**100.0%**	**0.09 [-0.06, 0.25]**

Heterogeneity: $Tau^2 = 0.00$; $Chi^2 = 3.00$, df = 4 (P = 0.56); $I^2 = 0\%$
Test for overall effect: Z = 1.21 (P = 0.23)

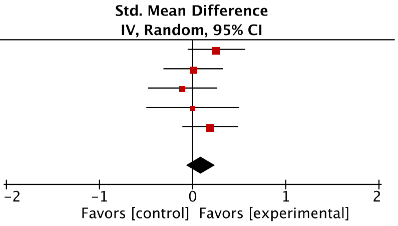

Figure S21. Forest Plot: Effect of use of probiotics on adverse events: Sepsis, in children 0-59 months of age from high income countries

Study or Subgroup	Experimental Events	Experimental Total	Control Events	Control Total	Weight	Risk Ratio IV, Random, 95% CI
Al-Hosni 2011	13	50	16	51	8.6%	0.83 [0.45, 1.54]
Bin-Nun 2005	31	72	24	73	14.8%	1.31 [0.86, 2.00]
Costalos 2003	3	51	3	36	1.7%	0.71 [0.15, 3.30]
Jacobs 2013	129	548	146	551	28.4%	0.89 [0.72, 1.09]
Kukkonen 2008	6	506	6	512	3.1%	1.01 [0.33, 3.12]
Lin 2008	40	217	24	217	12.9%	1.67 [1.04, 2.67]
Millar 1993	0	10	0	10		Not estimable
Oshiro 2019	0	17	3	18	0.5%	0.15 [0.01, 2.72]
Rouge 2009	15	45	13	49	8.6%	1.26 [0.67, 2.34]
Stratiki 2007	0	41	3	36	0.5%	0.13 [0.01, 2.36]
Totsu 2014	13	153	17	130	7.4%	0.65 [0.33, 1.29]
Wejryd 2019	25	68	23	66	13.5%	1.05 [0.67, 1.66]
**Total (95% CI)**		**1778**		**1749**	**100.0%**	**1.03 [0.84, 1.26]**
Total events	275		278			

Heterogeneity: $Tau^2 = 0.03$; $Chi^2 = 13.60$, df = 10 (P = 0.19); $I^2 = 26\%$
Test for overall effect: Z = 0.25 (P = 0.80)

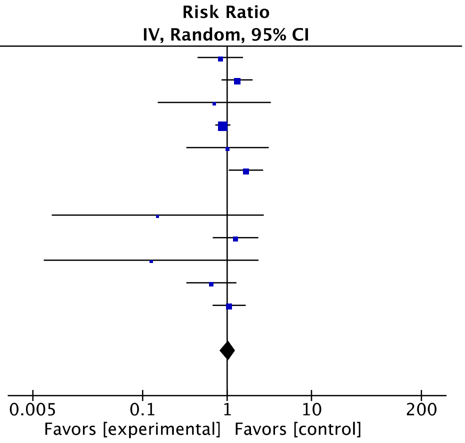

Figure S22. Forest Plot: Effect of use of probiotics on adverse events: Vomiting, in children 0-59 months of age from high income countries

Study or Subgroup	Experimental Events	Experimental Total	Control Events	Control Total	Weight	Risk Ratio IV, Random, 95% CI
Abrahamse-Berkeveld 2016	2	100	9	111	1.3%	0.25 [0.05, 1.11]
Aloisio 2018	0	82	0	73		Not estimable
Bin-Nun 2005	0	72	0	73		Not estimable
Cekola 2015	0	59	1	55	0.3%	0.31 [0.01, 7.48]
Chouraqui 2008	2	214	1	70	0.5%	0.65 [0.06, 7.11]
Costalos 2003	3	51	2	36	1.0%	1.06 [0.19, 6.02]
Dekker 2009	230	309	114	156	81.5%	1.02 [0.91, 1.14]
Harvey 2014	5	59	4	56	1.8%	1.19 [0.34, 4.19]
Hojsak 2010	10	139	17	142	5.0%	0.60 [0.29, 1.27]
Kukkonen 2008	4	506	7	512	1.9%	0.58 [0.17, 1.96]
Papagaroufalis 2014	1	44	5	44	0.6%	0.20 [0.02, 1.64]
Simeoni 2016	0	39	0	37		Not estimable
Szajewska 2017	10	90	12	92	4.5%	0.85 [0.39, 1.87]
Vlieger 2009	3	41	6	38	1.7%	0.46 [0.12, 1.72]
**Total (95% CI)**		**1805**		**1495**	**100.0%**	**0.93 [0.79, 1.10]**
Total events	270		178			

Heterogeneity: $Tau^2 = 0.01$; $Chi^2 = 10.28$, df = 10 (P = 0.42); $I^2 = 3\%$
Test for overall effect: Z = 0.82 (P = 0.41)

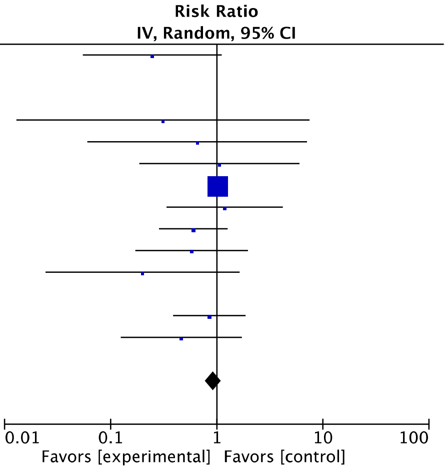

Figure S23. Forest Plot: Effect of use of probiotics on adverse events: Diarrhea, in children 0-59 months of age from high income countries

Study or Subgroup	Experimental Events	Experimental Total	Control Events	Control Total	Weight	Risk Ratio IV, Random, 95% CI
Abrahamse-Berkeveld 2016	2	100	1	111	0.6%	2.22 [0.20, 24.11]
Allen 2010	86	220	76	234	13.7%	1.20 [0.94, 1.54]
Bin-Nun 2005	0	72	0	73		Not estimable
Chouraqui 2008	3	214	1	70	0.7%	0.98 [0.10, 9.28]
Dekker 2009	235	309	117	156	17.0%	1.01 [0.91, 1.13]
Escribano 2018	3	93	7	97	1.8%	0.45 [0.12, 1.68]
Harvey 2014	3	59	2	56	1.1%	1.42 [0.25, 8.20]
Hojsak 2010	16	139	26	142	6.6%	0.63 [0.35, 1.12]
Maldonado 2010	7	40	26	40	5.0%	0.27 [0.13, 0.55]
Maldonado 2019	74	128	42	61	14.3%	0.84 [0.67, 1.05]
Manzano 2017	48	1350	14	468	6.5%	1.19 [0.66, 2.14]
Meli 2014	0	98	1	99	0.3%	0.34 [0.01, 8.17]
Radke 2017	43	179	31	180	9.6%	1.39 [0.92, 2.11]
Thibault 2004	263	464	251	449	16.9%	1.01 [0.90, 1.14]
Vendt 2006	2	51	0	54	0.4%	5.29 [0.26, 107.57]
Vlieger 2009	2	41	0	38	0.4%	4.64 [0.23, 93.71]
Weizman 2005	11	141	19	60	5.3%	0.25 [0.13, 0.49]
**Total (95% CI)**		**3698**		**2388**	**100.0%**	**0.89 [0.74, 1.07]**
Total events	798		614			

Heterogeneity: $Tau^2 = 0.05$; $Chi^2 = 43.90$, df = 15 (P = 0.0001); $I^2 = 66\%$
Test for overall effect: Z = 1.26 (P = 0.21)

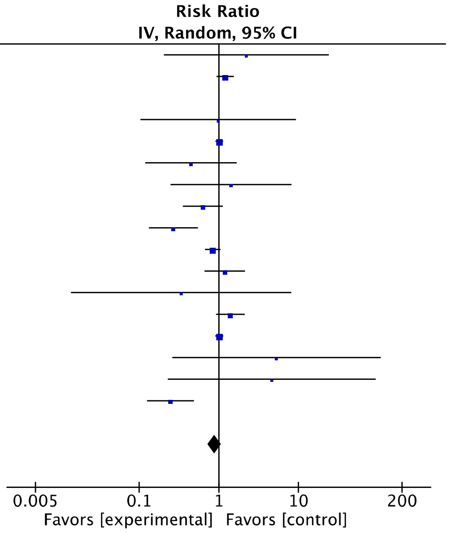

Figure S24. Forest Plot: Effect of use of probiotics on adverse events: Abdominal pain, in children 0-59 months of age from high income countries

Study or Subgroup	Experimental Events	Experimental Total	Control Events	Control Total	Weight	Risk Ratio IV, Random, 95% CI
Abrahamse-Berkeveld 2016	1	100	2	111	0.6%	0.56 [0.05, 6.03]
Aloisio 2018	0	82	0	73		Not estimable
Dekker 2009	156	309	77	156	98.7%	1.02 [0.84, 1.24]
Harvey 2014	2	59	1	56	0.7%	1.90 [0.18, 20.36]
Meli 2014	0	98	0	99		Not estimable
**Total (95% CI)**		**648**		**495**	**100.0%**	**1.02 [0.84, 1.24]**
Total events	159		80			

Heterogeneity: $Tau^2 = 0.00$; $Chi^2 = 0.51$, df = 2 (P = 0.77); $I^2 = 0\%$
Test for overall effect: Z = 0.23 (P = 0.82)

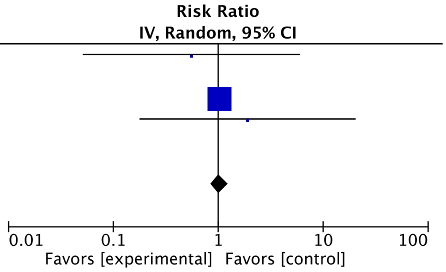

Figure S25. Forest Plot: Effect of use of probiotics on adverse events: Flatulence, in children 0-59 months of age from high income countries

Study or Subgroup	Experimental Events	Experimental Total	Control Events	Control Total	Weight	Risk Ratio IV, Random, 95% CI
Cekola 2015	4	59	2	55	4.7%	1.86 [0.36, 9.78]
Escribano 2018	26	93	33	95	70.3%	0.80 [0.53, 1.23]
Harvey 2014	13	59	11	56	25.1%	1.12 [0.55, 2.29]
Simeoni 2016	0	39	0	37		Not estimable
**Total (95% CI)**		**250**		**243**	**100.0%**	**0.91 [0.64, 1.30]**
Total events	43		46			

Heterogeneity: $Tau^2 = 0.00$; $Chi^2 = 1.37$, df = 2 (P = 0.51); $I^2 = 0\%$
Test for overall effect: Z = 0.52 (P = 0.60)

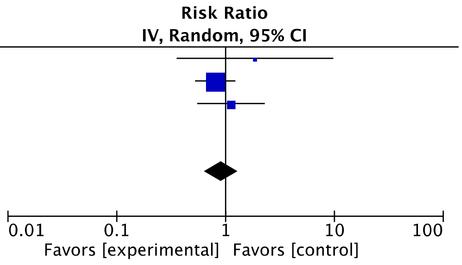

Figure S26. Risk of bias in the included studies from high income countries for effect of probiotics on the outcome of weight for age

Study	Randomization process	Deviations from intended interventions	Missing outcome data	Measurement of the outcome	Selection of the reported result	Overall
Abrahamse-Berkeveld 2016	+	+	+	+	+	+
Ahrens 2018	+	+	+	+	+	+
Al-Hosni 2012	+	+	+	+	+	+
Allen 2010	+	+	+	+	?	!
Aloisio 2018	+	+	+	+	+	+
Bazanella 2017	+	+	+	+	+	+
Bin-Nun 2005	+	+	+	+	+	+
Cekola 2015	+	+	+	+	+	+
Chouraqui 2008	+	−	+	+	+	−
Chrazanowska-Liszewska 2012	+	+	+	+	+	+
Costalos 2003	+	−	+	+	+	−
Costeloe 2016	+	+	+	+	+	+
Dekker 2009	+	+	+	+	+	+
Escribano 2018	+	+	+	+	+	+
Gil-Campos 2012	+	?	+	+	+	!
Hays 2015	+	+	+	+	+	+
Hojsak 2010	+	+	+	+	+	+
Indrio 2008	+	+	+	+	?	!
Jacobs 2013	+	+	+	+	+	+
Kankaanpa¨a¨ 2002	+	+	+	+	+	+
Kukkonen 2008	+	+	+	+	+	+
Lin 2008	?	+	+	+	+	!
Maldonado 2010	+	+	+	+	+	+
Maldonado 2012	?	−	+	+	+	−
Maldonado 2019	+	+	+	+	+	+
Manzano 2017	+	+	+	+	+	+
Meli 2014	+	+	+	+	+	+
Millar 1993	?	+	+	+	+	!
Mohan 2008	+	+	+	+	+	+
Oshiro 2019	?	+	+	+	+	!
Paparagoufalis 2014	+	+	+	+	+	+
Puccio 2007	?	+	+	+	+	!
Radke 2017	+	+	+	+	+	+
Rouge 2009	+	+	+	+	+	+
Roze 2012	+	+	+	+	+	+
Saavedra 2004	+	+	+	+	+	+
Scalabrin 2009	+	+	+	+	+	+
Simeoni 2016	?	+	+	+	?	!
Smilowitz 2017	+	+	+	+	+	+
Stratiki 2007	+	+	+	+	+	+
Szajewska 2017	+	+	+	+	+	+
Taylor 2007	?	+	+	+	+	!
Thibault 2004	+	?	+	+	+	!
Totsu 2014	?	+	+	+	+	!
Underwood 2009	+	+	+	+	+	+
Vendt 2006	−	+	+	+	+	−
Vlieger 2009	+	+	+	+	+	+
Weizman 2005	+	+	+	+	+	+
Weizman 2006	+	+	+	+	+	+
Wejryd 2019	+	+	+	+	+	+
Zeigler 2003	?	−	+	+	−	−

+ Low risk
? Some concerns
− High risk

Figure S27: Risk of bias in the included studies from high income countries for effect of probiotics on the outcome of height for age

Study	Randomization process	Deviations from intended interventions	Missing outcome data	Measurement of the outcome	Selection of the reported result	Overall
Abrahamse-Berkeveld 2016	+	+	+	+	+	+
Ahrens 2018	+	+	+	+	+	+
Aloisio 2018	+	+	+	+	+	+
Bazanella 2017	+	+	+	+	+	+
Cekola 2015	+	+	+	+	+	+
Chouraqui 2008	+	–	+	+	+	–
Dekker 2009	+	+	+	+	+	+
Escribano 2018	+	+	+	+	+	+
Gil-Campos 2012	+	?	+	+	+	!
Hays 2015	+	+	+	+	+	+
Hojsak 2010	+	+	+	+	+	+
Kukkonen 2008	+	+	+	+	+	+
Maldonado 2010	+	+	+	+	+	+
Maldonado 2012	?	–	+	+	+	–
Maldonado 2019	+	+	+	+	+	+
Manzano 2017	+	+	+	+	+	+
Meli 2014	+	+	+	+	+	+
Oshiro 2019	?	+	+	+	+	!
Puccio 2007	?	+	+	+	+	!
Roze 2012	+	+	+	+	+	+
Saavedra 2004	+	+	+	+	+	+
Scalabrin 2009	+	+	+	+	+	+
Simeoni 2016	?	+	+	+	?	!
Stratiki 2007	+	+	+	+	+	+
Szajewska 2017	+	+	+	+	+	+
Taylor 2007	?	+	+	+	+	!
Thibault 2004	+	?	+	+	+	!
Vendt 2006	–	+	+	+	+	–
Vlieger 2009	+	+	+	+	+	+
Weizman 2006	+	+	+	+	+	+
Wejryd 2019	+	+	+	+	+	+
Zeigler 2003	?	–	+	+	–	–

+ Low risk
? Some concerns
– High risk

Figure S28: Risk of bias in the included studies from high income countries for effect of probiotics on the outcome of weight for length

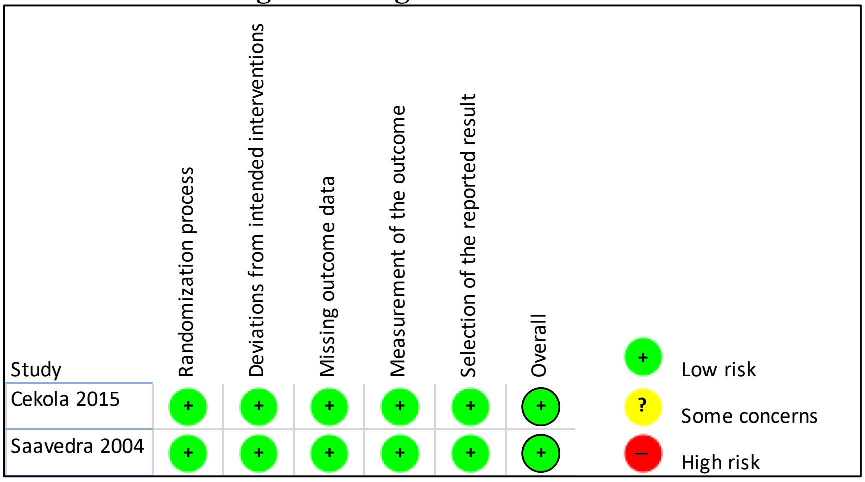

Figure S29: Risk of bias in the included studies from high income countries for effect of probiotics on the outcome of head circumference

Study	Randomization process	Deviations from intended interventions	Missing outcome data	Measurement of the outcome	Selection of the reported result	Overall
Abrahamse-Berkeveld 2016	+	+	+	+	+	+
Ahrens 2018	+	+	+	+	+	+
Aloisio 2018	+	+	+	+	+	+
Cekola 2015	+	+	+	+	+	+
Chouraqui 2008	+	–	+	+	+	–
Dekker 2009	+	+	+	+	+	+
Escribano 2018	+	+	+	+	+	+
Gil-Campos 2012	+	?	+	+	+	!
Hays 2015	+	+	+	+	+	+
Kukkonen 2008	+	+	+	+	+	+
Maldonado 2010	+	+	+	+	+	+
Maldonado 2012	?	–	+	+	+	–
Maldonado 2019	+	+	+	+	+	+
Manzano 2017	+	+	+	+	+	+
Meli 2014	+	+	+	+	+	+
Oshiro 2019	?	+	+	+	+	!
Paparagoufalis 2014	+	+	+	+	+	+
Puccio 2007	?	+	+	+	+	!
Roze 2012	+	+	+	+	+	+
Scalabrin 2009	+	+	+	+	+	+
Simeoni 2016	?	+	+	+	?	!
Stratiki 2007	+	+	+	+	+	+
Szajewska 2017	+	+	+	+	+	+
Totsu 2014	?	+	+	+	+	!
Vendt 2006	–	+	+	+	+	–
Vlieger 2009	+	+	+	+	+	+
Weizman 2006	+	+	+	+	+	+
Wejryd 2019	+	+	+	+	+	+

+ Low risk
? Some concerns
– High risk

Figure S30: Risk of bias in the included studies from high income countries for effect of probiotics on the outcome of BMI

Study	Randomization process	Deviations from intended interventions	Missing outcome data	Measurement of the outcome	Selection of the reported result	Overall
Chouraqui 2008	+	Ñ	+	+	+	Ñ
Escribano 2018	+	+	+	+	+	+
Luoto 2010	+	+	+	+	+	+
Paparagoufalis 2014	+	+	+	+	+	+
Szajewska 2017	+	+	+	+	+	+

+ Low risk
? Some concerns
Ñ High risk

Figure S31: Risk of bias in the included studies from high income countries for effect of probiotics on the outcome of sepsis

Study	Randomization process	Deviations from intended interventions	Missing outcome data	Measurement of the outcome	Selection of the reported result	Overall
Al-Hosni 2012	+	+	+	+	+	+
Bin-Nun 2005	+	+	+	+	+	+
Costalos 2003	+	–	+	+	+	–
Jacobs 2013	+	+	+	+	+	+
Kukkonen 2008	+	+	+	+	+	+
Lin 2008	?	+	+	+	+	!
Millar 1993	?	+	+	+	+	!
Oshiro 2019	?	+	+	+	+	!
Rouge 2009	+	+	+	+	+	+
Stratiki 2007	+	+	+	+	+	+
Totsu 2014	?	+	+	+	+	!
Wejryd 2019	+	+	+	+	+	+

+ Low risk
? Some concerns
– High risk

Figure S32: Subgroup and Sensitivity analyses in high income countries

	Number of Studies	Probiotics	Control	Total	I^2	Effect Estimate (95% CI)
**Primary Analyses**						
Weight	51	5759	5073	10832	7.30	0.01 (−0.04–0.05)
Height	32	3350	2768	6118	0	−0.01 (−0.06–0.04)
Weight–for–length	2	146	109	255	0	0.06 (−0.2–0.31)
Head Circumference	28	2655	2117	4772	84.65	−0.04 (−0.2–0.11)
BMI	5	415	305	720	0	0.09 (−0.06–0.25)
**Secondary Analyses**						
**Probiotics vs. Synbiotics**						
Weight						
Probiotics	39	4440	3853	8293	8.62	0.01 (−0.04–0.06)
Synbiotics	12	1319	1220	2539	10.67	0 (−0.08–0.09)
Height						
Probiotics	22	2242	1747	3989	0	0 (−0.07–0.06)
Synbiotics	10	1108	1021	2129	19.91	−0.01 (−0.12–0.1)
Weight–for–length						
Probiotics	2	146	109	255	0	0.06 (−0.2–0.31)
Synbiotics	0	0	0	0	0	0 (0–0)
Head Circumference						
Probiotics	18	1547	1096	2643	35.32	−0.01 (−0.11–0.09)
Synbiotics	10	1108	1021	2129	93.88	−0.12 (−0.51–0.27)
BMI						
Probiotics	4	242	252	494	0	0.04 (−0.13–0.22)
Synbiotics	1	173	53	226	0	0.25 (−0.05–0.56)
**Single vs. Multiple Strains**						
Weight						
Single Strain	28	2283	2302	4585	0	0.02 (−0.04–0.07)
Multiple Strains	23	3476	2771	6247	20.13	0 (−0.07–0.06)
Height						
Single Strain	18	1143	1175	2318	0	−0.03 (−0.12–0.05)
Multiple Strain	14	2207	1593	3800	0	0.01 (−0.06–0.07)
Weight–for–length						
Single Strain	1	68	69	137	0	0.14 (−0.2–0.47)
Multiple Strains	1	78	40	118	0	−0.05 (−0.43–0.33)
Head Circumference						
Single Strain	17	1038	1062	2100	89.85	−0.02 (−0.3–0.26)
Multiple Strains	11	1617	1055	2672	45.47	−0.06 (−0.18–0.06)
BMI						
Single Strain	4	242	252	494	0	0.04 (−0.13–0.22)
Multiple Strains	1	173	53	226	0	0.25 (−0.05–0.56)
**Nutritional Status**						
Weight						
Healthy	35	3638	3067	6705	0	−0.01 (−0.06–0.04)
Premature/LBW	16	2121	2006	4127	40.94	0.04 (−0.05–0.14)
Height						
Healthy	28	3079	2594	5673	0	−0.01 (−0.06–0.04)
Premature/LBW	4	271	174	445	0	0.01 (−0.19–0.2)
Weight–for–length						
Healthy	2	146	109	255	0	0.06 (−0.2–0.31)
Premature/LBW	0	0	0	0	0	0 (0–0)
Head Circumference						
Healthy	23	2265	1831	4096	86.54	−0.07 (−0.25–0.11)
Premature/LBW	5	390	286	676	67.81	0.05 (−0.24–0.35)
BMI						
Healthy	5	415	305	720	0	0.09 (−0.06–0.25)
Premature/LBW	0	0	0	0	0	0 (0–0)
**Age**						
Weight						
0–<6 Months	46	5162	4691	9853	6.82	0.02 (−0.03–0.06)
6–<60 Months	5	597	382	979	0	−0.09 (−0.22–0.04)
Height						
0–<6 Months	28	2889	2444	5333	0	−0.01 (−0.06–0.05)
6–<60 Months	4	461	324	785	0	−0.03 (−0.18–0.11)
Weight–for–length						
0–<6 Months	1	68	69	137	0	0.14 (−0.2–0.47)
6–<60 Months	1	78	40	118	0	−0.05 (−0.43–0.33)
Head Circumference						
0–<6 Months	26	2411	1975	4386	85.79	−0.05 (−0.22–0.13)
6–<60 Months	2	244	142	386	0	−0.03 (−0.24–0.18)
BMI						
0–<6 Months	5	415	305	720	0	0.09 (−0.06–0.25)
6–<60 Months	0	0	0	0	0	0 (0–0)
**Risk–of–Bias Sensitivity**						
Weight	46	5359	4812	10171	5.60	0 (−0.04–0.05)
Height	28	3001	2543	5544	0	−0.01 (−0.06–0.04)
Weight–for–length	2	146	109	255	0	0.06 (−0.2–0.31)
Head Circumference	25	2334	1919	4253	86.35	−0.05 (−0.22–0.13)
BMI	4	242	252	494	0	0.04 (−0.13–0.22)
**Graph–Data Sensitivity**						
Weight	45	5463	4761	10224	17.37	0.01 (−0.04–0.05)
Height	29	3161	2575	5736	0	−0.01 (−0.07–0.04)
Weight–for–length	2	146	109	255	0	0.06 (−0.2–0.31)
Head Circumference	25	2483	1942	4425	86.19	−0.06 (−0.23–0.12)
BMI	3	311	196	507	15.21	0.13 (−0.07–0.34)

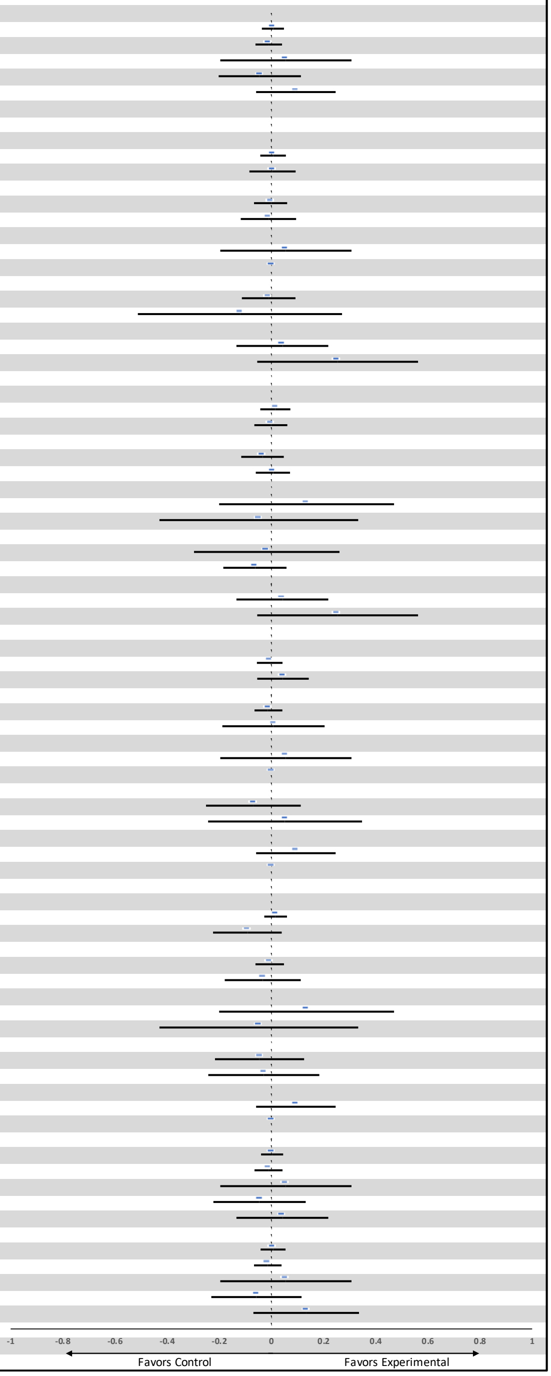

References

1. Cooper, P.; Bolton, K.D.; Velaphi, S.; de Groot, N.; Emady-Azar, S.; Pecquet, S.; Steenhout, P. Early Benefits of a Starter Formula Enriched in Prebiotics and Probiotics on the Gut Microbiota of Healthy Infants Born to HIV+ Mothers: A Randomized Double-Blind Controlled Trial. *Clin Med Insights Pediatr* **2016**, *10*, 119-130, doi:10.4137/CMPed.S40134.
2. Van Niekerk, E.; Kirsten, G.F.; Nel, D.G.; Blaauw, R. Probiotics, feeding tolerance, and growth: a comparison between HIV-exposed and unexposed very low birth weight infants. *Nutrition* **2014**, *30*, 645-653, doi:10.1016/j.nut.2013.10.024.
3. Wan, X.; Wang, W.; Liu, J.; Tong, T. Estimating the sample mean and standard deviation from the sample size, median, range and/or interquartile range. *BMC Medical Research Methodology* **2014**, *14*, 135, doi:10.1186/1471-2288-14-135.
4. Al-Hosni, M.; Duenas, M.; Hawk, M.; Stewart, L.A.; Borghese, R.A.; Cahoon, M.; Atwood, L.; Howard, D.; Ferrelli, K.; Soll, R. Probiotics-supplemented feeding in extremely low-birth-weight infants. *J Perinatol* **2012**, *32*, 253-259, doi:10.1038/jp.2011.51.
5. Cekola, P.L.; Czerkies, L.A.; Storm, H.M.; Wang, M.H.; Roberts, J.; Saavedra, J.M. Growth and Tolerance of Term Infants Fed Formula With Probiotic Lactobacillus reuteri. *Clin Pediatr (Phila)* **2015**, *54*, 1175-1184, doi:10.1177/0009922815574076.
6. Harvey, B.M.; Langford, J.E.; Harthoorn, L.F.; Gillman, S.A.; Green, T.D.; Schwartz, R.H.; Burks, A.W. Effects on growth and tolerance and hypoallergenicity of an amino acid-based formula with synbiotics. *Pediatr Res* **2014**, *75*, 343-351, doi:10.1038/pr.2013.211.
7. Saavedra, J.M.; Abi-Hanna, A.; Moore, N.; Yolken, R.H. Long-term consumption of infant formulas containing live probiotic bacteria: tolerance and safety. *Am J Clin Nutr* **2004**, *79*, 261-267, doi:10.1093/ajcn/79.2.261.
8. Scalabrin, D.M.; Johnston, W.H.; Hoffman, D.R.; P'Pool, V.L.; Harris, C.L.; Mitmesser, S.H. Growth and tolerance of healthy term infants receiving hydrolyzed infant formulas supplemented with Lactobacillus rhamnosus GG: randomized, double-blind, controlled trial. *Clin Pediatr (Phila)* **2009**, *48*, 734-744, doi:10.1177/0009922809332682.
9. Smilowitz, J.T.; Moya, J.; Breck, M.A.; Cook, C.; Fineberg, A.; Angkustsiri, K.; Underwood, M.A. Safety and tolerability of Bifidobacterium longum subspecies infantis EVC001 supplementation in healthy term breastfed infants: a phase I clinical trial. *BMC Pediatr* **2017**, *17*, 133, doi:10.1186/s12887-017-0886-9.
10. Underwood, M.A.; Salzman, N.H.; Bennett, S.H.; Barman, M.; Mills, D.A.; Marcobal, A.; Tancredi, D.J.; Bevins, C.L.; Sherman, M.P. A randomized placebo-controlled comparison of 2 prebiotic/probiotic combinations in preterm infants: impact on weight gain, intestinal microbiota, and fecal short-chain fatty acids. *J Pediatr Gastroenterol Nutr* **2009**, *48*, 216-225, doi:10.1097/MPG.0b013e31818de195.
11. Ziegler, E.E.; Jeter, J.M.; Drulis, J.M.; Nelson, S.E.; Haschke, F.; Steenhout, P.; Brown, C.; Maire, J.C.; Hager, C. Formula with reduced content of improved, partially hydrolyzed protein and probiotics: Infant growth and health. *Monatsschrift fur Kinderheilkunde* **2003**, *151*, S65-S71, doi:10.1007/s00112-003-0804-0.
12. Ringel-Kulka, T.; Kotch, J.B.; Jensen, E.T.; Savage, E.; Weber, D.J. Randomized, double-blind, placebo-controlled study of synbiotic yogurt effect on the health of children. *J Pediatr* **2015**, *166*, 1475-1481 e1471-1473, doi:10.1016/j.jpeds.2015.02.038.

13. Escribano, J.; Ferre, N.; Gispert-Llaurado, M.; Luque, V.; Rubio-Torrents, C.; Zaragoza-Jordana, M.; Polanco, I.; Codoner, F.M.; Chenoll, E.; Morera, M.; et al. Bifidobacterium longum subsp infantis CECT7210-supplemented formula reduces diarrhea in healthy infants: a randomized controlled trial. *Pediatr Res* **2018**, *83*, 1120-1128, doi:10.1038/pr.2018.34.
14. Gil-Campos, M.; Lopez, M.A.; Rodriguez-Benitez, M.V.; Romero, J.; Roncero, I.; Linares, M.D.; Maldonado, J.; Lopez-Huertas, E.; Berwind, R.; Ritzenthaler, K.L.; et al. Lactobacillus fermentum CECT 5716 is safe and well tolerated in infants of 1-6 months of age: a randomized controlled trial. *Pharmacol Res* **2012**, *65*, 231-238, doi:10.1016/j.phrs.2011.11.016.
15. Maldonado, J.; Canabate, F.; Sempere, L.; Vela, F.; Sanchez, A.R.; Narbona, E.; Lopez-Huertas, E.; Geerlings, A.; Valero, A.D.; Olivares, M.; et al. Human milk probiotic Lactobacillus fermentum CECT5716 reduces the incidence of gastrointestinal and upper respiratory tract infections in infants. *J Pediatr Gastroenterol Nutr* **2012**, *54*, 55-61, doi:10.1097/MPG.0b013e3182333f18.
16. Maldonado, J.; Gil-Campos, M.; Maldonado-Lobon, J.A.; Benavides, M.R.; Flores-Rojas, K.; Jaldo, R.; Jimenez Del Barco, I.; Bolivar, V.; Valero, A.D.; Prados, E.; et al. Evaluation of the safety, tolerance and efficacy of 1-year consumption of infant formula supplemented with Lactobacillus fermentum CECT5716 Lc40 or Bifidobacterium breve CECT7263: a randomized controlled trial. *BMC Pediatr* **2019**, *19*, 361, doi:10.1186/s12887-019-1753-7.
17. Maldonado, J.; Lara-Villoslada, F.; Sierra, S.; Sempere, L.; Gomez, M.; Rodriguez, J.M.; Boza, J.; Xaus, J.; Olivares, M. Safety and tolerance of the human milk probiotic strain Lactobacillus salivarius CECT5713 in 6-month-old children. *Nutrition* **2010**, *26*, 1082-1087, doi:10.1016/j.nut.2009.08.023.
18. Manzano, S.; De Andres, J.; Castro, I.; Rodriguez, J.M.; Jimenez, E.; Espinosa-Martos, I. Safety and tolerance of three probiotic strains in healthy infants: a multi-centre randomized, double-blind, placebo-controlled trial. *Benef Microbes* **2017**, *8*, 569-578, doi:10.3920/bm2017.0009.
19. Chouraqui, J.P.; Grathwohl, D.; Labaune, J.M.; Hascoet, J.M.; de Montgolfier, I.; Leclaire, M.; Giarre, M.; Steenhout, P. Assessment of the safety, tolerance, and protective effect against diarrhea of infant formulas containing mixtures of probiotics or probiotics and prebiotics in a randomized controlled trial. *Am J Clin Nutr* **2008**, *87*, 1365-1373, doi:10.1093/ajcn/87.5.1365.
20. Hays, S.; Jacquot, A.; Gauthier, H.; Kempf, C.; Beissel, A.; Pidoux, O.; Jumas-Bilak, E.; Decullier, E.; Lachambre, E.; Beck, L.; et al. Probiotics and growth in preterm infants: a randomized controlled trial, PREMAPRO study. *Clinical nutrition (Edinburgh, Scotland)* **2015**, doi:10.1016/j.clnu.2015.06.006.
21. Radke, M.; Picaud, J.C.; Loui, A.; Cambonie, G.; Faas, D.; Lafeber, H.N.; de Groot, N.; Pecquet, S.S.; Steenhout, P.G.; Hascoet, J.M. Starter formula enriched in prebiotics and probiotics ensures normal growth of infants and promotes gut health: a randomized clinical trial. *Pediatr Res* **2017**, *81*, 622-631, doi:10.1038/pr.2016.270.
22. Rouge, C.; Piloquet, H.; Butel, M.J.; Berger, B.; Rochat, F.; Ferraris, L.; Des Robert, C.; Legrand, A.; de la Cochetiere, M.F.; N'Guyen, J.M.; et al. Oral supplementation with

probiotics in very-low-birth-weight preterm infants: a randomized, double-blind, placebo-controlled trial. *Am J Clin Nutr* **2009**, *89*, 1828-1835, doi:10.3945/ajcn.2008.26919.
23. Roze, J.C.; Barbarot, S.; Butel, M.J.; Kapel, N.; Waligora-Dupriet, A.J.; De Montgolfier, I.; Leblanc, M.; Godon, N.; Soulaines, P.; Darmaun, D.; et al. An alpha-lactalbumin-enriched and symbiotic-supplemented v. a standard infant formula: a multicentre, double-blind, randomised trial. *Br J Nutr* **2012**, *107*, 1616-1622, doi:10.1017/s000711451100479x.
24. Thibault, H.; Aubert-Jacquin, C.; Goulet, O. Effects of long-term consumption of a fermented infant formula (with Bifidobacterium breve c50 and Streptococcus thermophilus 065) on acute diarrhea in healthy infants. *J Pediatr Gastroenterol Nutr* **2004**, *39*, 147-152, doi:10.1097/00005176-200408000-00004.
25. Abrahamse-Berkeveld, M.; Alles, M.; Franke-Beckmann, E.; Helm, K.; Knecht, R.; Kollges, R.; Sandner, B.; Knol, J.; Ben Amor, K.; Bufe, A. Infant formula containing galacto-and fructo-oligosaccharides and Bifidobacterium breve M-16V supports adequate growth and tolerance in healthy infants in a randomised, controlled, double-blind, prospective, multicentre study. *J Nutr Sci* **2016**, *5*, e42, doi:10.1017/jns.2016.35.
26. Ahrens, B.; Hellmuth, C.; Haiden, N.; Olbertz, D.; Hamelmann, E.; Vusurovic, M.; Fleddermann, M.; Roehle, R.; Knoll, A.; Koletzko, B.; et al. Hydrolyzed Formula With Reduced Protein Content Supports Adequate Growth: A Randomized Controlled Noninferiority Trial. *J Pediatr Gastroenterol Nutr* **2018**, *66*, 822-830, doi:10.1097/mpg.0000000000001853.
27. Bazanella, M.; Maier, T.V.; Clavel, T.; Lagkouvardos, I.; Lucio, M.; Maldonado-Gòmez, M.X.; Autran, C.; Walter, J.; Bode, L.; Schmitt-Kopplin, P.; et al. Randomized controlled trial on the impact of early-life intervention with bifidobacteria on the healthy infant fecal microbiota and metabolome. *American Journal of Clinical Nutrition* **2017**, *106*, 1274-1286, doi:10.3945/ajcn.117.157529.
28. Mohan, R.; Koebnick, C.; Schildt, J.; Mueller, M.; Radke, M.; Blaut, M. Effects of Bifidobacterium lactis Bb12 supplementation on body weight, fecal pH, acetate, lactate, calprotectin, and IgA in preterm infants. *Pediatr Res* **2008**, *64*, 418-422, doi:10.1203/PDR.0b013e318181b7fa.
29. Mitra, M.; Adarsh, E.; Narang, A.; Agrawal, R.; Vaidya, U.; Ganguly, S. Safety and tolerance of infant formulas containing probiotics in India: a multicenter randomized controlled trial. *Journal of maternal-fetal & neonatal medicine* **2014**, *27*, 346-, doi:10.3109/14767058.2014.924236.
30. Panigrahi, P.; Parida, S.; Nanda, N.C.; Satpathy, R.; Pradhan, L.; Chandel, D.S.; Baccaglini, L.; Mohapatra, A.; Mohapatra, S.S.; Misra, P.R.; et al. A randomized synbiotic trial to prevent sepsis among infants in rural India. *Nature* **2017**, *548*, 407-412, doi:10.1038/nature23480.
31. Panigrahi, P.; Parida, S.; Pradhan, L.; Mohapatra, S.S.; Misra, P.R.; Johnson, J.A.; Chaudhry, R.; Taylor, S.; Hansen, N.I.; Gewolb, I.H. Long-term colonization of a Lactobacillus plantarum synbiotic preparation in the neonatal gut. *J Pediatr Gastroenterol Nutr* **2008**, *47*, 45-53, doi:10.1097/MPG.0b013e31815a5f2c.
32. Sazawal, S.; Dhingra, U.; Hiremath, G.; Sarkar, A.; Dhingra, P.; Dutta, A.; Menon, V.P.; Black, R.E. Effects of Bifidobacterium lactis HN019 and prebiotic oligosaccharide added

to milk on iron status, anemia, and growth among children 1 to 4 years old. *J Pediatr Gastroenterol Nutr* **2010**, *51*, 341-346, doi:10.1097/MPG.0b013e3181d98e45.
33. Sur, D.; Manna, B.; Niyogi, S.K.; Ramamurthy, T.; Palit, A.; Nomoto, K.; Takahashi, T.; Shima, T.; Tsuji, H.; Kurakawa, T.; et al. Role of probiotic in preventing acute diarrhoea in children: a community-based, randomized, double-blind placebo-controlled field trial in an urban slum. *Epidemiol Infect* **2011**, *139*, 919-926, doi:10.1017/S0950268810001780.
34. Demirel, G.; Erdeve, O.; Celik, I.H.; Dilmen, U. Saccharomyces boulardii for prevention of necrotizing enterocolitis in preterm infants: a randomized, controlled study. *Acta Paediatr* **2013**, *102*, e560-565, doi:10.1111/apa.12416.
35. Dilli, D.; Aydin, B.; Fettah, N.D.; Ozyazici, E.; Beken, S.; Zenciroglu, A.; Okumus, N.; Ozyurt, B.M.; Ipek, M.S.; Akdag, A.; et al. The propre-save study: effects of probiotics and prebiotics alone or combined on necrotizing enterocolitis in very low birth weight infants. *J Pediatr* **2015**, *166*, 545-551 e541, doi:10.1016/j.jpeds.2014.12.004.
36. Guney Varal, I.; Koksal, N.; Ozkan, H.; Bagci, O.; Dogan, P. Potential use of multi-strain synbiotics for improving postnatal head circumference. *Pakistan Journal of Medical Sciences* **2018**, *34*, 1502-1506, doi:10.12669/pjms.346.16107.
37. Sari, F.N.; Dizdar, E.A.; Oguz, S.; Erdeve, O.; Uras, N.; Dilmen, U. Oral probiotics: Lactobacillus sporogenes in prevention of necrotizing enterocolitis in very low birth weight infants: A randomized, controlled trial. *Early Human Development* **2010**, *86*, S87, doi:10.1016/j.earlhumdev.2010.09.229.
38. Serce, O.; Benzer, D.; Gursoy, T.; Karatekin, G.; Ovali, F. Efficacy of Saccharomyces boulardii on necrotizing enterocolitis or sepsis in very low birth weight infants: a randomised controlled trial. *Early Hum Dev* **2013**, *89*, 1033-1036, doi:10.1016/j.earlhumdev.2013.08.013.
39. Cui, X.; Shi, Y.; Gao, S.; Xue, X.; Fu, J. Effects of Lactobacillus reuteri DSM 17938 in preterm infants: a double-blinded randomized controlled study. *Ital J Pediatr* **2019**, *45*, 140, doi:10.1186/s13052-019-0716-9.
40. Li, X.; Peng, Y.; Li, Z.; Christensen, B.; Heckmann, A.B.; Stenlund, H.; Lönnerdal, B.; Hernell, O. Feeding Infants Formula With Probiotics or Milk Fat Globule Membrane: A Double-Blind, Randomized Controlled Trial. *Frontiers in Pediatrics* **2019**, *7*, doi:10.3389/fped.2019.00347.
41. Xiao, L.; Gong, C.; Ding, Y.; Ding, G.; Xu, X.; Deng, C.; Ze, X.; Malard, P.; Ben, X. Probiotics maintain intestinal secretory immunoglobulin A levels in healthy formula-fed infants: A randomised, double-blind, placebo-controlled study. *Beneficial Microbes* **2019**, *10*, 729-739, doi:10.3920/BM2019.0025.
42. Xu, L.; Wang, Y.; Wang, Y.; Fu, J.; Sun, M.; Mao, Z.; Vandenplas, Y. A double-blinded randomized trial on growth and feeding tolerance with Saccharomyces boulardii CNCM I-745 in formula-fed preterm infants. *J Pediatr (Rio J)* **2016**, *92*, 296-301, doi:10.1016/j.jped.2015.08.013.
43. Urban, M.F.; Bolton, K.D.; Mokhachane, M.; Mphahlele, R.M.; Bomela, H.N.; Monaheng, L.; Beckh-Arnold, E.; Cooper, P.A. Growth of infants born to HIV-infected women when fed a biologically acidified starter formula with and without probiotics. *South African Journal of Clinical Nutrition* **2008**, *21*, 28-32, doi:10.1080/16070658.2008.11734148.

44. Velaphi, S.C.; Cooper, P.A.; Bolton, K.D.; Mokhachane, M.; Mphahlele, R.M.; Beckh-Arnold, E.; Monaheng, L.; Haschke-Becher, E. Growth and metabolism of infants born to women infected with human immunodeficiency virus and fed acidified whey-adapted starter formulas. *Nutrition* **2008**, *24*, 203-211, doi:10.1016/j.nut.2007.11.002.
45. Aloisio, I.; Prodam, F.; Giglione, E.; Bozzi Cionci, N.; Solito, A.; Bellone, S.; Baffoni, L.; Mogna, L.; Pane, M.; Bona, G.; et al. Three-Month Feeding Integration With Bifidobacterium Strains Prevents Gastrointestinal Symptoms in Healthy Newborns. *Front Nutr* **2018**, *5*, 39, doi:10.3389/fnut.2018.00039.
46. Indrio, F.; Riezzo, G.; Raimondi, F.; Bisceglia, M.; Cavallo, L.; Francavilla, R. The effects of probiotics on feeding tolerance, bowel habits, and gastrointestinal motility in preterm newborns. *J Pediatr* **2008**, *152*, 801-806, doi:10.1016/j.jpeds.2007.11.005.
47. Puccio, G.; Cajozzo, C.; Meli, F.; Rochat, F.; Grathwohl, D.; Steenhout, P. Clinical evaluation of a new starter formula for infants containing live Bifidobacterium longum BL999 and prebiotics. *Nutrition* **2007**, *23*, 1-8, doi:10.1016/j.nut.2006.09.007.
48. Meli, F.; Puccio, G.; Cajozzo, C.; Ricottone, G.L.; Pecquet, S.; Sprenger, N.; Steenhout, P. Growth and safety evaluation of infant formulae containing oligosaccharides derived from bovine milk: a randomized, double-blind, noninferiority trial. *BMC Pediatr* **2014**, *14*, 306, doi:10.1186/s12887-014-0306-3.
49. Kankaanpaa, P.E.; Yang, B.; Kallio, H.P.; Isolauri, E.; Salminen, S.J. Influence of probiotic supplemented infant formula on composition of plasma lipids in atopic infants. *J Nutr Biochem* **2002**, *13*, 364-369, doi:10.1016/s0955-2863(02)00185-7.
50. Kukkonen, K.; Savilahti, E.; Haahtela, T.; Juntunen-Backman, K.; Korpela, R.; Poussa, T.; Tuure, T.; Kuitunen, M. Long-term safety and impact on infection rates of postnatal probiotic and prebiotic (synbiotic) treatment: randomized, double-blind, placebo-controlled trial. *Pediatrics* **2008**, *122*, 8-12, doi:10.1542/peds.2007-1192.
51. Luoto, R.; Kalliomaki, M.; Laitinen, K.; Isolauri, E. The impact of perinatal probiotic intervention on the development of overweight and obesity: follow-up study from birth to 10 years. *Int J Obes (Lond)* **2010**, *34*, 1531-1537, doi:10.1038/ijo.2010.50.
52. Costalos, C.; Skouteri, V.; Gounaris, A.; Sevastiadou, S.; Triandafilidou, A.; Ekonomidou, C.; Kontaxaki, F.; Petrochilou, V. Enteral feeding of premature infants with Saccharomyces boulardii. *Early Hum Dev* **2003**, *74*, 89-96, doi:10.1016/s0378-3782(03)00090-2.
53. Papagaroufalis, K.; Fotiou, A.; Egli, D.; Tran, L.A.; Steenhout, P. A Randomized Double Blind Controlled Safety Trial Evaluating d-Lactic Acid Production in Healthy Infants Fed a Lactobacillus reuteri-containing Formula. *Nutr Metab Insights* **2014**, *7*, 19-27, doi:10.4137/NMI.S14113.
54. Stratiki, Z.; Costalos, C.; Sevastiadou, S.; Kastanidou, O.; Skouroliakou, M.; Giakoumatou, A.; Petrohilou, V. The effect of a bifidobacter supplemented bovine milk on intestinal permeability of preterm infants. *Early Hum Dev* **2007**, *83*, 575-579, doi:10.1016/j.earlhumdev.2006.12.002.
55. Bin-Nun, A.; Bromiker, R.; Wilschanski, M.; Kaplan, M.; Rudensky, B.; Caplan, M.; Hammerman, C. Oral probiotics prevent necrotizing enterocolitis in very low birth weight neonates. *J Pediatr* **2005**, *147*, 192-196, doi:10.1016/j.jpeds.2005.03.054.

56. Weizman, Z.; Alsheikh, A. Safety and tolerance of a probiotic formula in early infancy comparing two probiotic agents: a pilot study. *J Am Coll Nutr* **2006**, *25*, 415-419, doi:10.1080/07315724.2006.10719554.
57. Weizman, Z.; Asli, G.; Alsheikh, A. Effect of a probiotic infant formula on infections in child care centers: comparison of two probiotic agents. *Pediatrics* **2005**, *115*, 5-9, doi:10.1542/peds.2004-1815.
58. Allen, S.J.; Jordan, S.; Storey, M.; Thornton, C.A.; Gravenor, M.; Garaiova, I.; Plummer, S.F.; Wang, D.; Morgan, G. Dietary supplementation with lactobacilli and bifidobacteria is well tolerated and not associated with adverse events during late pregnancy and early infancy. *J Nutr* **2010**, *140*, 483-488, doi:10.3945/jn.109.117093.
59. Costeloe, K.; Hardy, P.; Juszczak, E.; Wilks, M.; Millar, M.R. Bifidobacterium breve BBG-001 in very preterm infants: a randomised controlled phase 3 trial. *Lancet* **2016**, *387*, 649-660, doi:10.1016/s0140-6736(15)01027-2.
60. Millar, M.R.; Bacon, C.; Smith, S.L.; Walker, V.; Hall, M.A. Enteral feeding of premature infants with Lactobacillus GG. *Arch Dis Child* **1993**, *69*, 483-487, doi:10.1136/adc.69.5_spec_no.483.
61. Jacobs, S.E.; Tobin, J.M.; Opie, G.F.; Donath, S.; Tabrizi, S.N.; Pirotta, M.; Morley, C.J.; Garland, S.M.; ProPrems Study, G. Probiotic effects on late-onset sepsis in very preterm infants: a randomized controlled trial. *Pediatrics* **2013**, *132*, 1055-1062, doi:10.1542/peds.2013-1339.
62. Taylor, A.L.; Dunstan, J.A.; Prescott, S.L. Probiotic supplementation for the first 6 months of life fails to reduce the risk of atopic dermatitis and increases the risk of allergen sensitization in high-risk children: a randomized controlled trial. *J Allergy Clin Immunol* **2007**, *119*, 184-191, doi:10.1016/j.jaci.2006.08.036.
63. Dekker, J.W.; Wickens, K.; Black, P.N.; Stanley, T.V.; Mitchell, E.A.; Fitzharris, P.; Tannock, G.W.; Purdie, G.; Crane, J. Safety aspects of probiotic bacterial strains Lactobacillus rhamnosus HN001 and Bifidobacterium animalis subsp. lactis HN019 in human infants aged 0-2 years. *International Dairy Journal* **2009**, *19*, 149-154, doi:10.1016/j.idairyj.2008.10.004.
64. Vlieger, A.M.; Robroch, A.; van Buuren, S.; Kiers, J.; Rijkers, G.; Benninga, M.A.; te Biesebeke, R. Tolerance and safety of Lactobacillus paracasei ssp. paracasei in combination with Bifidobacterium animalis ssp. lactis in a prebiotic-containing infant formula: a randomised controlled trial. *Br J Nutr* **2009**, *102*, 869-875, doi:10.1017/s0007114509289069.
65. Agustina, R.; Bovee-Oudenhoven, I.M.; Lukito, W.; Fahmida, U.; van de Rest, O.; Zimmermann, M.B.; Firmansyah, A.; Wulanti, R.; Albers, R.; van den Heuvel, E.G.; et al. Probiotics Lactobacillus reuteri DSM 17938 and Lactobacillus casei CRL 431 modestly increase growth, but not iron and zinc status, among Indonesian children aged 1-6 years. *J Nutr* **2013**, *143*, 1184-1193, doi:10.3945/jn.112.166397.
66. Surono, I.S.; Koestomo, F.P.; Novitasari, N.; Zakaria, F.R.; Yulianasari; Koesnandar. Novel probiotic Enterococcus faecium IS-27526 supplementation increased total salivary sIgA level and bodyweight of pre-school children: a pilot study. *Anaerobe* **2011**, *17*, 496-500, doi:10.1016/j.anaerobe.2011.06.003.

67. Amini, E.; Dalili, H.; Niknafs, N.; Shariat, M.; Nakhostin, M.; Jedari-Attari, S. The effect of probiotics in prevention of necrotising enterocolitis in preterm neonates in comparison with control group. *Iranian Journal of Pediatrics* **2017**, *27*, doi:10.5812/ijp.7663.
68. Jalali, S.Z.; Shiri, M.R.; Shirazi, M.G. Effect of probiotics on full intestinal feeding in premature infants: a double blind, clinical trial. *Iranian journal of pediatrics* **2020**, *30*, 1-6, doi:10.5812/ijp.100139.
69. Chrzanowska-Liszewska, D.; Seliga-Siwecka, J.; Kornacka, M.K. The effect of Lactobacillus rhamnosus GG supplemented enteral feeding on the microbiotic flora of preterm infants-double blinded randomized control trial. *Early Hum Dev* **2012**, *88*, 57-60, doi:10.1016/j.earlhumdev.2011.07.002.
70. Szajewska, H.; Ruszczynski, M.; Szymanski, H.; Sadowska-Krawczenko, I.; Piwowarczyk, A.; Rasmussen, P.B.; Kristensen, M.B.; West, C.E.; Hernell, O. Effects of infant formula supplemented with prebiotics compared with synbiotics on growth up to the age of 12 mo: a randomized controlled trial. *Pediatr Res* **2017**, *81*, 752-758, doi:10.1038/pr.2017.5.
71. Totsu, S.; Yamasaki, C.; Terahara, M.; Uchiyama, A.; Kusuda, S. Bifidobacterium and enteral feeding in preterm infants: cluster-randomized trial. *Pediatr Int* **2014**, *56*, 714-719, doi:10.1111/ped.12330.
72. Oshiro, T.; Nagata, S.; Wang, C.; Takahashi, T.; Tsuji, H.; Asahara, T.; Nomoto, K.; Takei, H.; Nittono, H.; Yamashiro, Y. Bifidobacterium Supplementation of Colostrum and Breast Milk Enhances Weight Gain and Metabolic Responses Associated with Microbiota Establishment in Very-Preterm Infants. *Biomedicine Hub* **2019**, *4*, doi:10.1159/000502935.
73. Fisberg, M.; Maulén-Radován, I.E.; Tormo, R.; Tabernero Carrascoso, M.; Pedrón Giner, C.; Argüelles Martin, F.; Pavón Belinchón, P.; Martínez Costa, C.; Pérez Pérez, M.; Gonzalez Caro, J.; et al. Effect of oral nutritional supplementation with or without synbiotics on sickness and catch-up growth in preschool children. *International Pediatrics* **2002**, *17*, 216-222.
74. Hojsak, I.; Snovak, N.; Abdovic, S.; Szajewska, H.; Misak, Z.; Kolacek, S. Lactobacillus GG in the prevention of gastrointestinal and respiratory tract infections in children who attend day care centers: a randomized, double-blind, placebo-controlled trial. *Clin Nutr* **2010**, *29*, 312-316, doi:10.1016/j.clnu.2009.09.008.
75. Vendt, N.; Grunberg, H.; Tuure, T.; Malminiemi, O.; Wuolijoki, E.; Tillmann, V.; Sepp, E.; Korpela, R. Growth during the first 6 months of life in infants using formula enriched with Lactobacillus rhamnosus GG: double-blind, randomized trial. *J Hum Nutr Diet* **2006**, *19*, 51-58, doi:10.1111/j.1365-277X.2006.00660.x.
76. Batac, M.C.R.; Guno, M.J.V.; Caparas-de Castro, C.; Gutierrez-Santos, K.; Tondoc, A. Effects of a probiotic formula on measles, mumps and rubella IgG production and on anthropometric measurements of infants aged 11-15 months in a tertiary hospital. *Santo Tomas Journal of Medicine* **2005**, *52*, 124-130.
77. Wejryd, E.; Marchini, G.; Frimmel, V.; Jonsson, B.; Abrahamsson, T. Probiotics promoted head growth in extremely low birthweight infants in a double-blind placebo-controlled trial. *Acta Paediatr* **2019**, *108*, 62-69, doi:10.1111/apa.14497.

78. Simeoni, U.; Berger, B.; Junick, J.; Blaut, M.; Pecquet, S.; Rezzonico, E.; Grathwohl, D.; Sprenger, N.; Brussow, H.; Study, T.; et al. Gut microbiota analysis reveals a marked shift to bifidobacteria by a starter infant formula containing a synbiotic of bovine milk-derived oligosaccharides and Bifidobacterium animalis subsp. lactis CNCM I-3446. *Environ Microbiol* **2016**, *18*, 2185-2195, doi:10.1111/1462-2920.13144.
79. Lin, H.C.; Hsu, C.H.; Chen, H.L.; Chung, M.Y.; Hsu, J.F.; Lien, R.I.; Tsao, L.Y.; Chen, C.H.; Su, B.H. Oral probiotics prevent necrotizing enterocolitis in very low birth weight preterm infants: a multicenter, randomized, controlled trial. *Pediatrics* **2008**, *122*, 693-700, doi:10.1542/peds.2007-3007.
80. Saengtawesin, V.; Tangpolkaiwalsak, R.; Kanjanapattankul, W. Effect of oral probiotics supplementation in the prevention of necrotizing enterocolitis among very low birth weight preterm infants. *Chotmaihet thangphaet [journal of the medical association of thailand]* **2014**, *97*, S20-S25.
81. Xuan, N.N.; Wang, D.; Grathwohl, D.; Lan, P.N.; Kim, H.V.; Goyer, A.; Benyacoub, J. Effect of a Growing-up Milk Containing Synbiotics on Immune Function and Growth in Children: A Cluster Randomized, Multicenter, Double-blind, Placebo Controlled Study. *Clin Med Insights Pediatr* **2013**, *7*, 49-56, doi:10.4137/CMPed.S13073.
82. Abdulkadir, B.; Nelson, A.; Skeath, T.; Marrs, E.C.; Perry, J.D.; Cummings, S.P.; Embleton, N.D.; Berrington, J.E.; Stewart, C.J. Routine Use of Probiotics in Preterm Infants: Longitudinal Impact on the Microbiome and Metabolome. *Neonatology* **2016**, *109*, 239-247, doi:10.1159/000442936.
83. Abramova, T.V.; Gmoshinskaya, M.V.; Surzhik, A.V.; Kon, I.Y.; Georgieva, O.V.; Safronova, A.I.; Pustograev, N.N.; Kurkova, V.I.; Kuleshova, O.K.; Rusova, T.V. Parameters of physical and psychomotor development in infants of the first months of life who receive an adapted milk formula with prebiotics, nucleotides and long-chain polyunsaturated fatty acids. *Voprosy Prakticheskoi Pediatrii* **2015**, *10*, 30-38.
84. Actrn. Use of probiotics to improve gut health and vaccine response in newborn babies. *http://www.who.int/trialsearch/Trial2.aspx?TrialID=ACTRN12619000369123* **2019**.
85. Actrn. The effect of probiotic on the weight gaining and feeding intolerance in preterm newborn babies of Kabul city via a randomized clinical trial. *http://www.who.int/trialsearch/Trial2.aspx?TrialID=ACTRN12620000538943* **2020**.
86. Aflatoonian, M.; Taghavi Ardakani, A.; Modarresi, S.Z.; Modaresi, V.; Karimi, M.; Ordooei, M.; Vakili, M.; Pakseresht, B. The Effect of Synbiotic Supplementation on Growth Parameters in Mild to Moderate FTT Children Aged 2–5 Years. *Probiotics and antimicrobial proteins* **2019**, doi:10.1007/s12602-018-9508-6.
87. Akar, M.; Eras, Z.; Oncel, M.Y.; Arayici, S.; Guzoglu, N.; Canpolat, F.E.; Uras, N.; Oguz, S.S. Impact of oral probiotics on neurodevelopmental outcomes in preterm infants. *J Matern Fetal Neonatal Med* **2017**, *30*, 411-415, doi:10.1080/14767058.2016.1174683.
88. Aydin, B.; Dilli, D.; Erol, S.; Sorguc, N.H.; Beken, S.; Cullas Ilarslan, N.E.; Zenciroglu, A.; Okumus, N. The effects of synbiotic use on morbidity and mortality in premature infants: A prospective randomized controlled trial. *Archives of Disease in Childhood* **2012**, *97*, A462, doi:10.1136/archdischild-2012-302724.1634.
89. Baglatzi, L.; Gavrili, S.; Stamouli, K.; Zachaki, S.; Favre, L.; Pecquet, S.; Benyacoub, J.; Costalos, C. Effect of Infant Formula Containing a Low Dose of the Probiotic

Bifidobacterium lactis CNCM I-3446 on Immune and Gut Functions in C-Section Delivered Babies: A Pilot Study. *Clin Med Insights Pediatr* **2016**, *10*, 11-19, doi:10.4137/CMPed.S33096.
90. Bakker-Zierikzee, A.M.; Alles, M.S.; Knol, J.; Kok, F.J.; Tolboom, J.J.; Bindels, J.G. Effects of infant formula containing a mixture of galacto- and fructo-oligosaccharides or viable Bifidobacterium animalis on the intestinal microflora during the first 4 months of life. *Br J Nutr* **2005**, *94*, 783-790, doi:10.1079/bjn20051451.
91. Bakker-Zierikzee, A.M.; Tol, E.A.; Kroes, H.; Alles, M.S.; Kok, F.J.; Bindels, J.G. Faecal SIgA secretion in infants fed on pre- or probiotic infant formula. *Pediatr Allergy Immunol* **2006**, *17*, 134-140, doi:10.1111/j.1399-3038.2005.00370.x.
92. Barclay, D.; Puccio, G.; Fazzolari-Nesci, A.; Giammanco, A.; Raiha, N.; Carrie Fassler, A.L.; Brown, C.; Chauffard, F.; Grathwohl, D.; Hager, C.; et al. Growth and tolerance of a whey-based starter infant formula with enhanced protein efficiency and containing pro-, pre- or synbiotics. A randomized controlled trial in term infants. *Journal of pediatric gastroenterology and nutrition* **2003**, *37*, 388.
93. Berggren, A.; Söderberg, L.; Önning, G.; Hagslätt, M.L.J.; Axelsson, I. Intestinal Function, Microflora and Nutrient Intake of Children after Administration of a Fermented Oat Product Containing Lactobacillus plantarum DSM 9843 (299v). *Microbial Ecology in Health and Disease* **2003**, *15*, 160-168, doi:10.1080/08910600410024825.
94. Binns, C.W.; Lee, A.H.; Harding, H.; Gracey, M.; Barclay, D.V. The CUPDAY Study: prebiotic-probiotic milk product in 1-3-year-old children attending childcare centres. *Acta Paediatr* **2007**, *96*, 1646-1650, doi:10.1111/j.1651-2227.2007.00508.x.
95. Bocquet, A.; Lachambre, E.; Kempf, C.; Beck, L. Effect of infant and follow-on formulas containing B lactis and galacto- and fructo-oligosaccharides on infection in healthy term infants. *J Pediatr Gastroenterol Nutr* **2013**, *57*, 180-187, doi:10.1097/MPG.0b013e318297f35e.
96. Braga, T.D.; da Silva, G.A.; de Lira, P.I.; de Carvalho Lima, M. Efficacy of Bifidobacterium breve and Lactobacillus casei oral supplementation on necrotizing enterocolitis in very-low-birth-weight preterm infants: a double-blind, randomized, controlled trial. *Am J Clin Nutr* **2011**, *93*, 81-86, doi:10.3945/ajcn.2010.29799.
97. Campeotto, F.; Suau, A.; Kapel, N.; Magne, F.; Viallon, V.; Ferraris, L.; Waligora-Dupriet, A.J.; Soulaines, P.; Leroux, B.; Kalach, N.; et al. A fermented formula in pre-term infants: clinical tolerance, gut microbiota, down-regulation of faecal calprotectin and up-regulation of faecal secretory IgA. *Br J Nutr* **2011**, *105*, 1843-1851, doi:10.1017/S0007114510005702.
98. Campoy, C.; Nieto-Ruiz, A.; Sepúlveda-Valbuena, N.; Diéguez, E.; Herrmann, F.; Miranda, M.T.; De Castellar, R. Association of early nutrition and gender with metabolic risk in healthy children at 4 years of age. *Annals of Nutrition and Metabolism* **2018**, *73*, 44-45, doi:10.1159/000490752.
99. Castanet, M.; Costalos, C.; Haiden, N.; Hascoet, J.M.; Berger, B.; Sprenger, N.; Grathwohl, D.; Brussow, H.; De Groot, N.; Steenhout, P.; et al. Early Effect of Supplemented Infant Formulae on Intestinal Biomarkers and Microbiota: A Randomized Clinical Trial. *Nutrients* **2020**, *12*, doi:10.3390/nu12051481.

100. Cazzola, M.; Pham-Thi, N.; Kerihuel, J.C.; Durand, H.; Bohbot, S. Efficacy of a synbiotic supplementation in the prevention of common winter diseases in children: a randomized, double-blind, placebo-controlled pilot study. *Ther Adv Respir Dis* **2010**, *4*, 271-278, doi:10.1177/1753465810379010.
101. Chau, K.; Lau, E.; Greenberg, S.; Jacobson, S.; Yazdani-Brojeni, P.; Verma, N.; Koren, G. Probiotics for infantile colic: a randomized, double-blind, placebo-controlled trial investigating Lactobacillus reuteri DSM 17938. *J Pediatr* **2015**, *166*, 74-78, doi:10.1016/j.jpeds.2014.09.020.
102. Chen, C.C.; Kong, M.S.; Lai, M.W.; Chao, H.C.; Chang, K.W.; Chen, S.Y.; Huang, Y.C.; Chiu, C.H.; Li, W.C.; Lin, P.Y.; et al. Probiotics have clinical, microbiologic, and immunologic efficacy in acute infectious diarrhea. *Pediatric Infectious Disease Journal* **2010**, *29*, 135-138, doi:10.1097/INF.0b013e3181b530bf.
103. Chi, C.; Xue, Y.; Liu, R.; Wang, Y.; Lv, N.; Zeng, H.; Buys, N.; Zhu, B.; Sun, J.; Yin, C. Effects of a formula with a probiotic Bifidobacterium lactis Supplement on the gut microbiota of low birth weight infants. *Eur J Nutr* **2019**, doi:10.1007/s00394-019-02006-4.
104. Chou, I.C.; Kuo, H.T.; Chang, J.S.; Wu, S.F.; Chiu, H.Y.; Su, B.H.; Lin, H.C. Lack of Effects of Oral Probiotics on Growth and Neurodevelopmental Outcomes in Preterm Very Low Birth Weight Infants. *Journal of pediatrics* **2010**, *156*, 393-396, doi:10.1016/j.jpeds.2009.09.051.
105. Chubarova, A.I.; Sharyafetdinova, G.R. An experience of using a preparation containing combined probiotic strains of bifidobacteria and lactobacilli in premature newborns in neonatal resuscitation and intensive care units. *Voprosy Detskoi Dietologii* **2017**, *15*, 5-13, doi:10.20953/1727-5784-2017-4.
106. Costeloe, K.; Wilks, M.; Hardy, P.; Millar, M. Bifidobacterium breve BBG-001 (B breve) to prevent necrotising enterocolitis (NEC), late-onset sepsis (LOS) and death: The pips trial. *Journal of Paediatrics and Child Health* **2015**, *51*, 67, doi:10.1111/jpc.12884-4.
107. Cox, M.J.; Huang, Y.J.; Fujimura, K.E.; Liu, J.T.; McKean, M.; Boushey, H.A.; Segal, M.R.; Brodie, E.L.; Cabana, M.D.; Lynch, S.V. Lactobacillus casei abundance is associated with profound shifts in the infant gut microbiome. *PLoS One* **2010**, *5*, e8745, doi:10.1371/journal.pone.0008745.
108. Ctri. A clinical trial to study the efficacy of probiotic VSL#3/PP in preventing Necrotizing enterocolitis in preterm infants. *http://www.who.int/trialsearch/Trial2.aspx?TrialID=CTRI/2009/091/000556* **2009**.
109. Ctri. Growth of infants who are supplementary fed / mixed fed with probiotics-containing formula in India. Available online: (accessed on
110. Ctri. A study of the Effect of Probiotic organism administration on Feeding Tolerance in Very Low Birth Weight Newborn babies. *http://www.who.int/trialsearch/Trial2.aspx?TrialID=CTRI/2012/08/002853* **2012**.
111. Ctri. Role of Probiotics on growth and diarrhea in children. *http://www.who.int/trialsearch/Trial2.aspx?TrialID=CTRI/2012/08/002942* **2012**.
112. Ctri. clinical trial comparing the long term outcomes of post discharge probiotic treatment in moderately premature infants. *http://www.who.int/trialsearch/Trial2.aspx?TrialID=CTRI/2017/12/011033* **2017**.

113. Da Costa Ribeiro Júnior, H.; Medrado Ribeiro, T.C.; Peixoto De Mattos, A.; Pontes, M.; Saccardo Sarni, R.O.; Santos Cruz, M.L.; Nogueira-De-Almeida, C.A.; Mussi-Pinhata, M.M.; De Carvalho Norton, R.; Steenhout, P. Normal Growth of Healthy Infants Born from HIV+ Mothers Fed a Reduced Protein Infant Formula Containing the Prebiotics Galacto-Oligosaccharides and Fructo-Oligosaccharides: A Randomized Controlled Trial. *Clinical Medicine Insights: Pediatrics* **2015**, 37-47, doi:10.4137/CMPed.S17841.
114. Di Pierro, F.; Lo Russo, P.; Danza, M.L.; Basile, I.; Soardo, S.; Capocasale, G.; Paparone, S.B.; Paletta, V.; Lanza, C.; Schiavone, E.; et al. Use of a probiotic mixture containing Bifidobacterium animalis subsp. lactis BB-12 and Enterococcus faecium L3 as prophylaxis to reduce the incidence of acute gastroenteritis and upper respiratory tract infections in children. *Minerva Pediatr* **2020**, doi:10.23736/S0026-4946.20.05925-3.
115. Drks. PRIMAL Clinical Study: efficacy of probiotics to prevent gut dysbiosis in very preterm infants (VPIs) and moderate preterm infants of 28+0 – 32+6 weeks of gestation: a randomized, placebo-controlled double-blind study. *http://www.who.int/trialsearch/Trial2.aspx?TrialID=DRKS00013197* **2018**.
116. Dupont, C.; Rivero, M.; Grillon, C.; Belaroussi, N.; Kalindjian, A.; Marin, V. Alpha-lactalbumin-enriched and probiotic-supplemented infant formula in infants with colic: growth and gastrointestinal tolerance. *Eur J Clin Nutr* **2010**, *64*, 765-767, doi:10.1038/ejcn.2010.81.
117. Famouri, F.; Khoshdel, A.; Golshani, A.; Kheiri, S.; Saneian, H.; Kelishadi, R. Effects of synbiotics on treatment of children with failure to thrive: A triple blind placebo-controlled trial. *Journal of Research in Medical Sciences* **2014**, *19*, 1046-1050.
118. Firmansyah, A.; Dwipoerwantoro, P.G.; Kadim, M.; Alatas, S.; Conus, N.; Lestarina, L.; Bouisset, F.; Steenhout, P. Improved growth of toddlers fed a milk containing synbiotics. *Asia Pac J Clin Nutr* **2011**, *20*, 69-76.
119. Fleming, P.; Panton, N.; Wilks, M.; Millar, M.; Costeloe, K. Comparing serum bacterial DNA and lipopolysaccharide in preterm infants randomised to bifidobacterium breve BBG-001 versus placebo. *Journal of Paediatrics and Child Health* **2015**, *51*, 67, doi:10.1111/jpc.12884-4.
120. Fonollá, J.; Maldonado-Lobón, J.A.; Gil-Campo, M.; Maldonado, J.; Flores, K.; Benavides, M.R.; Jaldo, R.; Del Barco, I.J.; Valero, A.D.; Lara, F.; et al. An infant formula enriched with the human milk strain Lactobacillus fermentum CECT5716 is safe and reduces dhiarrea incidences during first year of life. *Journal of Pediatric Gastroenterology and Nutrition* **2017**, *64*, 933, doi:10.1097/01.mpg.0000516381.25680.b4.
121. Galpin, L.; Manary, M.J.; Fleming, K.; Ou, C.N.; Ashorn, P.; Shulman, R.J. Effect of Lactobacillus GG on intestinal integrity in Malawian children at risk of tropical enteropathy. *Am J Clin Nutr* **2005**, *82*, 1040-1045, doi:10.1093/ajcn/82.5.1040.
122. Garland, S.M.; Tobin, J.M.; Pirotta, M.; Tabrizi, S.N.; Opie, G.; Donath, S.; Tang, M.L.; Morley, C.J.; Hickey, L.; Ung, L.; et al. The ProPrems trial: investigating the effects of probiotics on late onset sepsis in very preterm infants. *BMC Infect Dis* **2011**, *11*, 210, doi:10.1186/1471-2334-11-210.
123. Gibson, R.A.; Barclay, D.; Marshall, H.; Moulin, J.; Maire, J.C.; Makrides, M. Safety of supplementing infant formula with long-chain polyunsaturated fatty acids and

Bifidobacterium lactis in term infants: a randomised controlled trial. *Br J Nutr* **2009**, *101*, 1706-1713, doi:10.1017/s0007114508084080.
124. Gomez-Rodriguez, G.; Amador-Licona, N.; Daza-Benitez, L.; Barbosa-Sabanero, G.; Carballo-Magdaleno, D.; Aguilar-Padilla, R.; Gonzalez-Ramirez, E. Single strain versus multispecies probiotic on necrotizing enterocolitis and faecal IgA levels in very low birth weight preterm neonates: A randomized clinical trial. *Pediatr Neonatol* **2019**, *60*, 564-569, doi:10.1016/j.pedneo.2019.02.005.
125. Gonchar, N.V.; Suvorov, A.N.; Maryshev, V.P.; Sorokina, T.M.; Churkova, T.V.; Kharit, S.M. PROBIOTICS, NUTRITIONAL STATUS AND RESISTANCE TO RESPIRATORY INFECTIONS IN INFANTS. *E? Ksperimental'nai? A? I klinicheskai? A? Gastroe? Nterologii? A? = experimental & clinical gastroenterology* **2015**, 48-54.
126. Gonchar, N.V.; Lo Skiavo, L.A.; Suvorov, A.N. Effects of using a probiotic strain of enterococcus on the dynamics of body weight, reduction of the incidence of complications and change of gut microbiota in deeply premature newborn infants. *Voprosy Detskoi Dietologii* **2016**, *14*, 5-14, doi:10.20953/1727-5784-2016-1-5-14.
127. Grenov, B.; Namusoke, H.; Lanyero, B.; Nabukeera-Barungi, N.; Ritz, C.; Molgaard, C.; Friis, H.; Michaelsen, K.F. Effect of Probiotics on Diarrhea in Children With Severe Acute Malnutrition: A Randomized Controlled Study in Uganda. *J Pediatr Gastroenterol Nutr* **2017**, *64*, 396-403, doi:10.1097/mpg.0000000000001515.
128. Greuter, T.; Michel, M.C.; Thomann, D.; Weigmann, H.; Vavricka, S.R. Randomized, Placebo-Controlled, Double-Blind and Open-Label Studies in the Treatment and Prevention of Acute Diarrhea With Enterococcus faecium SF68. *Frontiers in Medicine* **2020**, *7*, doi:10.3389/fmed.2020.00276.
129. Hariharan, D.; Balasubramanian, L.; Kannappan, V.; Veluswami, G. Probiotic supplementation in VLBW preterm infants improves feeding tolerance and reduces risk of gram negative sepsis. *Journal of pediatric gastroenterology and nutrition. Conference: 49th annual meeting of the european society for paediatric gastroenterology, hepatology and nutrition, ESPGHAN 2016. Athens greece. Conference start: 20160525. Conference end: 20160528. Conference publication: (var.pagings)* **2016**, *62*, 655, doi:10.1097/01.mpg.0000484500.48517.e7.
130. Hartel, C.; Pagel, J.; Rupp, J.; Bendiks, M.; Guthmann, F.; Rieger-Fackeldey, E.; Heckmann, M.; Franz, A.; Schiffmann, J.H.; Zimmermann, B.; et al. Prophylactic use of Lactobacillus acidophilus/Bifidobacterium infantis probiotics and outcome in very low birth weight infants. *J Pediatr* **2014**, *165*, 285-289.e281, doi:10.1016/j.jpeds.2014.04.029.
131. Hartel, C.; Pagel, J.; Spiegler, J.; Buma, J.; Henneke, P.; Zemlin, M.; Viemann, D.; Gille, C.; Gehring, S.; Frommhold, D.; et al. Lactobacillus acidophilus/Bifidobacterium infantis probiotics are associated with increased growth of VLBWI among those exposed to antibiotics. *Sci Rep* **2017**, *7*, 5633, doi:10.1038/s41598-017-06161-8.
132. Hishiki, H.; Kawashima, T.; Tsuji, N.M.; Ikari, N.; Takemura, R.; Kido, H.; Shimojo, N. A Double-Blind, Randomized, Placebo-Controlled Trial of Heat-Killed Pediococcus acidilactici K15 for Prevention of Respiratory Tract Infections among Preschool Children. *Nutrients* **2020**, *12*, doi:10.3390/nu12071989.

133. Hojsak, I.; Mocic Pavic, A.; Kos, T.; Dumancic, J.; Kolacek, S. Bifidobacterium animalis subsp. lactis in prevention of common infections in healthy children attending day care centers - Randomized, double blind, placebo-controlled study. *Clin Nutr* **2016**, *35*, 587-591, doi:10.1016/j.clnu.2015.05.004.
134. Holscher, H.D.; Czerkies, L.A.; Cekola, P.; Litov, R.; Benbow, M.; Santema, S.; Alexander, D.D.; Perez, V.; Sun, S.; Saavedra, J.M.; et al. Bifidobacterium lactis Bb12 enhances intestinal antibody response in formula-fed infants: a randomized, double-blind, controlled trial. *JPEN J Parenter Enteral Nutr* **2012**, *36*, 106S-117S, doi:10.1177/0148607111430817.
135. Hu, X.Y.; Zhou, Y.X.; Xu, S.Z.; Lin, Y.Y. [Effects of probiotics on feeding intolerance in low birth weight premature infants]. *Zhongguo Dang Dai Er Ke Za Zhi* **2010**, *12*, 693-695.
136. Hurkala, J.; Lauterbach, R.; Radziszewska, R.; Strus, M.; Heczko, P. Effect of a Short-Time Probiotic Supplementation on the Abundance of the Main Constituents of the Gut Microbiota of Term Newborns Delivered by Cesarean Section-A Randomized, Prospective, Controlled Clinical Trial. *Nutrients* **2020**, *12*, doi:10.3390/nu12103128.
137. Irct201210209568N. The effect of probiotics in premature newborn. *http://www.who.int/trialsearch/Trial2.aspx?TrialID=IRCT201210209568N3* **2013**.
138. Irct2016061128386N. Evaluation of the effects of synbiotic supplementation on weight gain, fecal ph, acetate, lactate, calprotectin in preterm infants. *http://www.who.int/trialsearch/Trial2.aspx?TrialID=IRCT2016061128386N1* **2016**.
139. Isolauri, E.; Arvola, T.; Sutas, Y.; Moilanen, E.; Salminen, S. Probiotics in the management of atopic eczema. *Clin Exp Allergy* **2000**, *30*, 1604-1610, doi:10.1046/j.1365-2222.2000.00943.x.
140. Isrctn. The Preterm Prebiotic Study. *http://www.who.int/trialsearch/Trial2.aspx?TrialID=ISRCTN77444690* **2005**.
141. Isrctn. The role of pre- and probiotics in infections in term infants (De role van pre- en probiotica in infecties in a terme geboren kinderen). *http://www.who.int/trialsearch/Trial2.aspx?TrialID=ISRCTN65140085* **2008**.
142. Isrctn. Study to investigate the nutritional efficacy and tolerance of an infant formula with an added synbiotic mixture in infants. *http://www.who.int/trialsearch/Trial2.aspx?TrialID=ISRCTN23993517* **2012**.
143. Jones, M.L.; Martoni, C.J.; Tamber, S.; Parent, M.; Prakash, S. Evaluation of safety and tolerance of microencapsulated Lactobacillus reuteri NCIMB 30242 in a yogurt formulation: a randomized, placebo-controlled, double-blind study. *Food Chem Toxicol* **2012**, *50*, 2216-2223, doi:10.1016/j.fct.2012.03.010.
144. Jones, R.B.; Alderete, T.L.; Martin, A.A.; Geary, B.A.; Hwang, D.H.; Palmer, S.L.; Goran, M.I. Probiotic supplementation increases obesity with no detectable effects on liver fat or gut microbiota in obese Hispanic adolescents: a 16-week, randomized, placebo-controlled trial. *Pediatr Obes* **2018**, *13*, 705-714, doi:10.1111/ijpo.12273.
145. Jprn, U. The effect of Bifidobacterium breve on immunologic parameters in low birth weight infants: an Open-label non-randomized controlled trial. *http://www.who.int/trialsearch/Trial2.aspx?TrialID=JPRN-UMIN000016663* **2015**.
146. Kara, S.S.; Volkan, B.; Erten, I. Lactobacillus rhamnosus GG can protect malnourished children. *Benef Microbes* **2019**, *10*, 237-244, doi:10.3920/bm2018.0071.

147. Kerac, M.; Bunn, J.; Seal, A.; Thindwa, M.; Tomkins, A.; Sadler, K.; Bahwere, P.; Collins, S. Probiotics and prebiotics for severe acute malnutrition (PRONUT study): a double-blind efficacy randomised controlled trial in Malawi. *Lancet* **2009**, *374*, 136-144, doi:10.1016/s0140-6736(09)60884-9.
148. Kianifar, H.R.; Ahanchian, H.; Safarian, M.; Javid, A.; Farsad-Naeimi, A.; Jafari, S.A.; Kiani, M.A.; Dahri, M. Effects of synbiotics on anthropometric indices of obesity in children: A randomized double-blind placebo-controlled pilot study. *Topics in Clinical Nutrition* **2018**, *33*, 118-126, doi:10.1097/TIN.0000000000000134.
149. Kim, J.Y.; Kwon, J.H.; Ahn, S.H.; Lee, S.I.; Han, Y.S.; Choi, Y.O.; Lee, S.Y.; Ahn, K.M.; Ji, G.E. Effect of probiotic mix (Bifidobacterium bifidum, Bifidobacterium lactis, Lactobacillus acidophilus) in the primary prevention of eczema: a double-blind, randomized, placebo-controlled trial. *Pediatr Allergy Immunol* **2010**, *21*, e386-393, doi:10.1111/j.1399-3038.2009.00958.x.
150. Kitajima, H.; Sumida, Y.; Tanaka, R.; Yuki, N.; Takayama, H.; Fujimura, M. Early administration of Bifidobacterium breve to preterm infants: randomised controlled trial. *Arch Dis Child Fetal Neonatal Ed* **1997**, *76*, F101-107, doi:10.1136/fn.76.2.f101.
151. Kon, I.Y.; Gmoshinskaya, M.V.; Safronova, A.I.; Alarcon, P.; Vandenplas, Y. Growth and Tolerance Assessment of a Lutein-fortified Infant Formula. *Pediatr Gastroenterol Hepatol Nutr* **2014**, *17*, 104-111, doi:10.5223/pghn.2014.17.2.104.
152. Kuitunen, M.; Kukkonen, K.; Juntunen-Backman, K.; Korpela, R.; Poussa, T.; Tuure, T.; Haahtela, T.; Savilahti, E. Probiotics prevent IgE-associated allergy until age 5 years in cesarean-delivered children but not in the total cohort. *J Allergy Clin Immunol* **2009**, *123*, 335-341, doi:10.1016/j.jaci.2008.11.019.
153. Langhendries, J.P.; Detry, J.; Van Hees, J.; Lamboray, J.M.; Darimont, J.; Mozin, M.J.; Secretin, M.C.; Senterre, J. Effect of a fermented infant formula containing viable bifidobacteria on the fecal flora composition and pH of healthy full-term infants. *J Pediatr Gastroenterol Nutr* **1995**, *21*, 177-181, doi:10.1097/00005176-199508000-00009.
154. Lazou Ahrén, I.; Berggren, A.; Teixeira, C.; Martinsson Niskanen, T.; Larsson, N. Evaluation of the efficacy of Lactobacillus plantarum HEAL9 and Lactobacillus paracasei 8700:2 on aspects of common cold infections in children attending day care: a randomised, double-blind, placebo-controlled clinical study. *European Journal of Nutrition* **2020**, *59*, 409-417, doi:10.1007/s00394-019-02137-8.
155. Lee le, Y.; Bharani, R.; Biswas, A.; Lee, J.; Tran, L.A.; Pecquet, S.; Steenhout, P. Normal growth of infants receiving an infant formula containing Lactobacillus reuteri, galacto-oligosaccharides, and fructo-oligosaccharide: a randomized controlled trial. *Matern Health Neonatol Perinatol* **2015**, *1*, 9, doi:10.1186/s40748-015-0008-3.
156. Lin, H.C.; Su, B.H.; Chen, A.C.; Lin, T.W.; Tsai, C.H.; Yeh, T.F.; Oh, W. Oral probiotics reduce the incidence and severity of necrotizing enterocolitis in very low birth weight infants. *Pediatrics* **2005**, *115*, 1-4, doi:10.1542/peds.2004-1463.
157. Mai, T.T.; Thi Thu, P.; Thi Hang, H.; Trang, T.T.T.; Yui, S.; Shigehisa, A.; Tien, V.T.; Dung, T.V.; Nga, P.B.; Hung, N.T.; et al. Efficacy of probiotics on digestive disorders and acute respiratory infections: a controlled clinical trial in young Vietnamese children. *European Journal of Clinical Nutrition* **2020**, doi:10.1038/s41430-020-00754-9.

158. Maldonado-Lobón, J.A.; Flores-Rojas, K.; Gil-Campos, M.; Maldonado, J.; Valero, A.D.; Lara-Villoslada, F.; Olivares, M.; Fonollá, J. Study of long-term effects of infant formula one consumer supplemented with lactobacillus fermentum CECT5716. *Acta Physiologica* **2014**, *212*, 89, doi:10.1111/apha.12388.
159. Maldonado-Lobon, J.A.; Gil-Campos, M.; Maldonado, J.; Lopez-Huertas, E.; Flores-Rojas, K.; Valero, A.D.; Rodriguez-Benitez, M.V.; Banuelos, O.; Lara-Villoslada, F.; Fonolla, J.; et al. Long-term safety of early consumption of Lactobacillus fermentum CECT5716: A 3-year follow-up of a randomized controlled trial. *Pharmacol Res* **2015**, *95-96*, 12-19, doi:10.1016/j.phrs.2015.01.006.
160. Manzoni, P.; Stolfi, I.; Cattani, S.; Messner, H.; Laforgia, N.; Romeo, M.G.; Decembrino, L.; Rinaldi, M.; Gallo, E.; Stronati, M.; et al. Bovine lactoferrin supplementation prevents invasive fungal infections in preterm VLBW neonates: Data from a multicenter, Randomized, Double-blind, Placebo-controlled study. *Early Human Development* **2011**, *87*, S92-S93, doi:10.1016/j.earlhumdev.2010.12.031.
161. Marissen, J.; Haiss, A.; Meyer, C.; Van Rossum, T.; Bunte, L.M.; Frommhold, D.; Gille, C.; Goedicke-Fritz, S.; Gopel, W.; Hudalla, H.; et al. Efficacy of Bifidobacterium longum, B. infantis and Lactobacillus acidophilus probiotics to prevent gut dysbiosis in preterm infants of 28+0-32+6 weeks of gestation: a randomised, placebo-controlled, double-blind, multicentre trial: the PRIMAL Clinical Study protocol. *BMJ Open* **2019**, *9*, e032617, doi:10.1136/bmjopen-2019-032617.
162. Materna, L. Evaluation of the Effect of Milk Based Infant Formula Supplemented Either With Probiotic Microorganisms and/or With Prebiotic on the Intestinal Microflora During the First 4 Months of Life of Healthy, Full Term Infants and it's Long Term Effect on Morbidity up to the Age of 9 Months. *Clinicaltrials.gov identifier: NCT00836771* **2010**.
163. Merenstein, D.; Murphy, M.; Fokar, A.; Hernandez, R.K.; Park, H.; Nsouli, H.; Sanders, M.E.; Davis, B.A.; Niborski, V.; Tondu, F.; et al. Use of a fermented dairy probiotic drink containing Lactobacillus casei (DN-114 001) to decrease the rate of illness in kids: the DRINK study. A patient-oriented, double-blind, cluster-randomized, placebo-controlled, clinical trial. *Eur J Clin Nutr* **2010**, *64*, 669-677, doi:10.1038/ejcn.2010.65.
164. Merenstein, D.J.; Smith, K.H.; Scriven, M.; Roberts, R.F.; Sanders, M.E.; Petterson, S. The study to investigate the potential benefits of probiotics in yogurt, a patient-oriented, double-blind, cluster-randomised, placebo-controlled, clinical trial. *Eur J Clin Nutr* **2010**, *64*, 685-691, doi:10.1038/ejcn.2010.30.
165. Meyer, M.P.; Alexander, T. Use of the probiotic lactobacillus GG in combination with lactoferrin and improved outcomes in preterm infants. *Journal of Paediatrics and Child Health* **2015**, *51*, 69, doi:10.1111/jpc.12884-4.
166. Modi, N.; Uthaya, S.; Fell, J.; Kulinskaya, E. A randomized, double-blind, controlled trial of the effect of prebiotic oligosaccharides on enteral tolerance in preterm infants (ISRCTN77444690). *Pediatr Res* **2010**, *68*, 440-445, doi:10.1203/PDR.0b013e3181f1cd59.
167. Muraro, A.; Hoekstra, M.O.; Meijer, Y.; Lifschitz, C.; Wampler, J.L.; Harris, C.; Scalabrin, D.M.F. Extensively hydrolysed casein formula supplemented with Lactobacillus rhamnosus GG maintains hypoallergenic status: Randomised double-blind, placebo-controlled crossover trial. *BMJ Open* **2012**, *2*, doi:10.1136/bmjopen-2011-000637.

168. Nakamura, S.; Sarker, S.A.; Oku, T.; Wahed, M.A.; Wagatsuma, Y. Effect of daily intake of prebiotic (Fructooligosaccharide) on weight gain and reduction of acute diarrhea among children in a Bangladesh Urban Slum: A randomized double-masked placebo-controlled study. *Gastroenterology* **2010**, *138*, S51.
169. 2008, N. Prophylactic Probiotics in Premature Infants. Available online: (accessed on
170. Nct. Effects of a Synbiotics-containing Starter Formula on Infant Growth. *https://clinicaltrials.gov/show/NCT01010113* **2009**.
171. 2010, N. Prevention of the Minor Digestive Disorders by Lactobacillus Reuteri Supplementation. Available online: (accessed on
172. Nct. Double Blind Controlled Trial of an Extensively Hydrolyzed Formula With a Probiotic vs. an Extensively Hydrolyzed Formula Without a Probiotic. *https://clinicaltrials.gov/show/NCT01181297* **2010**.
173. University, T.W.s.M. Early Administration of Bifidobacterium to Very Low Birth Weight Infants. **2007**.
174. Nct. Probiotic Formula and Infant Growth. *https://clinicaltrials.gov/show/NCT01476397* **2011**.
175. Prophylactic Probiotics to Extremely Low Birth Weight Prematures. Available online: https://ClinicalTrials.gov/show/NCT01603368 (accessed on
176. Nct. Feeding Infant Formula With Added Probiotics and Whey Protein Concentrate. *https://clinicaltrials.gov/show/NCT01755481* **2012**.
177. Nct. Effect of Probiotic Supplementation on Immune Function in Healthy Infants. *https://clinicaltrials.gov/show/NCT01542320* **2012**.
178. Nct. Effect of Starter Formula on Infection Prevention. *https://clinicaltrials.gov/show/NCT01880970* **2013**.
179. Nct. The Effects on Growth and Tolerance of an Infant Formula Fed to Term Infants. *https://clinicaltrials.gov/show/NCT01897922* **2013**.
180. Nct. Effects of Oral Probiotic Supplementation on the Clinical Status of Very-low-birth-weight Preterm Neonates. *https://clinicaltrials.gov/show/NCT02073214* **2014**.
181. Nct. Effect of a Starter Formula With Synbiotics on Stool Microbiota in Infants After Normal or Caesarean Section Delivery. *https://clinicaltrials.gov/show/NCT02031887* **2014**.
182. Nct. The Combiotic-Study. *https://clinicaltrials.gov/show/NCT02221687* **2014**.
183. Nct. Effect of a New Infant Cereal on Weight Gain. *https://clinicaltrials.gov/show/NCT02165956* **2014**.
184. Nct. Effect of Probiotics in Reducing Infections and Allergies in Young Children During the Complementary Feeding Period. *https://clinicaltrials.gov/show/NCT02032056* **2014**.
185. Nct. Safety of Lactobacillus Reuteri in Healthy Children Ages 2 to 5 Years in Peru. *https://clinicaltrials.gov/show/NCT02124122* **2014**.
186. Nct. Severe Necrotizing Enterocolitis in Preterm Newborns <1500g Using Probiotics. *https://clinicaltrials.gov/show/NCT02226263* **2014**.
187. Nct. Effects of Bifidobacterium Longum BB536 on Incidence of Acute Diarrhea and/or Respiratory- Related Illnesses in Children. *https://clinicaltrials.gov/show/NCT02434042* **2015**.

188. Nct. Safety of Lactobacillus Reuteri in Healthy Children Aged 2-24 Months. https://clinicaltrials.gov/show/NCT02460575 **2015**.
189. Nct. The Effect of Feeding Infant Formula Containing Prebiotics and/or Probiotics. https://clinicaltrials.gov/show/NCT02948114 **2016**.
190. Nct. Prebiotic in Preterm Infants. https://clinicaltrials.gov/show/NCT03306316 **2017**.
191. Nct. Effect of Double Fortification (Iron and Zinc) in Synbiotic Milk to Under 5 Years Stunted Children Growth. https://clinicaltrials.gov/show/NCT03495401 **2018**.
192. Nct. Pilot of a Prebiotic and Probiotic Trial in Young Infants With Severe Acute Malnutrition. https://clinicaltrials.gov/show/NCT03666572 **2018**.
193. Nct. Effect of a Mixture of New Probiotic Strains in Preterm Infants. https://clinicaltrials.gov/show/NCT03701906 **2018**.
194. Nct. Randomised, Controlled Study to Assess Safety and Tolerance of Infant Formula With Prebiotics and Postbiotics in Healthy Infants. https://clinicaltrials.gov/show/NCT04015050 **2019**.
195. Nct. The EFFECT Study: probiotic and HMO Supplementation in Infants. https://clinicaltrials.gov/show/NCT03994315 **2019**.
196. Nct. Effect of L. Rhamnosus Yoba on RTI and Other Health Outcomes Among Children (3-6 Years) in Uganda. https://clinicaltrials.gov/show/NCT04144491 **2019**.
197. Beneo-Institute; Ordesa, L.; Union, E. Long-term Safety and Efficacy of Prebiotic Enriched Infant Formula. Available online: (accessed on
198. Nopchinda, S.; Varavithya, W.; Phuapradit, P.; Sangchai, R.; Suthutvoravut, U.; Chantraruksa, V.; Haschke, F. Effect of bifidobacterium Bb12 with or without Streptococcus thermophilus supplemented formula on nutritional status. *J Med Assoc Thai* **2002**, *85 Suppl 4*, S1225-1231.
199. Ntr. Infant formulas with prebiotics and synbiotics in Vietnam. http://www.who.int/trialsearch/Trial2.aspx?TrialID=NTR1359 **2008**.
200. Ntr. Combining prebiotics or/and probiotics to improve the immunity of Children. http://www.who.int/trialsearch/Trial2.aspx?TrialID=NTR1234 **2008**.
201. Ntr. Fermented infant formula with prebiotics study. http://www.who.int/trialsearch/Trial2.aspx?TrialID=NTR2521 **2010**.
202. Oberhelman, R.A.; Gilman, R.H.; Sheen, P.; Taylor, D.N.; Black, R.E.; Cabrera, L.; Lescano, A.G.; Meza, R.; Madico, G. A placebo-controlled trial of Lactobacillus GG to prevent diarrhea in undernourished Peruvian children. *J Pediatr* **1999**, *134*, 15-20, doi:10.1016/s0022-3476(99)70366-5.
203. Pastor-Villaescusa, B.; Hurtado, J.A.; Gil-Campos, M.; Uberos, J.; Maldonado-Lobón, J.A.; Díaz-Ropero, M.P.; Bañuelos, O.; Fonollá, J.; Olivares, M.; Group, t.P. Effects of Lactobacillus fermentum CECT5716 Lc40 on infant growth and health: a randomised clinical trial in nursing women. *Beneficial Microbes* **2020**, *11*, 235-244, doi:10.3920/bm2019.0180.
204. Patole, S.K.; Keil, A.D.; Nathan, E.; Doherty, D.; Esvaran, M.; Simmer, K.N.; Conway, P. Effect of Bifidobacterium breve M-16V supplementation on faecal bifidobacteria in growth restricted very preterm infants - analysis from a randomised trial. *Journal of maternal-fetal & neonatal medicine* **2016**, 1-5, doi:10.3109/14767058.2016.1147554.

205. Pecquet, S.; Steenhout, P.; Radkova, N.; Pochynok, T.; Al-Jashi, I. Growth of Infant Fed with Low Protein Acidified Formulae Containing Probiotics. *Pediatric academic societies annual meeting; 2012 april 28 - may 1; boston ma, united states* **2012**.
206. Serce Pehlevan, O.; Benzer, D.; Gursoy, T.; Karatekin, G.; Ovali, F. Synbiotics use for preventing sepsis and necrotizing enterocolitis in very low birth weight neonates: a randomized controlled trial. *Clin Exp Pediatr* **2020**, *63*, 226-231, doi:10.3345/cep.2019.00381.
207. Picaud, J.C.; Pajek, B.; Arciszewska, M.; Tarczon, I.; Escribano, J.; Porcel, R.; Adelt, T.; Hassink, E.; Rijnierse, A.; Abrahamse-Berkeveld, M.; et al. An Infant Formula with Partially Hydrolyzed Whey Protein Supports Adequate Growth and Is Safe and Well-Tolerated in Healthy, Term Infants: A Randomized, Double-Blind, Equivalence Trial. *Nutrients* **2020**, *12*, doi:10.3390/nu12072072.
208. Rinaldi, M.; Manzoni, P.; Meyer, M.; Della Casa, E.; Pugni, L.; Mosca, F.; Stolfi, I.; Messner, H.; Memo, L.; Laforgia, N.; et al. Bovine lactoferrin supplementation for prevention of necrotising enterocolitis in preterm very-low-birth-weight neonates: A randomised trial. *Early Human Development* **2012**, *88*, S102.
209. Rinne, M.M.; Gueimonde, M.; Kalliomaki, M.; Hoppu, U.; Salminen, S.J.; Isolauri, E. Similar bifidogenic effects of prebiotic-supplemented partially hydrolyzed infant formula and breastfeeding on infant gut microbiota. *FEMS Immunol Med Microbiol* **2005**, *43*, 59-65, doi:10.1016/j.femsim.2004.07.005.
210. Rodriguez-Herrera, A.; Mulder, K.; Bouritius, H.; Rubio, R.; Muñoz, A.; Agosti, M.; Lista, G.; Corvaglia, L.; Ludwig, T.; Abrahamse-Berkeveld, M.; et al. Gastrointestinal Tolerance, Growth and Safety of a Partly Fermented Formula with Specific Prebiotics in Healthy Infants: A Double-Blind, Randomized, Controlled Trial. *Nutrients* **2019**, *11*, 1530-1530, doi:10.3390/nu11071530.
211. Rojas, M.A.; Lozano, J.M.; Rojas, M.X.; Rodriguez, V.A.; Rondon, M.A.; Bastidas, J.A.; Perez, L.A.; Rojas, C.; Ovalle, O.; Garcia-Harker, J.E.; et al. Prophylactic probiotics to prevent death and nosocomial infection in preterm infants. *Pediatrics* **2012**, *130*, e1113-1120, doi:10.1542/peds.2011-3584.
212. Rubaltelli, F.; Biadaioli, R.; Dani, C. Probiotics feeding prevents necrotizing enterocolitis in preterm infants: a prospective double-blind study. *Pediatric research* **2000**, *47*, 346A.
213. Sadowska-Krawczenko, I.; Korbal, P.; Polak, A.; Wietlicka-Piszcz, M.; Szajewska, H. Ocena skuteczności Lactobacillus rhamnosus ATC A07FA w zapobieganiu martwiczego zapalenia jelit wcześniaków z bardzo małą urodzeniową masą ciała: badanie z randomizacją (wstępne wyniki). *Pediatria Polska* **2012**, *87*, 139-145, doi:https://doi.org/10.1016/S0031-3939(12)70608-X.
214. Saran, S.; Gopalan, S.; Krishna, T.P. Use of fermented foods to combat stunting and failure to thrive. *Nutrition* **2002**, *18*, 393-396, doi:10.1016/s0899-9007(01)00790-0.
215. Sari, F.N.; Eras, Z.; Dizdar, E.A.; Erdeve, O.; Oguz, S.S.; Uras, N.; Dilmen, U. Do oral probiotics affect growth and neurodevelopmental outcomes in very low-birth-weight preterm infants? *Am J Perinatol* **2012**, *29*, 579-586, doi:10.1055/s-0032-1311981.
216. Sazawal, S.; Dhingra, U.; Hiremath, G.; Sarkar, A.; Dhingra, P.; Dutta, A.; Verma, P.; Menon, V.P.; Black, R.E. Prebiotic and probiotic fortified milk in prevention of

morbidities among children: community-based, randomized, double-blind, controlled trial. *PLoS One* **2010**, *5*, e12164, doi:10.1371/journal.pone.0012164.
217. Scalabrin, D.; Harris, C.; Johnston, W.H.; Berseth, C.L. Long-term safety assessment in children who received hydrolyzed protein formulas with Lactobacillus rhamnosus GG: a 5-year follow-up. *Eur J Pediatr* **2017**, *176*, 217-224, doi:10.1007/s00431-016-2825-4.
218. Serce, O.; Gursoy, T.; Karatekin, G.; Ovali, F. Effects of prebiotic and probiotic combination on necrotizing enterocolitis and sepsis prophylaxis in very low birth weight infants. *Journal of Perinatal Medicine* **2013**, *41*, doi:10.1515/jpm-2013-2002.
219. Silva, M.R.; Dias, G.; Ferreira, C.L.; Franceschini, S.C.; Costa, N.M. Growth of preschool children was improved when fed an iron-fortified fermented milk beverage supplemented with Lactobacillus acidophilus. *Nutr Res* **2008**, *28*, 226-232, doi:10.1016/j.nutres.2008.02.002.
220. Strong, P.; Kirchoff, A.; Harris, C.; Berseth, C.L. Growth of infants fed formula with prebiotics and probiotics. *FASEB Journal* **2015**, *29*.
221. Strus, M.; Helwich, E.; Lauterbach, R.; Rzepecka-Weglarz, B.; Nowicka, K.; Wilinska, M.; Szczapa, J.; Rudnicka, M.; Slawska, H.; Szczepanski, M.; et al. Effects of oral probiotic supplementation on gut Lactobacillus and Bifidobacterium populations and the clinical status of low-birth-weight preterm neonates: a multicenter randomized, double-blind, placebo-controlled trial. *Infect Drug Resist* **2018**, *11*, 1557-1571, doi:10.2147/idr.S166348.
222. Szajewska, H.; Gyrczuk, E.; Horvath, A. Lactobacillus reuteri DSM 17938 for the management of infantile colic in breastfed infants: a randomized, double-blind, placebo-controlled trial. *J Pediatr* **2013**, *162*, 257-262, doi:10.1016/j.jpeds.2012.08.004.
223. Tewari, V.V.; Dubey, S.K.; Gupta, G. Bacillus clausii for Prevention of Late-onset Sepsis in Preterm Infants: A Randomized Controlled Trial. *J Trop Pediatr* **2015**, *61*, 377-385, doi:10.1093/tropej/fmv050.
224. Totsu, S.; Terahara, M.; Kusuda, S. Probiotics and the development of very low birthweight infants: follow-up study of a randomised trial. *BMJ Paediatr Open* **2018**, *2*, e000256, doi:10.1136/bmjpo-2018-000256.
225. Urbanska, M.; Gieruszczak-Bialek, D.; Szymanski, H.; Szajewska, H. Effectiveness of Lactobacillus reuteri DSM 17938 for the Prevention of Nosocomial Diarrhea in Children: A Randomized, Double-blind, Placebo-controlled Trial. *Pediatr Infect Dis J* **2016**, *35*, 142-145, doi:10.1097/inf.0000000000000948.
226. Vakiliamini, M.; Babaei, H.; Mohammadi, M.; Habibi, R.; Motamed, H. Intestinal Colonization Rate of Candida albicans among Low Birth Weight Neonates after Using Oral Synbiotic Supplementation: A Randomized Placebo-controlled Trial. *Iranian Journal of Neonatology IJN* **2020**, *11*, 51-56, doi:10.22038/ijn.2020.40131.1651.
227. Van Niekerk, E.; Nel, D.G.; Blaauw, R.; Kirsten, G.F. Probiotics Reduce Necrotizing Enterocolitis Severity in HIV-exposed Premature Infants. *J Trop Pediatr* **2015**, *61*, 155-164, doi:10.1093/tropej/fmv004.
228. Vandenplas, Y.; Analitis, A.; Tziouvara, C.; Kountzoglou, A.; Drakou, A.; Tsouvalas, M.; Mavroudi, A.; Xinias, I. Safety of a New Synbiotic Starter Formula. *Pediatr Gastroenterol Hepatol Nutr* **2017**, *20*, 167-177, doi:10.5223/pghn.2017.20.3.167.

229. Veereman-Wauters, G.; Staelens, S.; Van de Broek, H.; Plaskie, K.; Wesling, F.; Roger, L.C.; McCartney, A.L.; Assam, P. Physiological and bifidogenic effects of prebiotic supplements in infant formulae. *J Pediatr Gastroenterol Nutr* **2011**, *52*, 763-771, doi:10.1097/MPG.0b013e3182139f39.
230. Victoria, A.; Sevdalina, M. PROBIOTIC SUPPLEMENTATION IN PREMATURE INFANTS...XIII World Congress of Perinatal Medicine Belgrade, Serbia. October 26-29, 2017. *Journal of Perinatal Medicine* **2017**, *45*, 168-168, doi:10.1515/jpm-2017-3001.
231. Vray, M.; Hedible, B.G.; Adam, P.; Tondeur, L.; Manirazika, A.; Randremanana, R.; Mainassara, H.; Briend, A.; Artaud, C.; von Platen, C.; et al. A multicenter, randomized controlled comparison of three renutrition strategies for the management of moderate acute malnutrition among children aged from 6 to 24 months (the MALINEA project). *Trials* **2018**, *19*, 666, doi:10.1186/s13063-018-3027-3.
232. Wall, C.R.; Hill, R.J.; Lovell, A.L.; Matsuyama, M.; Milne, T.; Grant, C.C.; Jiang, Y.; Chen, R.X.; Wouldes, T.A.; Davies, P.S.W. A multicenter, double-blind, randomized, placebo-controlled trial to evaluate the effect of consuming Growing Up Milk "Lite" on body composition in children aged 12-23 mo. *Am J Clin Nutr* **2019**, *109*, 576-585, doi:10.1093/ajcn/nqy302.
233. Wu, G.; Chen, X.; Cui, N.; He, Y.; Fan, J.; Yan, D.; Zhu, X.; Zhu, X. Preventive Effect of Bifidobacterium Supplementation on Neonatal Cholestasis in Preterm Neonates with Very Low Birth Weight. *Gastroenterology Research and Practice* **2020**, *2020*, 4625315, doi:10.1155/2020/4625315.
234. Yamasaki, C.; Totsu, S.; Uchiyama, A.; Nakanishi, H.; Masumoto, K.; Washio, Y.; Shuri, K.; Ishida, S.; Imai, K.; Kusuda, S. Effect of Bifidobacterium administration on very-low-birthweight infants. *Pediatr Int* **2012**, *54*, 651-656, doi:10.1111/j.1442-200X.2012.03649.x.
235. Ye, L.L. Growth of Infants Consuming Starter Formula Containing Synbiotics. *Clinicaltrials.gov identifier: NCT01010113* **2009**.
236. Zhang, A.M.; Sun, Z.Q.; Zhang, L.M. Mosapride combined with probiotics on gastrointestinal function and growth in premature infants. *Experimental and Therapeutic Medicine* **2017**, *13*, 2675-2680, doi:10.3892/etm.2017.4340.
237. Ziegler, E.; Vanderhoof, J.A.; Petschow, B.; Mitmesser, S.H.; Stolz, S.I.; Harris, C.L.; Berseth, C.L. Term infants fed formula supplemented with selected blends of prebiotics grow normally and have soft stools similar to those reported for breast-fed infants. *J Pediatr Gastroenterol Nutr* **2007**, *44*, 359-364, doi:10.1097/MPG.0b013e31802fca8c.
